# Hydrogen-Borrowing and Interrupted-Hydrogen-Borrowing Reactions of Ketones and Methanol Catalyzed by Iridium**

**DOI:** 10.1002/anie.201410391

**Published:** 2014-12-09

**Authors:** Di Shen, Darren L Poole, Camilla C Shotton, Anne F Kornahrens, Mark P Healy, Timothy J Donohoe

**Affiliations:** Department of Chemistry, University of Oxford Chemistry Research LaboratoryMansfield Road, Oxford, OX1 3TA (UK); Novartis Institute for BioMedical Research250 Massachusetts Avenue, Cambridge, MA 02139 (USA)

**Keywords:** hydrogen borrowing, iridium, Michael addition, oxidation, synthetic methods

## Abstract

Reported herein is the use of catalytic [{Ir(cod)Cl}_2_] to facilitate hydrogen-borrowing reactions of ketone enolates with methanol at 65 °C. An oxygen atmosphere accelerates the process, and when combined with the use of a bulky monodentate phosphine ligand, interrupts the catalytic cycle by preventing enone reduction. Subsequent addition of pro-nucleophiles to the reaction mixture allowed a one-pot methylenation/conjugate addition protocol to be developed, which greatly expands the range of products that can be made by this methodology.

The use of transition metals to perform the alkylation of ketone enolates using hydrogen-borrowing reactions is an interesting and powerful alternative to enolate formation/alkylation reactions under more traditional reaction conditions (e.g. lithium amide bases coupled with alkyl halide electrophiles).[1] Recently we disclosed that rhodium was an effective metal for catalyzing the hydrogen-borrowing reactions of methanol and we developed a reaction for the methylation of ketones under relatively mild reaction conditions (carbonate base and 65 °C reaction temperature; Scheme 1).[2] Performing the reaction under an atmosphere of oxygen was an important modification resulting in higher yields and shorter reaction times (Scheme 1).

**Scheme 1:**
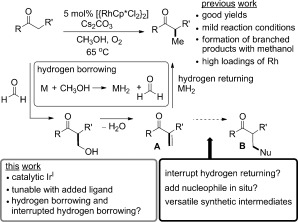
Hydrogen borrowing with methanol and the concept of interrupted hydrogen borrowing. The reduced form of the catalyst may be MH or MH_2_. Cp*=C_5_Me_5_.

Methanol is an important feedstock chemical that has been utilized in a variety of chemical transformations which involve dehydrogenation to formaldehyde.[3] It is also important to note that the use of methanol in hydrogen borrowing is exceptional in that it confers the ability (by the formation of reactive formaldehyde) to form branched products from enolates and allows the production of more complex carbonyl compounds than previous cases.[2] However, our preliminary studies revealed two areas in need of improvement. Firstly, the ketone methylation reaction required between 5 and 10 mol % of rhodium metal, thus making the sequence potentially costly. Secondly, we predicted that it was difficult for other more hindered alcohols to form branched products from ketones because of adverse steric interactions in the aldol intermediates formed en route to the alkylated products.

Our idea for producing diverse branched products was to interrupt the hydrogen-borrowing process by preventing enone reduction, thus enabling the introduction of a nucleophile in situ. Subsequent conjugate addition could follow to form a wide range of complex products. This concept is shown in Scheme 1 (**A**→**B**). In this scenario we should be able to incorporate functionality beyond a methyl group, yet harness the enhanced reactivity which comes with methanol-based hydrogen borrowing. There is a small amount of precedent for interrupting hydrogen-borrowing alkylations using either oxygen gas or a reactive alkene to intercept metal hydrides in situ, but these methods go no further than the production of unsaturated derivatives.[4]

We sought to address both issues raised above by examining a relatively inexpensive iridium(I) catalyst system which is precedented to participate in hydrogen borrowing but works best with an added ligand.[5] This requirement meant that we could adjust the balance of reactivity between the hydrogen borrowing and hydrogen returning events. Preliminary experiments with [{Ir(cod)Cl}_2_], together with a variety of phosphine ligands proved promising. Reactions of the ketone **1** with low loadings of catalyst (1–2 mol %) and different ligands are shown in Table 1. These experiments revealed that the ligand plays a crucial role in determining the products which are formed. Compare entry 1 (PPh_3_ ligand, which forms the methylated compound **2**) with entries 3 and 4 (bulky PCy_3_ or cataCXium A[6] ligands which form two interrupted intermediates, the enone **3** and methoxy addition product **4** in 59–81 % combined yield). Interestingly, the beneficial role of oxygen in enhancing the efficiency of both the methylation and methylenation reactions is also clear (entries 2 and 5).[7] Note that the combination of a hindered phosphine and an oxygen atmosphere is particularly effective in stopping enone reduction during the hydrogen-borrowing cycle (entry 4).[8] We do not yet know the precise mechanism by which the metal hydride (or dihydride) is returned to the catalytic cycle after methanol oxidation, but speculate that the recycling process involves such a metal hydride being oxidized by molecular oxygen.[9] Although the details are not yet clear, the reactivity of any iridium hydrides formed in situ is clearly influenced by the added ligand. Taken together, this set of results proves that low loadings of iridium(I) can be used with methanol in both hydrogen-borrowing and interrupted-hydrogen-borrowing chemistry depending upon the ligand added.

**Table 1:** Effect of phosphine ligand and atmosphere on product distribution

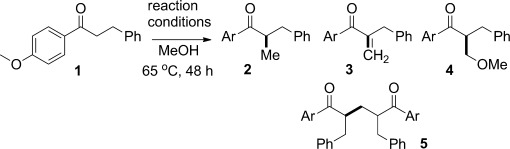						

Entry	Conditions	Product distribution [%]				
		1	2	3	4	5
1	1 mol % [{Ir(cod)Cl}_2_], 4 mol % PPh_3_, 2 equiv KOH, O_2_	–	*94*	–	–	–
2	1 mol % [{Ir(cod)Cl}_2_], 4 mol % PPh_3_, 2 equiv KOH, Ar	4	75	–	trace	trace
3	2 mol % [{Ir(cod)Cl}_2_], 8 mol % PCy_3_, 3 equiv KOH, O_2_	12	23	9	50	–
4	2 mol % [{Ir(cod)Cl}_2_], 8 mol % cataCXium A, 3 equiv KOH, O_2_	2	4	*6*	*75*	–
5	2 mol % [{Ir(cod)Cl}_2_], 8 mol % cataCXium A, 3 equiv KOH, Ar	50	29	1	13	–
6	1 mol % [{Ir(cod)Cl}_2_], 5 equiv Cs_2_CO_3_, O_2_ (24 h)	–	–	–	14	*83*

[a] Determined by ^1^H NMR spectroscopy of the material after chromatography. CataCXium A=(adamantyl)_2_PBu, cod=1,5-cyclooctadiene.

For the following investigation, we decided to concentrate on the reactions of *p*-methoxyphenyl ketones. This substrate class is an interesting ketone because there are many possibilities for the formation of functionalized esters, after hydrogen-borrowing sequences, through a regioselective Baeyer–Villiger reaction. Therefore, we examined the efficiency of the optimized interrupted hydrogen-borrowing methylenation conditions (Table 1, entry 4) with several aryl ketones and found that this was a general process which formed both the enone and methoxy addition product in good combined yield (Scheme 2). Note that control experiments have shown that the enone and methoxy adduct products are in equilibrium, and so the ratio formed in a particular reaction varies according to the precise substrate employed. Because of this reversibility, we predicted that both products would be equally useful in subsequent reactions.

**Scheme 2:**
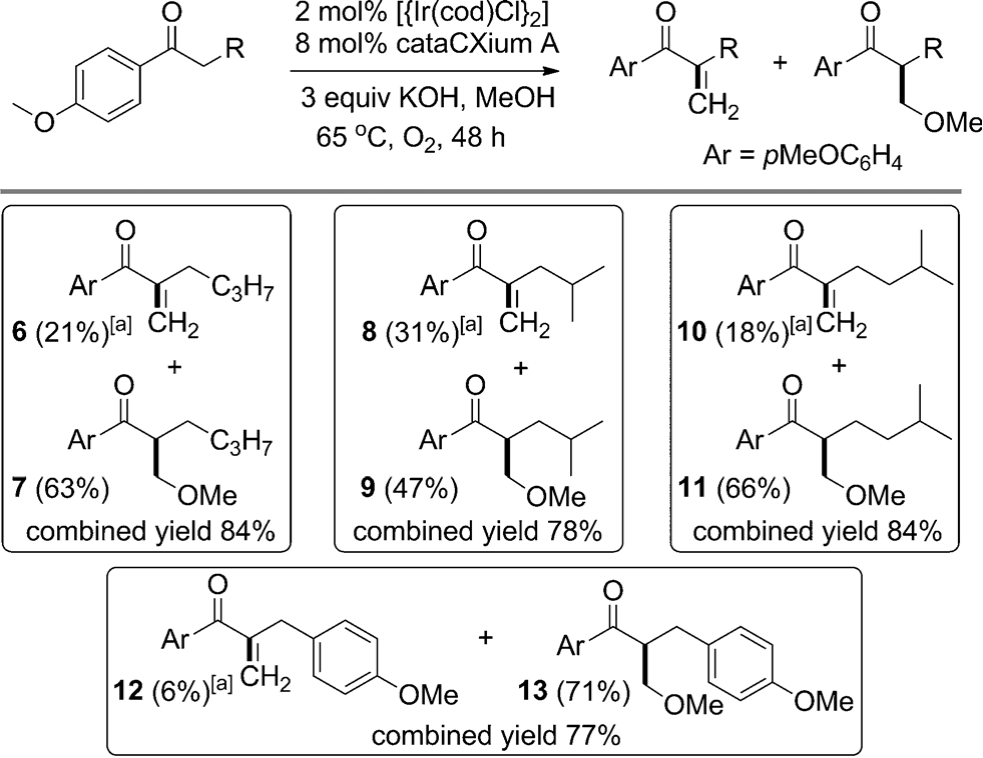
Methylenation of ketones. Yield is that of isolated products. [a] Enone contaminated with a few percent of inseparable starting material and/or methylated material. Yields adjusted accordingly.

The success of this iridium-catalyzed interrupted process allowed us to develop a one-pot methylenation/conjugate addition sequence (Scheme 3). The enone and methoxy adduct were not isolated but reacted in situ with an external nucleophile and extra base. In most cases we found it beneficial to treat the crude reaction mixture with a metal scavenging resin (SiliaMetS DMT)[10] while stirring the solution, open to the atmosphere, for 1 hour, before the addition of base and the external nucleophile. We suggest that the resin removes most of the metal catalyst from solution and prevents complications caused by methanol oxidation during the second phase of the reaction. Moreover, stirring the reaction vessel when it is open to the atmosphere concentrates the reaction and may remove unwanted formaldehyde. We found that each set of nucleophiles required a minor variation in the conditions to optimize the yields. The supplementary nucleophiles which can be added to the methylenation reaction are diverse, including nitro compounds (**14**,**15**), ketones (**16**,**17**) and even *tert*-butyl hydroperoxide to form epoxides (**18**,**19**) in good to excellent yields, and all in one pot from methylene ketones. In addition, we were also able to perform a tandem rhodium-catalyzed conjugate addition of a boroxine or boronic acid to form the doubly benzylated compound **20**, and analogue **21**, again in good overall yields for a sequence which involves multiple reaction steps.[11] For this latter type of addition, the scavenger resin was not added because it slowed down the metal-catalyzed conjugate addition. The wide variety of nucleophiles which are compatible with this protocol greatly enhance the types of products which can be accessed directly from ketones through a hydrogen-borrowing-based methodology.

**Scheme 3:**
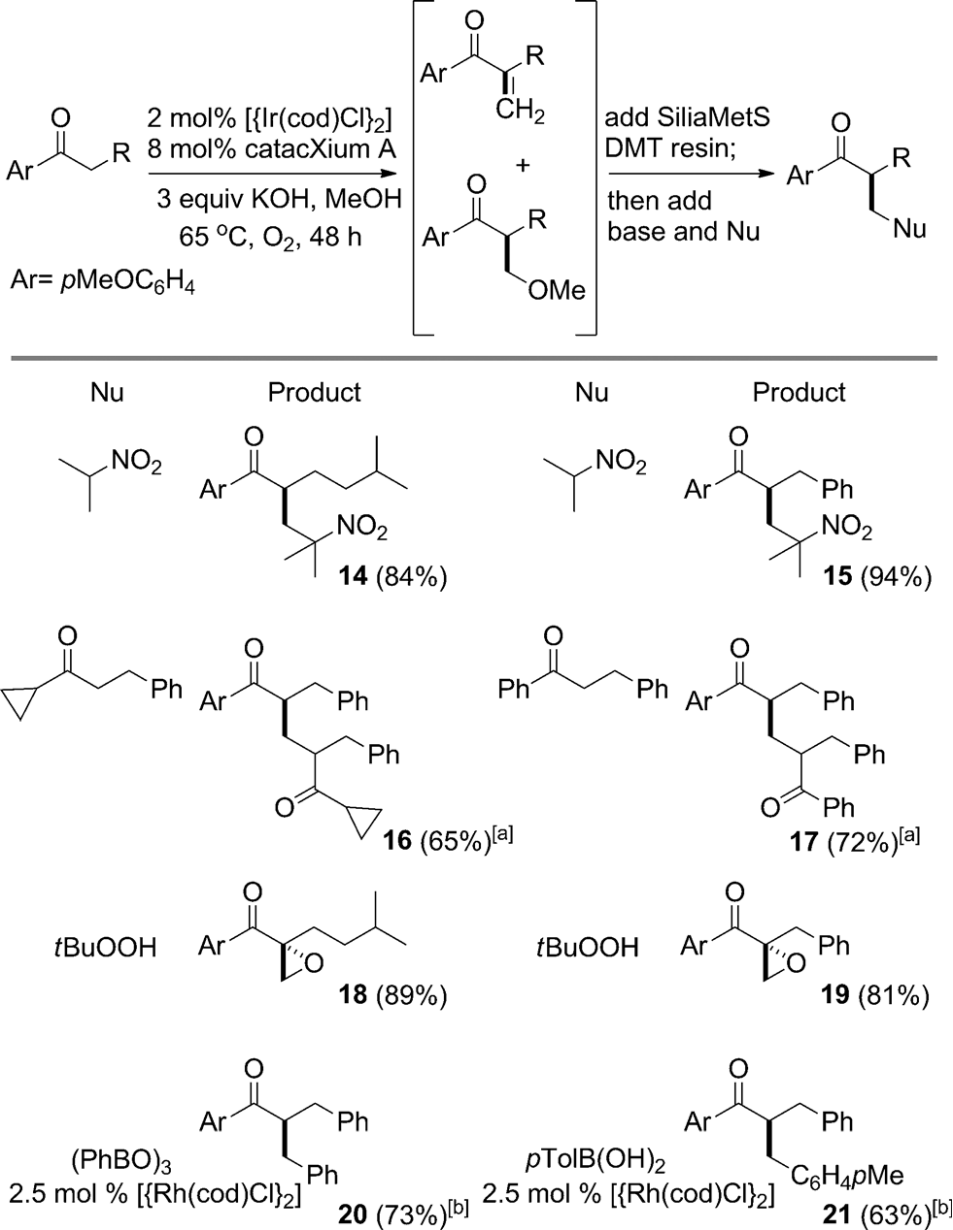
One-pot methylenation/conjugate addition of aryl ketones. Yield is that of isolated products. [a] Reaction run under an atmosphere of oxygen. [b] SiliaMetS DMT scavenger resin not added to the reaction mixture before addition of nucleophile. Reaction run under argon.

Given the propensity of iridium to participate in methanol hydrogen-borrowing processes,[12] we also examined the alternative catalytic system shown in entry 1 of Table 1 (Ph_3_P, O_2_ atmosphere), to improve our recently reported enolate methylation reaction, (Scheme 4, methylation). The outcome was a broadly applicable methylation reaction of alkyl ketones using lower loadings of iridium (2 mol % metal) compared to rhodium (10 mol % metal).

**Scheme 4:**
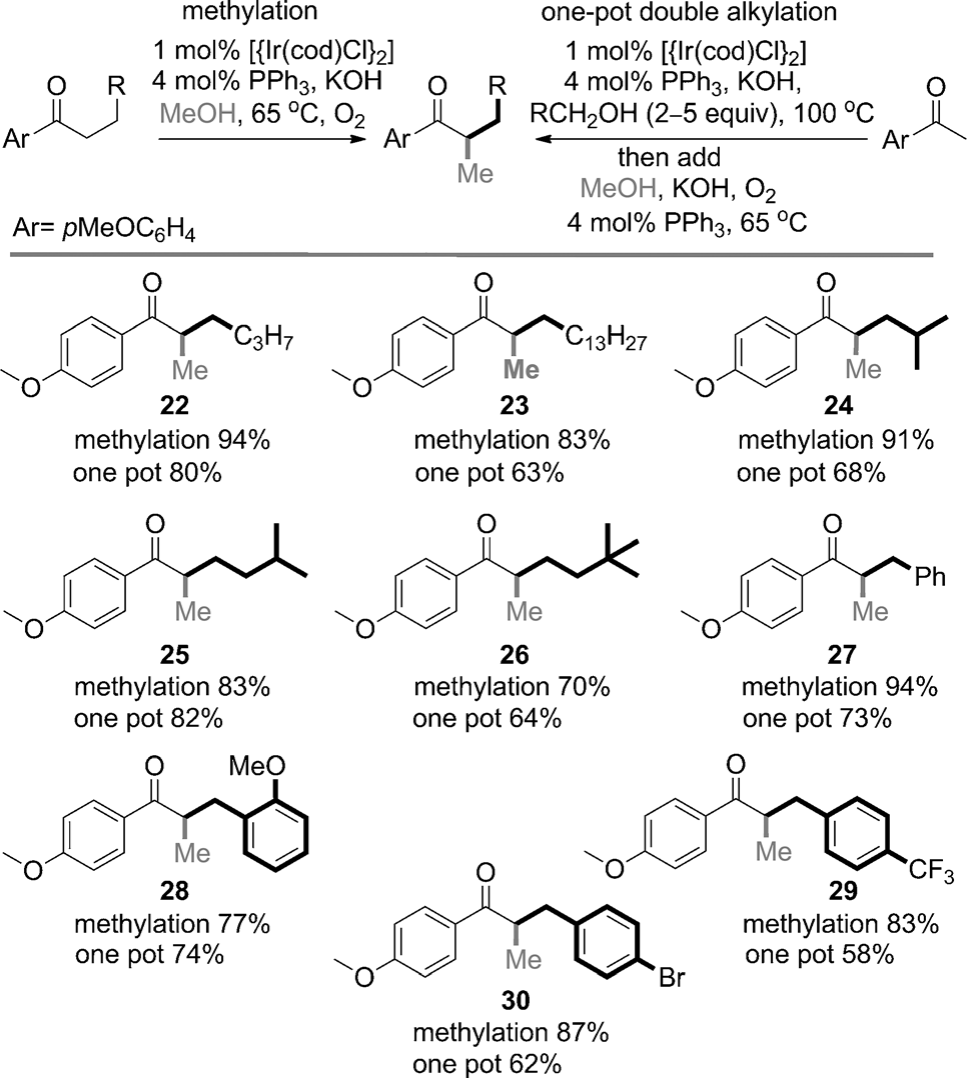
Iridium-catalyzed methylation and double alkylation of *p*-methoxyphenyl-substituted aryl ketones. The KOH loading was dependent on the substrate (see the Supporting Information for details). Yield is that of isolated products.

This procedure was then extended to a one-pot double alkylation of *p*-methoxyacetophenone with a primary alcohol at 100 °C (conditions developed by Ishii to form a monoalkylated ketone exclusively).[5] The subsequent methylation was accomplished by cooling to the reaction mixture to 65 °C and adding methanol, Ph_3_P, KOH, and a balloon of oxygen (**22**–**30**; Scheme 4). A variety of different alcohols were subjected to this regimen, thus demonstrating a useful double-alkylation reaction using 2 mol % of iridium to perform both carbon–carbon bond-forming steps in good overall yields.

Finally, our goal was to showcase the synthetic utility of the intermediates provided by this methodology. In this regard, the *p*MeOC_6_H_4_ group is an ideal candidate for regioselective migration during a Baeyer–Villiger oxidation.[13] Scheme 5 shows that a variety of different esters (**31**–**36**) can be made by reaction with *m*CPBA, including those armed with a reactive epoxide functionality. This reaction expands the range of the products which can be formed, solves the problem of esters being unstable to the basic methanol conditions, and introduces the potential for many additional reactions of the more oxidized carbonyl group in the product. Curiously, the ketone **15**, bearing a nitro group, proved to be extremely resistant to such oxidation under a variety of reaction conditions.

**Scheme 5:**
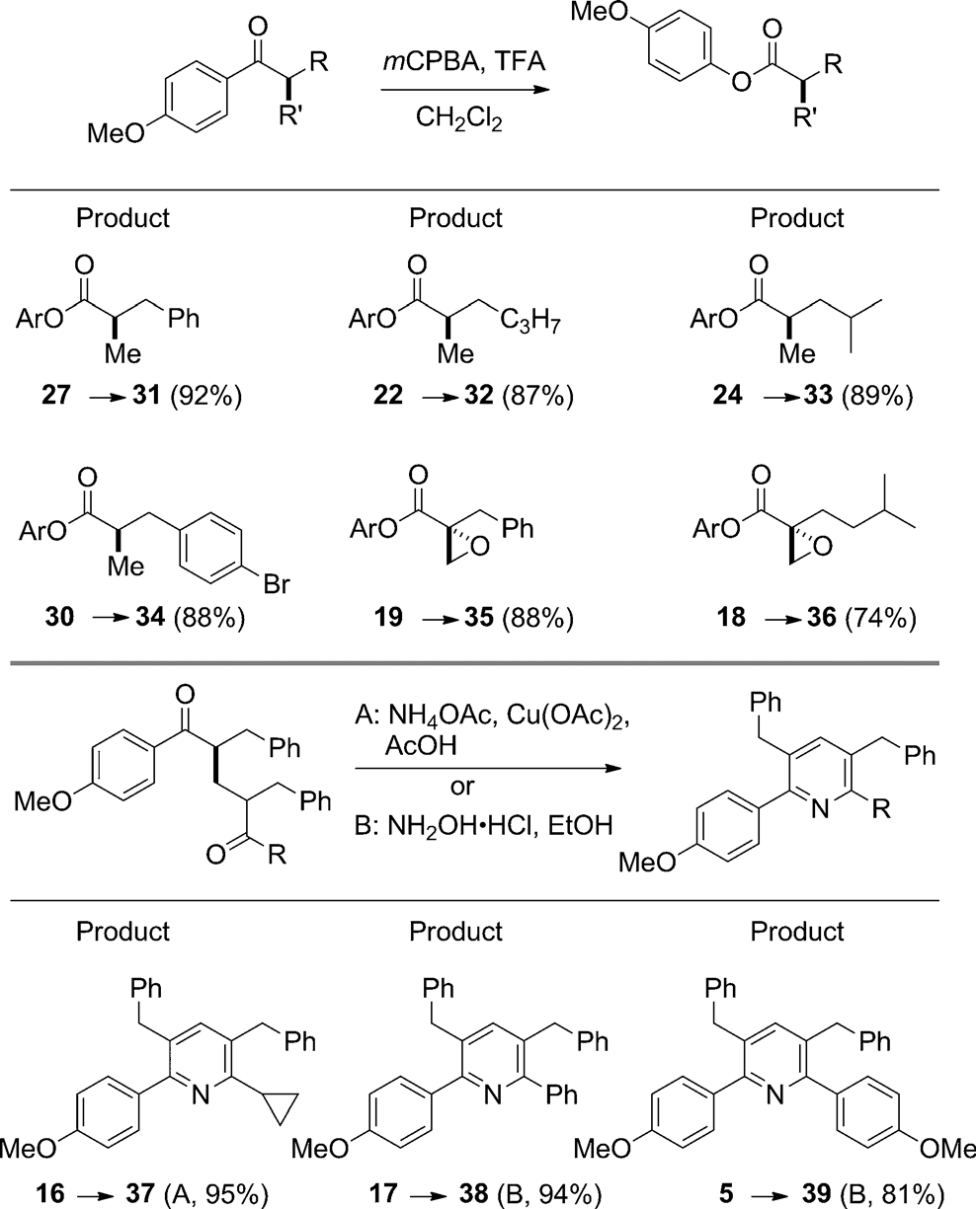
Regioselective Baeyer–Villiger and aromatizaton reactions of the aryl ketone products. Yield is that of isolated products. Baeyer–Villiger products formed with ≥20:1 regioselectivity in each case. *m*CPBA=*m*-chloroperbenzoic acid, TFA=trifluoroacetic acid.

In addition, we examined the oxidative (and aromatizing) transformation of the three 1,5-diketone derivatives made in this work (Scheme 5).[14] Reaction of the compounds **5**, **16**, and **17** with either ammonium acetate and Cu^II^ or hydroxylamine hydrochloride formed the corresponding tetrasubstituted pyridines in excellent yields.

To conclude, we have shown that [{Ir(cod)Cl}_2_] is an excellent catalyst for enolate methylenation and methylation reactions using methanol and low loadings of metal at relatively low temperatures. The combination of a sterically hindered phosphine ligand and O_2_ interrupts the hydrogen-borrowing reaction sequence and prevents enone reduction, therefore enabling the addition of a nucleophile to the unsaturated intermediates formed in situ. The synthetic utility of the products can be enhanced further by performing a regioselective Baeyer–Villiger reaction which gives access to products at the carboxylic acid oxidation state.

## Supporting Information

As a service to our authors and readers, this journal provides supporting information supplied by the authors. Such materials are peer reviewed and may be re-organized for online delivery, but are not copy-edited or typeset. Technical support issues arising from supporting information (other than missing files) should be addressed to the authors.

miscellaneous_information
