# Supplementary material for: Hydrogen-Borrowing and Interrupted-Hydrogen-Borrowing Reactions of Ketones and Methanol Catalyzed by Iridium
Source: Angew Chem Int Ed Engl. 2014 Dec 9;54(5):1642–5. doi: 10.1002/anie.201410391 (PMC4344817; doi:10.1002/anie.201410391)

Angewandte Chemie
Eine Zeitschrift der Gesellschaft Deutscher Chemiker

Supporting Information
© Wiley-VCH 2015
69451 Weinheim, Germany

Hydrogen-Borrowing and Interrupted-Hydrogen-Borrowing Reactions of Ketones and Methanol Catalyzed by Iridium**

*Di Shen, Darren L. Poole, Camilla C. Shotton, Anne F. Kornahrens, Mark P. Healy, and Timothy J. Donohoe**

anie_201410391_sm_miscellaneous_information.pdf

Supporting Information

Contents

I. General Experimental **2**

II. General Procedure A (Ir-catalyzed alkylation) **2**

III. General Procedure B (Ir-catalyzed methylation) **2**

IV. General Procedure C (Ir-catalyzed methylenation) **2**

V. General Procedure D (Baeyer Villiger oxidation) **3**

VI. General Procedure E (Pyridine formation using $NH_2OH{\cdot}HCl$) **3**

VII. General Procedure F (Pyridine formation using NH_4OAc) **3**

VIII. Compound Characterization **3**

IX. Spectral Data **22**

D. Shen, D. L. Poole, C. C. Shotton, A. F. Kornahrens, Prof. T. J. Donohoe, Department of Chemistry, University of Oxford, Chemistry Research Laboratory, Mansfield Road, Oxford, OX1 3TA (UK),

E-mail: timothy.donohoe@chem.ox.ac.uk

Dr M. Healy, Novartis Institute for BioMedical Research, 250 Massachusetts Avenue, Cambridge, MA 02139 (USA)

I. General Experimental

All solvents and reagents were used as commercially supplied without further purification unless otherwise stated. Anhydrous CH_2Cl_2, PhMe, and tetrahydrofuran (THF) were dried by filtration through an activated alumina purification column. Petrol refers to petroleum ether in the boiling range 40–60 °C. Flash column chromatography (FCC) was performed using Merck Kieselgel 60 (40–63 μm). ^{1}H nuclear magnetic resonance spectra (NMR) were recorded on a Bruker DPX200 (200 MHz), Bruker AV400 (400 MHz) or Bruker AVII500 (500 MHz). ^{13}C NMR spectra were recorded on a Bruker AV400 (101 MHz) or AVII500 (126 MHz) as stated. ^{19}F NMR spectra are recorded on a Bruker AV400 (376 MHz) and are externally calibrated to $CFCl_3$. Chemical shifts are reported relative to residual solvent peaks. Coupling constants are quoted to the nearest 0.1 Hz for ^{1}H NMR and to the nearest 1 Hz for ^{13}C NMR. Mass spectra under the conditions of electrospray ionisation (ESI) were recorded on a Fisons Platform II. Mass spectra under the conditions of field ionisation (FI) were recorded on a Micromass LCT. Mass spectra under the conditions of chemical ionisation (CI) were recorded on a Fisons Autospec-oaTof. Fourier transform infrared spectra (FTIR) were recorded as evaporated films. Melting points (m.p) were obtained using a Leica VMTG heated-stage microscope and are uncorrected.

II. General Procedure A (Ir-catalyzed alkylation)

Following literature procedure (Iuchi, Y.; Obora, Y.; Ishii, Y. *J. Am. Chem. Soc.* **2010**, *132*, 2536) with some modification where stated: Under an open atmosphere, $[Ir(cod)Cl]_2$, KOH and PPh_3 were added to a Biotage® microwave vial equipped with a stir bar, followed by methyl ketone and alcohol. The vial was sealed with a microwave vial cap (containing a Reseal™ septa) and degassed *via* a needle with a balloon of Ar. The mixture was stirred at 100 °C for the time stated. Purification (where necessary) by FCC afforded product.

III. General Procedure B (Ir-catalyzed methylation)

Under an open atmosphere, $[Ir(cod)Cl]_2$, KOH and PPh_3 were added to a Biotage® microwave vial equipped with a stir bar, followed by “wet” methanol and starting ketone. The vial was sealed with a microwave vial cap (containing a Reseal™ septum) and a balloon of O_2 was inserted *via* a needle through the septum. The reaction was heated to 65 °C for the period of time stated. The reaction was quenched by diluting with diethyl ether and filtering through a pad of silica gel. Purification by FCC afforded product.

IV. General Procedure C (Ir-catalyzed methylenation)

Under an open atmosphere, $[Ir(cod)Cl]_2$, KOH and cataCXium® A were added to a Biotage® microwave vial equipped with a stir bar, followed by “wet” methanol and starting ketone. The vial was sealed with a microwave vial cap (containing a Reseal™ septum) and degassed *via* a needle with a balloon of O_2. The reaction was heated to 65 °C for the period of time stated with the O_2 balloon attached. The reaction was quenched by diluting with diethyl ether and filtering through a pad of silica gel. Purification by FCC afforded product. (It is important to keep the balloon properly attached. If the

O_2 atmosphere is not sufficient, reaction mixture will turn brown from green and become less effective.)

V. General Procedure D (Baeyer Villiger oxidation)

Under an open atmosphere, starting ketone, mCPBA, trifluoroacetic acid and CH_2Cl_2 were added to a Biotage® microwave vial equipped with a stir bar. The vial was sealed with a microwave vial cap (containing a Reseal™ septa) and stirred at rt for 48 h. The organic phase was washed with sat. aq. $NaHCO_3$ (3 x 10mL), extracted with CH_2Cl_2, dried over Na_2SO_4, filtered, and the solvent removed *in vacuo*. Purification by FCC afforded product.

VI. General Procedure E (Pyridine formation using $NH_2OH{\cdot}HCl$)

Under an open atmosphere, starting diketone (30 mg, 1 equiv.), hydroxylamine hydrochloride (3 equiv.) were added to a Biotage® microwave vial equipped with a stir bar, followed by ethanol (0.15 mL). The vial was sealed with a microwave vial cap (containing a Reseal™ septum) and was heated to 80 ˚C for 24 h. The reaction was quenched by adding $NaHCO_3$ (sat.), extracted with CH_2Cl_2, then washed with brine, dried over Na_2SO_4, filtered, concentrated *in vacuo*. Purification by FCC afforded product.

VII. General Procedure F (Pyridine formation using NH_4OAc)

Under an open atmosphere, starting diketone (30 mg, 1 equiv.), ammonium acetate (3 equiv.), copper (II) acetate monohydrate (2.5 equiv.) were added to a Biotage® microwave vial equipped with a stir bar, followed by acetic acid (0.16 mL). The vial was sealed with a microwave vial cap (containing a Reseal™ septum), degassed with argon and heated to 120 ˚C for 24 h. The reaction was quenched by adding ammonia solution (28%-30%, 0.7 mL), extracted with EtOAc, then washed with brine, dried over Na_2SO_4, filtered, concentrated *in vacuo*. Purification by FCC afforded product.

VIII. Compound Characterization

(±)1-(4-Methoxyphenyl)-2-methyl-3-phenylpropan-1-one, 2

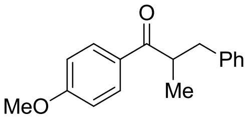

Benzylation: 4'-Methoxyacetophenone (300 mg, 2.00 mmol), $[Ir(cod)Cl]_2$ (13.4 mg, 0.0199 mmol), KOH (11.2 mg, 0.200 mmol), PPh_3 (21.0 mg, 0.0801 mmol), and benzyl alcohol (0.42 mL, 4.0 mmol) were subjected to general procedure A for 6 h. Purification by FCC (Petrol/Et_2O 9:1) afforded 1-(4-methoxyphenyl)-3-phenylpropan-1-one (**1**, 418 mg, 1.74 mmol, 87 %) as a colourless solid. **m.p.** 95-97 ^{o}C (Lit: 96-98 ^{o}C, Hajipour, A. R.; Ruoho, A. E.; Hajipour, A. R.; Khazdooz, L.; Zarei, A. *Synth.Commun.* **2009**, *39*, 2702); **^{1}H NMR** (400

MHz, $CDCl_3$) 7.94 (d, *J* 9.0 Hz, 2H), 7.16-7.33 (m, 5H), 6.92 (d, *J* 9.0 Hz, 2H), 3.86 (s, 3H), 3.05 (t, *J* 7.8 Hz, 2H), 2.25 (t, *J* 7.8 Hz, 2H); **^{13}C NMR** (101 MHz, $CDCl_3$) 197.8, 163.4, 141.4, 130.3, 129.9, 128.5, 128.4, 126.0, 113.7, 55.4, 40.1, 30.3. All spectroscopic data were consistent with those previously reported: Hajipour, A. R.; Zarei, A.; Khazdooz, L.; Ruoho, A. E. *Synth. Comm.* **2009**, *39*, 2702.

Methylation: 1-(4-Methoxyphenyl)-3-phenylpropan-1-one (**1**, 72.0 mg, 0.300 mmol), $[Ir(cod)Cl]_2$ (2.0 mg, 0.0030 mmol), KOH (33.7 mg, 0.602 mmol), PPh_3 (3.2 mg, 0.012 mmol), MeOH (1.5 mL) were subjected to general procedure B for 48 h. Purification by FCC (Petrol/Et_2O 10:1) afforded **2** (71.5 mg, 0.281 mmol, 94%) as a colorless oil.

One-pot dialkylation: To a mixture of 4'-Methoxyacetophenone (150 mg, 1.00 mmol), [Ir (cod) Cl]$_2$ (6.7 mg, 0.010 mmol), KOH (5.6 mg, 0.10 mmol) and PPh_3 (10.5 mg, 0.0400 mmol) in a Biotage® microwave vial equipped with a stir bar was added benzyl alcohol (0.21 mL, 2.0 mmol). The vial was sealed with a microwave vial cap (containing a Reseal™ septa) and degassed *via* a needle with a balloon of Ar. The mixture was stirred at 100 ^oC for 6 h before KOH (168 mg, 3.00 mmol), PPh_3 (10.5 mg, 0.0400 mmol) and MeOH (5 mL) were added, and the mixture was stirred under O_2 at 65 ^0C for 48 h. Purification by FCC (Petrol/Et2O 10:1) afforded **2** (185 mg, 0.728 mmol, 73%) as a colorless oil. **^{1}H NMR** (400 MHz, $CDCl_3$) δ 7.91 (d, *J* 8.9 Hz, 2H), 7.13-7.28 (m, 5H), 6.91 (d, *J* 8.9 Hz, 2H), 3.84 (s, 3H), 3.65-3.74 (m, 1H), 3.15 (dd, *J* 6.7, 13.6 Hz, 1H), 2.68 (dd, *J* 7.9, 13.6 Hz, 1H), 1.19 (d, *J* 6.9 Hz, 3H); **^{13}C NMR** (101 MHz, $CDCl_3$) δ 202.3, 163.4, 140.2, 130.6, 129.3, 129.1, 128.4, 126.2, 113.8, 55.5, 42.4, 39.5, 17.6. Spectroscopic data are consistent with those previously reported: Samanta, S.; Mishra, B. K.; Pace, T. C. S.; Sathyamurthy, N.; Bohne, C.; Moorthy, J. N. *J. Org. Chem.* **2006**, *71*, 4453.

(±)2-Benzyl-3-methoxy-1-(4-methoxyphenyl)propan-1-one, 4

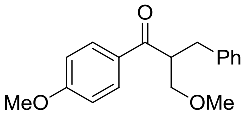

Methylenation: 1-(4-Methoxyphenyl)-3-phenylpropan-1-one (**1**, 72.0 mg, 0.300 mmol), $[Ir(cod)Cl]_2$ (4.0 mg, 0.0060 mmol), KOH (50.0mg, 0.893 mmol), cataCXium® A (8.4 mg, 0.024 mmol), MeOH (3.0 mL) were subjected to general procedure C for 48 h. Purification by FCC (Petrol/Et_2O 8:2) afforded **4** (64.3 mg, 0.226 mmol, 75%) as a colorless oil. **IR ν_{max}** (cm^{-1}) 2929, 1669, 1598, 1169, 842, 700; **^{1}H NMR** (500 MHz, $CDCl_3$) δ 7.89 (d, *J* 8.8 Hz, 2H), 7.25-7.15 (m, 5H), 6.89 (d, *J* 9.0 Hz, 2H), 3.98 (td, *J* 7.1, 1.9 Hz, 1H), 3.84 (s, 3H), 3.72 (dd, *J* 9.0, 7.4 Hz, 1H), 3.51 (dd, *J* 9.0, 5.4 Hz, 1H), 3.28 (s, 3H), 3.06 (dd, *J* 13.8, 7.5 Hz, 1H), 2.88 (dd, *J* 13.8, 6.7 Hz, 1H); **^{13}C NMR** (126 MHz, $CDCl_3$) δ 200.5, 163.5, 139.3, 130.7, 130.4, 129.0, 128.4, 126.3, 113.7, 73.7, 59.1, 55.4, 48.3, 35.5; **HRMS** (ESI^+) calculated for $[C_{18}H_{20}O_3+Na]^+$ 307.1305, found 307.1305, (Δ -0.3 ppm).

(±)-(2*RR*, 4*RS*)-2,4-Dibenzyl-1,5-bis(4-methoxyphenyl)pentane-1,5-dione, 5

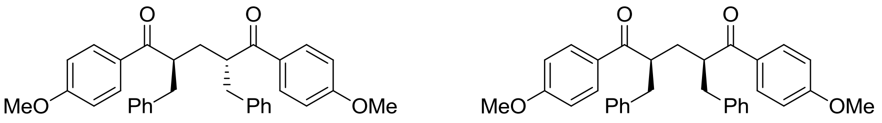

anti-XX *syn*-XX

Under an open atmosphere, $[Ir(cod)Cl]_2$ (2.0 mg, 0.0030 mmol), Cs_2CO_3 (489 mg, 1.50 mmol) were added to a Biotage® microwave vial equipped with a stir bar, followed by "wet" methanol (0.75 mL) and 1-(4-methoxyphenyl)-3-phenylpropan-1-one (**1**, 72.1 mg, 0.300 mmol). The vial was sealed with a microwave vial cap (containing a Reseal™ septum) and a balloon of O_2 was inserted *via* a needle through the septum. The reaction was heated to 65 °C for 48 h. The reaction was quenched by dilution with diethyl ether and filtering, first through a cotton filter and then through a pad of silica gel. Purification by FCC (Petrol/Et_2O 8:2) afforded **5** (61.0 mg, 83%) as a yellow oil. Dimers *anti*-**5** and *syn*-**5** were isolated as a 1:1 mixture of inseparable diastereoisomers. **IR ν_{max}** (cm^{-1}) 2933, 2839, 1667, 1598, 1510, 1246, 1170, 1030, 836, 749, 700; **^{1}H NMR** (400 MHz, $CDCl_3$) δ 7.88 (d, *J* 8.7, 4H), 7.50 (d, *J* 8.8, 4H), 7.21-7.07 (m, 16H), 6.99 (d, *J* 7.1, 4H), 6.92 (d, *J* 9.0, 4H), 6.61 (d, *J* 8.6, 4H), 3.86 (s, 6H), 3.74 (s, 6H), 3.76-3.71 (m, 2H), 3.67-3.60 (m, 2H), 3.06-3.02 (m, 2H), 3.01-2.97 (m, 2H), 2.74-2.69 (m, 2H), 2.67-2.62 (m, 2H), 2.41-2.35 (m, 1H), 2.12 (t, *J* 7.1, 2H), 1.75-1.69 (m, 1H); **^{13}C NMR** (101 MHz, $CDCl_3$) δ 202.4, 201.6, 163.6, 163.2, 139.6, 139.2, 130.8, 130.4, 130.4, 129.9, 129.0 (x2), 128.5, 128.4, 126.3, 126.7, 113.9, 113.5, 55.6, 55.3, 45.6, 45.2, 40.2, 38.5, 35.5, 34.8; **HRMS** (ESI^+) calculated for $[C_{33}H_{32}O_4+H]^+$ 493.2373, found 493.2367 (Δ –1.3 ppm).

(±)2-(Methoxymethyl)-1-(4-methoxyphenyl)hexan-1-one, 7

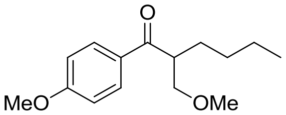

1-(4-Methoxyphenyl)hexan-1-one (**7a**, 61.8 mg, 0.300 mmol), $[Ir(cod)Cl]_2$ (4.0 mg, 0.0060 mmol), KOH (50.0mg, 0.893 mmol), cataCXium®A (8.4 mg, 0.024 mmol), MeOH (3.0 mL) were subjected to general procedure C for 48 h. Purification by FCC (Petrol/Et_2O 8:2) afforded **7** (46.9 mg, 0.188 mmol, 63%) as a colorless oil. **IR ν_{max}** (cm^{-1}) 2929, 1671, 1599, 1255, 1171, 843, 763; **^{1}H NMR** (500 MHz, $CDCl_3$) δ 7.98 (d, *J* 8.8 Hz, 2H), 6.94 (d, *J* 8.8 Hz, 2H), 3.87 (s, 3H), 3.73-3.66 (m, 2H), 3.51-3.46 (m, 1H), 3.29 (s, 3H), 1.75-1.68 (m, 1H), 1.58-1.49 (m, 1H), 1.32-1.20 (m, 4H), 0.84 (t, *J* 7.0 Hz, 3H); **^{13}C NMR** (126 MHz, $CDCl_3$) δ 201.5, 163.5, 130.7, 130.7, 113.8, 74.4, 59.1, 55.4, 46.3, 29.7, 29.6, 22.9, 13.9; **HRMS** (ESI^+) calculated for $[C_{15}H_{22}O_3+Na]^+$ 273.1461, found 273.1458, (Δ 1.3 ppm).

(±)2-(Methoxymethyl)-1-(4-methoxyphenyl)-4-methylpentan-1-one, 9

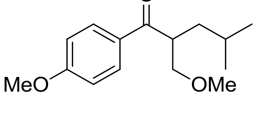

1-(4'-Methoxyphenyl)-4-methylpentan-1-one (**9a**, 61.8 mg, 0.300 mmol), $[Ir(cod)Cl]_2$ (4.0 mg, 0.0060 mmol), KOH (50.0mg, 0.893 mmol), cataCXium® A (8.4 mg, 0.024 mmol), MeOH (3.0 mL) were subjected to general procedure C for 48 h. Purification by FCC (Petrol/Et_2O 8:2) afforded **9** (35.4 mg, 0.142 mmol, 47%) as a colorless oil. **IR ν_{max}** (cm^{-1}) 2957, 1671, 1599, 1252, 1171, 842, 765; **^{1}H NMR** (400 MHz, $CDCl_3$) δ 7.99 (d, *J* 9.0 Hz, 2H), 6.94 (d, *J* 9.0 Hz, 2H), 3.87 (s, 3H), 3.82-3.77 (m, 1H), 3.67 (t, *J* 8.5 Hz, 1H), 3.46 (dd, *J* 8.8, 5.2 Hz, 1H), 3.27 (s, 3H), 1.66 (ddd, *J* 13.5, 8.1, 6.6 Hz, 1H), 1.60-1.51 (m, 1H), 1.34 (ddd, *J* 13.4, 7.5, 5.8 Hz, 1H), 0.89 (d, *J* 6.5 Hz, 3H), 0.87 (d, *J* 6.5 Hz, 3H); **^{13}C NMR** (101 MHz, $CDCl_3$) δ 201,7, 163.5, 130.8, 130.6, 113.8, 75.0, 59.1, 55.4, 44.3, 39.0, 26.1, 23.0, 22.6; **HRMS** (ESI^+) calculated for $[C_{15}H_{22}O_3+Na]^+$ 273.1461, found 273.1464, (Δ -1.0 ppm).

(±)2-(Methoxymethyl)-1-(4-methoxyphenyl)-5-methylhexan-1-one, 11

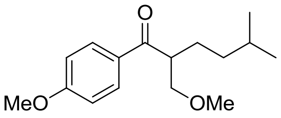

1-(4-Methoxyphenyl)-5-methylhexan-1-one (**11a**, 66.0 mg, 0.300 mmol), $Ir(cod)Cl]_2$ (4.0 mg, 0.0060 mmol), KOH (50.0mg, 0.893 mmol), cataCXium® (8.4 mg, 0.024 mmol), MeOH (3.0 mL) were subjected to general procedure C for 48 h. Purification by FCC (Petrol/Et_2O 8:2) afforded **11** (52.2 mg, 0.200 mmol, 66%) as a colorless oil. **IR ν_{max}** (cm^{-1}) 2955, 1671, 1599, 1255, 1170, 834, 760; **^{1}H NMR** (400 MHz, $CDCl_3$) δ 7.90 (d, *J* 9.0 Hz, 2H), 6.87 (d, *J* 9.0 Hz, 2H), 3.79 (s, 3H), 3.65-3.55 (m, 2H), 3.44-3.38 (m, 1H), 3.21 (s, 3H), 1.68-1.55 (m, 1H), 1.52-1.37 (m, 2H), 1.09-1.03 (m, 2H), 0.76 (d, *J* 4.4 Hz, 3H), 0.74 (d, *J* 4.4 Hz, 3H); **^{13}C NMR** (101 MHz, $CDCl_3$) δ 201.5, 163.5, 130.7, 130.6, 113.7, 74.5, 59.1, 55.4, 46.5, 36.5, 28.2, 27.8, 22.4; **HRMS** (ESI^+) calculated for $[C_{16}H_{24}O_3+Na]^+$ 287.1618, found 287.1617, (Δ 0.4 ppm).

(±)3-Methoxy-2-(4-methoxybenzyl)-1-(4-methoxyphenyl)propan-1-one, 13

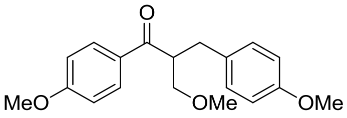

1,3-Bis(4-methoxyphenyl)propan-1-one (**13a**, 81.0 mg, 0.300 mmol), $[Ir(cod)Cl]_2$ (4.0 mg, 0.0060 mmol), KOH (50.0mg, 0.893 mmol), cataCXium® (8.4 mg, 0.024 mmol), MeOH (3.0 mL) were subjected to general procedure D for 48 h. Purification by FCC (Petrol/Et_2O 7:3) afforded **13** (67.2 mg, 0.214 mmol, 71%) as a colorless oil. **IR ν_{max}** (cm^{-1}) 2932, 2837, 1669, 1245, 840, 818; **^{1}H NMR** (400 MHz, $CDCl_3$) δ 7.89 (d, *J* 8.8 Hz, 2H), 7.08 (d, *J* 8.6 Hz, 2H), 6.89 (d, *J* 9.0 Hz, 2H), 6.77 (d, *J* 8.6 Hz, 2H), 3.97-3.90 (m, 1H), 3.84 (s, 3H), 3.75 (s, 3H), 3.70 (dd, *J* 8.8, 7.6 Hz, 1H), 3.51-3.48 (m, 1H), 3.28 (s, 3H), 3.00 (dd, *J* 13.8, 7.5, 1H), 2.81 (dd, *J* 13.8, 6.7, 1H); **^{13}C NMR** (101 MHz, $CDCl_3$) δ 200.6, 163.4, 158.1, 131.3, 130.7, 130.5, 130.0, 113.8, 113.7, 73.6, 59.1, 55.4, 55.2, 48.5, 34.7; **HRMS** (ESI^+) calculated for $[C_{19}H_{22}O_4+H]^+$ 315.1591, found 315.1585, (Δ -1.8 ppm).

(±)1-(4-Methoxyphenyl)-5-methyl-2-(2-methyl-2-nitropropyl)hexan-1-one, 14

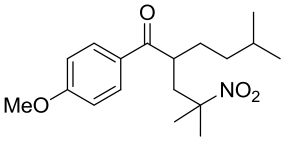

1-(4-Methoxyphenyl)-5-methylhexan-1-one (**11a**, 66.0 mg, 0.300 mmol). [Ir(cod)Cl]$_2$ (4.0 mg, 2 mol%), CataCXium A (8.6 mg, 8 mol%), KOH (50.0 mg, 0.900 mmol) and MeOH (3 mL) were subjected to general procedure C. After 48 h, SiliaMetS DMT (77.0 mg, 16 mol%) was added, and the resultant solution stirred at room temperature for 1 h open to the air. 2-Nitropropane (54 µL, 0.60 mmol), and KOH (34.0 mg, 0.600 mmol) were added, and the resultant solution stirred at 65 °C in a sealed tube under air for a further 14 h. The reaction was diluted with Et$_2$O, filtered for a short pad of silica, and concentrated *in vacuo*. Purification by FCC (Petrol/Et$_2$O 20:1) afforded **14** (81.0 mg, 0.250 mmol, 84%) as a colourless oil. **IR** ν_{max} (cm^{-1}) 2955, 1672, 1599, 1574, 1536, 1260, 1171; **^{1}H NMR** (400 MHz, CDCl$_3$) δ 7.93 (d, *J* 9.0 Hz, 2H), 6.96 (d, *J* 9.0 Hz, 2H), 3.88 (s, 3H), 3.31-3.24 (m, 1H), 2.68 (dd, *J* 9.6, 14.8 Hz, 1H), 2.20 (dd, *J* 0.7, 14.7 Hz, 1H), 1.65-1.54 (m, 4H), 1.48-1.37 (m, 2H), 1.35 (s, 3H), 1.22-1.05 (m, 2H), 0.82 (d, *J* 6.6 Hz, 3H), 0.80 (d, *J* 6.6 Hz, 3H); **^{13}C NMR** (101 MHz, CDCl$_3$) δ 201.0, 163.7, 130.6, 129.3, 114.0, 88.4, 55.5, 41.6, 41.3, 35.8, 32.5, 28.2, 28.0, 24.5, 22.5, 22.2; **HRMS** (ESI$^+$) calculated for [$C_{18}H_{27}NO_4$+H]$^+$ 322.2013, found 322.2001, (Δ −2.0 ppm).

(±)2-Benzyl-1-(4-methoxyphenyl)-4-methyl-4-nitropentan-1-one, 15

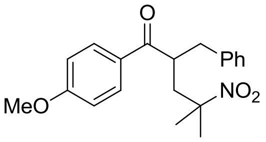

1-(4-Methoxyphenyl)-3-phenylpropan-1-one (**1**, 73.0 mg, 0.300 mmol). [Ir(cod)Cl]$_2$ (4.0 mg, 2 mol%), CataCXium A (8.6 mg, 8 mol%), KOH (50.0 mg, 0.900 mmol) and MeOH (3 mL) were subjected to general procedure C. After 48 h, SiliaMetS DMT (77.0 mg, 16 mol%) was added, and the resultant solution stirred at room temperature for 1 h open to the air. 2-Nitropropane (54 µL, 0.60 mmol), and KOH (34.0 mg, 0.60 mmol) were added, and the resultant solution stirred at 65 °C in a sealed tube under air for a further 14 h. The reaction was diluted with Et$_2$O, filtered for a short pad of silica, and concentrated *in vacuo*. Purification by FCC (Petrol/Et$_2$O20:1) afforded **15** (96.0 mg, 0.280 mmol, 94%) as a colourless oil. **IR** ν_{max} (cm^{-1}) 2935, 1671, 1598, 1535, 1260, 1235, 1169; **^{1}H NMR** (400 MHz, CDCl$_3$) δ 7.87 (d, *J* 8.9 Hz, 2H), 7.28-7.21 (m, 2H), 7.20-7.15 (m, 1H), 7.13-7.08 (m, 2H), 6.91 (d, *J* 8.9 Hz, 2H), 3.87 (s, 3H), 3.72-3.64 (m, 1H), 2.97 (dd, *J* 6.3, 13.8, 1H), 2.72-2.59 (m, 2H), 2.18 (dd, *J* 1.1, 14.7 Hz, 1H), 1.50 (s, 3H), 1.34 (s, 3H); **^{13}C NMR** (101 MHz, CDCl$_3$) δ 200.6, 163.7, 137.9, 130.6, 129.3, 129.0, 128.5, 126.7, 113.9, 88.0, 55.5, 43.4, 40.9, 40.5, 27.7, 24.9; **HRMS** (ESI$^+$) calculated for [$C_{20}H_{23}NO_4$+Na]$^+$ 364.1519 , found 364.1519, (Δ 0.1 ppm).

1-Cyclopropyl-3-phenylpropan-1-one, 16a

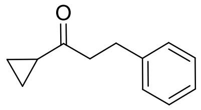

Cyclopropyl methyl ketone (0.40 mL, 4.00 mmol), $[Ir(cod)Cl]_2$ (26.8 mg, 0.0400 mmol), KOH (22.4 mg, 0.400 mmol), PPh_3 (42.0 mg, 0.160 mmol), and benzyl alcohol (2.1 mL, 20.0 mmol) were subjected to general procedure A for 4 h. Two of such reactions were combined and purified by FCC (Petrol/Et_2O 10:1) afforded **16a** (1.30 g, 7.47 mmol, 93 %) as a colourless oil. **^{1}H NMR** (400 MHz, $CDCl_3$) δ 7.33-7.21 (m, 5H), 2.98-2.89 (m, 4H), 1.94 (m, 1H), 1.05 (m, 2H), 0.89 (m, 2H); **^{13}C NMR** (101 MHz, $CDCl_3$) δ 209.9, 141.2, 128.4, 128.3, 126.0, 44.9, 29.9, 20.5, 10.7. All spectroscopic data were consistent with those previously reported: Fernμndez, R.; Ferrete, A.; Llera, J. M.; Magriz, A.; Martín-Zamora, E.; Díez, E.; Lassaletta, J. M. *Chem. Eur. J.* **2004**, *10*, 737.

(±)(2*RS*,4*SS*)-2,4-Dibenzyl-1-cyclopropyl-5-(4-methoxyphenyl)pentane-1,5-dione, 16

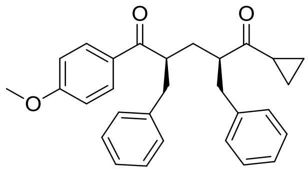

Syn-XX

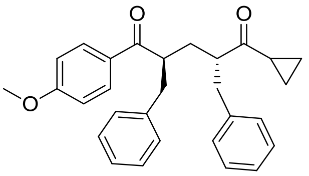

Anti-XX

Under an open atmosphere, $[Ir(cod)Cl]_2$ (13.3 mg, 0.0200 mmol), KOH (167 mg, 3 mmol) and cataCXium® (28.0 mg, 0.0800 mmol) were added to a Biotage® microwave vial equipped with a stir bar, followed by "wet" methanol (10 mL) and 1-(4-methoxyphenyl)-3-phenylpropan-1-one (**1**, 240.0 mg, 1 mmol). The vial was sealed with a microwave vial cap (containing a Reseal™ septum) and a balloon of O_2 was inserted via a needle through the septum. The reaction was heated to 65 ˚C for 48 h before being concentrated to 0.4 M (stream of Ar) and SiliaMetS DMT (257 mg, 0.16 mmol) was added. The mixture was stirred at rt open to air for 1 h before 1-cyclopropyl-3-phenylpropan-1-one (**16a**, 523 mg, 3 mmol) and KOH (167 mg, 3 mmol) were added. The vial was sealed and heated at 65 ˚C under O_2 for 21 h, and another 2 equiv. KOH (112.0 mg, 1 mmol) was added, heated for another 24 h. Crude mixture was diluted with Et_2O, filtered through SiO_2, concentrated *in vacuo*. Purification by FCC (Petrol/Et_2O 8:2) afforded **16** as a light yellow oil (277 mg, 0.650 mmol, 65%). IR ν_{max} (cm^{-1}) 3062, 3027, 2934, 2841, 1598, 1259, 1169, 699; ^{1}H NMR (500 MHz, $CDCl_3$) δ 7.89 (d, *J* = 8.8 Hz, 2 H), 7.84 (d, *J* = 9.0 Hz, 2 H), 7.30 - 7.10 (m, 18 H), 7.06 - 7.02 (m, 2 H), 6.92 (d, *J* = 8.8 Hz, 2 H), 6.88 (d, *J* = 8.8 Hz, 2 H), 3.86 - 3.82 (m, 3 H), 3.81 - 3.77 (m, 3 H), 3.77 - 3.64 (m, 2 H), 3.14 - 2.87 (m, 6 H), 2.80 - 2.69 (m, 3 H), 2.63 (dd, *J* = 6.7, 13.5 Hz, 1 H), 2.36 (td, *J* = 7.1, 14.1 Hz, 1 H), 2.14 - 2.05 (m, 1 H), 2.04 - 1.96 (m, 1 H), 1.94 - 1.84 (m, 1 H), 1.67 (td, *J* = 6.6, 13.8 Hz, 1 H), 1.57 - 1.48 (m, 1 H), 1.07 - 0.92 (m, 2 H), 0.91 - 0.79 (m, 3 H), 0.79 - 0.71 (m, 1 H), 0.65 - 0.51 (m, 2 H); ^{13}C NMR (126 MHz, $CDCl_3$) δ 213.7, 213.1, 202.0, 201.4, 163.6, 163.5, 139.6, 139.4, 139.2, 138.9, 130.7, 130.6, 130.5, 129.8, 129.0, 129.0, 129.0, 128.5, 128.4, 128.4, 126.4, 126.3, 113.9, 113.8, 55.5, 55.4, 52.8, 52.6, 45.7, 45.3, 39.9, 39.3, 38.3, 38.1, 33.8, 33.7, 20.9, 20.7, 11.7, 11.5, 11.5, 11.5 (4 aromatic carbons were not observed due to overlapping); HRMS (ESI^+) calculated for $[C_{29}H_{30}O_3+H]^+$ 427.2268, found 427.2259 (Δ –2.1 ppm).

(±)(2*SS*,4*RS*)-2,4-Dibenzyl-1-(4-methoxyphenyl)-5-phenylpentane-1,5-dione, 17

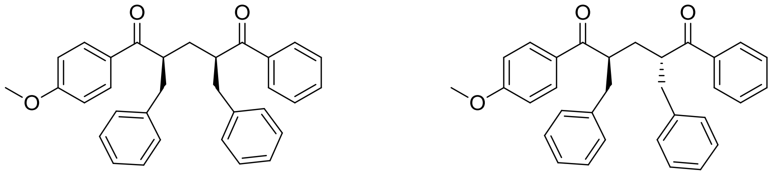

Syn-XX *Anti*-XX

Under an open atmosphere, $[Ir(cod)Cl]_2$ (13.3 mg, 0.0200 mmol), KOH (167 mg, 3 mmol) and cataCXium® A (28.0 mg, 0.0800 mmol) were added to a Biotage® microwave vial equipped with a stir bar, followed by "wet" methanol (10 mL) and 1-(4-methoxyphenyl)-3-phenylpropan-1-one (**1**, 240.0 mg, 1 mmol). The vial was sealed with a microwave vial cap (containing a Reseal™ septum) and a balloon of O_2 was inserted via a needle through the septum. The reaction was heated to 65 ˚C for 48 h before concentrated to 0.4 M (stream of Ar) and SiliaMetS DMT (257 mg, 0.16 mmol) was added. The mixture was stirred at rt open to air for 1 h before 3-phenylpropiophenone (630 mg, 3 mmol) and KOH (167 mg, 3 mmol) were added. The vial was sealed and heated at 65 ˚C for 24 h under O_2 and another 1 equiv. KOH was added (56 mg, 1 mmol), heated for another 18 h. Crude mixture was diluted with Et_2O, filtered through SiO_2, concentrated *in vacuo*. Purification by FCC (Petrol/Et_2O 8:2) afforded **17** as a light yellow oil (333 mg, 0.721 mmol, 72%). Dimers *anti*-**17** and *syn*-**17** were isolated as a 1:1 mixture of inseparable diastereoisomers. **IR ν$_{max}$** (cm^{-1}) 3061, 3027, 2923, 2851, 2361, 2341, 1674, 1599, 1259, 1245, 1171, 699; **^{1}H NMR** (500 MHz, $CDCl_3$) δ 7.90-7.87 (m, 4H), 7.58-7.51 (m, 5H), 7.46-7.43 (m, 2H), 7.39-7.35 (m, 1H), 7.22-7.08 (m, 18H), 7.01-6.99 (m, 4H), 6.94-6.92 (m, 2H), 6.64-6.63 (m, 2H), 3.87 (s, 3H), 3.84-3.66 (m, 4H), 3.74 (s, 3H), 3.07-2.98 (m, 4H), 2.75-2.63 (m, 4H), 2.43-2.38 (m, 1H), 2.15-2.12 (m, 2H), 1.77-1.72 (m, 1H); **^{13}C NMR** (126 MHz, $CDCl_3$) δ 203.9, 203.2, 202.3, 201.5, 163.6, 163.3, 139.5, 139.3, 139.1, 138.8, 137.2, 136.8, 133.1, 132.7, 130.7, 130.4, 129.8, 129.0, 128.9, 128.9, 128.7, 128.5, 128.4, 128.4, 128.4, 128.4, 128.3, 128.0, 126.4, 126.3, 126.3, 113.9, 113.5, 55.5, 55.4, 46.0, 45.6, 45.5, 45.2, 39.9, 39.9, 38.5, 38.3, 34.9, 34.5 (3 carbon peaks are not observed due to overlapping); **HRMS** (ESI$^+$) calculated for $[C_{32}H_{30}O_3+H]^+$ 463.2268, found 463.2260 (Δ –1.6 ppm).

(±) (2-Benzyloxiran-2-yl)(4-methoxyphenyl)methanone, 18

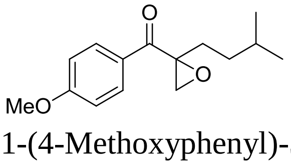

1-(4-Methoxyphenyl)-5-methylhexan-1-one (**11a**, 66.0 mg, 0.300 mmol). $[Ir(cod)Cl]_2$ (4.0 mg, 2 mol%), CataCXium A (8.6 mg, 8 mol%), KOH (50.0 mg, 0.900 mmol) and MeOH (3 mL) were subjected to general procedure C. After 48 h, SiliaMetS DMT (77.0 mg, 16 mol%) was added, and the resultant solution stirred at room temperature for 1 h open to the air. *tert*-Butylhydroperoxide (410 µL, 3.00 mmol), and Triton B (1.62 mL, 3.00 mmol) were added, and the resultant solution stirred at rt for a further 15 h. The reaction was diluted with Et_2O, filtered for a short pad of silica, and concentrated *in vacuo*. Purification by FCC (Petrol/Et_2O

20:1) afforded **18** (67.0 mg, 0.270 mmol, 89%) as a colourless oil. **IR ν$_{max}$** (cm^{-1}) 2956, 1667, 1599, 1257, 1165; **^{1}H NMR** (400 MHz, $CDCl_3$) δ 8.07 (d, *J* 9.0 Hz, 2H), 6.94 (d, *J* 9.0 Hz, 2H), 3.88 (s, 3H), 2.93 (d, *J* 5.0 Hz, 1H), 2.88 (d, *J* 5.0 Hz, 1H), 2.23 (ddd, *J* 5.1, 11.8, 14.0 Hz, 1H), 1.72 (ddd, *J* 5.1, 11.7, 14.1 Hz, 1H), 1.61-1.48 (m, 1H), 1.44-1.21 (m, 2H), 0.86 (d, *J* 6.6 Hz, 6H); **^{13}C NMR** (101 MHz, $CDCl_3$) δ 196.4, 163.8, 131.8, 127.6, 113.7, 63.6, 55.5, 50.9, 33.5, 31.3, 28.0, 22.3, 22.3; **m/z** (ESI+) 271.1 **HRMS** (ESI$^+$) calculated for $[C_{15}H_{20}O_3+H]^+$ 249.1485, found 249.1482, (Δ −1.1 ppm).

(±) (2-Benzyloxiran-2-yl)(4-methoxyphenyl)methanone, 19

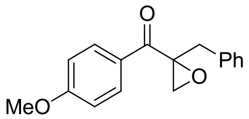

1-(4-Methoxyphenyl)-3-phenylpropan-1-one (**1**, 73.0 mg, 0.300 mmol). $[Ir(cod)Cl]_2$ (4.0 mg, 2 mol%), CataCXium A (8.6 mg, 8 mol%), KOH (50.0 mg, 0.900 mmol) and MeOH (3 mL) were subjected to general procedure C. After 48 h, SiliaMetS DMT (77.0 mg, 16 mol%) was added, and the resultant solution stirred at room temperature for 1 h open to the air. *tert*-Butylhydroperoxide (410 μL, 3.00 mmol), and Triton B (1.62 mL, 3.00 mmol) were added, and the resultant solution stirred at rt for a further 15 h. The reaction was diluted with Et_2O, filtered for a short pad of silica, and concentrated *in vacuo*. Purification by FCC (Petrol/Et_2O 20:1) afforded **19** (65.0 mg, 0.240 mmol, 81%) as a colourless oil. **IR ν$_{max}$** (cm^{-1}) 3031, 2932, 1664, 1598, 1258, 1173; **^{1}H NMR** (400 MHz, $CDCl_3$) δ 7.87 (d, *J* 9.0 Hz, 2H), 7.21-7.10 (m, 5H), 6.79 (d, *J* 9.0 Hz, 2H), 3.76 (s, 3H), 3.52 (d, *J* 14.6 Hz, 1H), 3.00 (d, *J* 14.6 Hz, 1H), 2.77 (d, *J* 5.0 Hz, 1H), 2.73 (d, *J* 5.0 Hz, 1H); **^{13}C NMR** (101 MHz, $CDCl_3$) δ 196.3, 163.7, 135.3, 131.8, 129.8, 128.3, 127.5, 126.9, 113.6, 63.5, 55.4, 50.3, 38.7; **HRMS** (ESI$^+$) calculated for $[C_{17}H_{16}O_3+Na]^+$ 291.0992, found 291.0989 (Δ 1.1 ppm).

2-Benzyl-1-(4-methoxyphenyl)-3-phenylpropan-1-one, 20

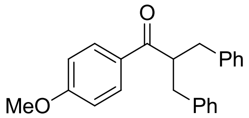

1-(4-Methoxyphenyl)-3-phenylpropan-1-one (**1**, 73.0 mg, 0.300 mmol). $[Ir(cod)Cl]_2$ (4.0 mg, 2 mol%), CataCXium A (8.6 mg, 8 mol%), KOH (50.0 mg, 0.900 mmol) and MeOH (3 mL) were subjected to general procedure C. After 48 h, the reaction was concentrated to 0.25 M (1.2 mL) by bubbling argon through the solution. Subsequently, $[Rh(cod)Cl]_2$ (3.7 mg, 2.5 mol%), 1,5-cyclooctadiene (3.7 μL, 10 mol%), KOH (50.0 mg, 0.900 mmol) and 1,3,5-triphenylboroxine (47.0 mg, 0.450 mmol) were added. The vial was sealed with a microwave vial cap (containing a Reseal™ septum) and degassed with a ballon of Ar *via* a needle inserted through the septum. The balloon was them removed, and the reaction heated at 100 °C for 6 h. The reaction was diluted with Et_2O, filtered through a short plug of SiO_2, and concentrated *in vacuo*. Purification by FCC (PhMe) afforded **20** (72.0 mg, 0.220 mmol, 73%) as a colourless oil. **IR ν$_{max}$** (cm^{-1}) 3027, 2932, 1669, 1599, 1261, 1238, 1170; **^{1}H NMR** (400

MHz, $CDCl_3$) δ 7.75 (d, *J* 9.0 Hz, 2H), 7.28-7.11 (m, 10H), 6.82 (d, *J* 9.0 Hz, 2H), 3.98 (tt, *J* 6.2 Hz, 7.9 Hz, 1H), 3.79 (s, 3H), 3.14 (dd, *J* 8.0, 13.8 Hz, 2H), 2.81 (dd, *J* 6.3, 13.8 Hz, 2H); **^{13}C NMR** (101 MHz, $CDCl_3$) δ 201.6, 163.2, 139.7, 130.4, 130.3, 129.0, 128.3, 126.2, 113.6, 55.4, 49.9, 38.3; **m/z** 363.1 **HRMS** (ESI^+) calculated for $[C_{23}H_{22}O_2+Na]^+$ 353.1512, found 353.1511, (Δ 0.3 ppm).

2-Benzyl-3-(4-bromophenyl)-1-(4-methoxyphenyl)propan-1-one, 21

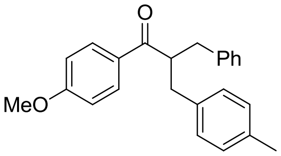

1-(4-Methoxyphenyl)-3-phenylpropan-1-one (**1**, 73.0 mg, 0.300 mmol). $[Ir(cod)Cl]_2$ (4.0 mg, 2 mol%), CataCXium A (8.6 mg, 8 mol%), KOH (50.0 mg, 0.900 mmol) and MeOH (3 mL) were subjected to general procedure C. After 48 h, the reaction was concentrated to 0.25 M (1.2 mL) by bubbling argon through the solution. Subsequently, $[Rh(cod)Cl]_2$ (3.7 mg, 2.5 mol%), 1,5-cyclooctadiene (3.7 μL, 10 mol%), KOH (50.0 mg, 0.900 mmol) and 1 *p*-tolylboronic acid (61 mg, 1.50 mmol) were added. The vial was sealed with a microwave vial cap (containing a Reseal™ septum) and degassed with a ballon of Ar *via* a needle inserted through the septum. The balloon was them removed, and the reaction heated at 100 °C for 6 h. The reaction was diluted with Et_2O, filtered through a short plug of SiO_2, and concentrated *in vacuo*. Purification by FCC (PhMe) afforded **21** (65 mg, 0.19 mmol, 63%) **IR ν_{max}** (cm^{-1}) 2906, 1689, 1598, 1510, 1237, 1169; **^{1}H NMR** (400 MHz, $CDCl_3$) δ 7.65 (d, *J* 8.8 Hz, 2 H), 7.12 - 6.98 (m, 5 H), 6.96 - 6.88 (m, 4 H), 6.71 - 6.65 (m, 2 H), 3.85 ((quin, *J* 7.0 Hz, 1 H), 3.71 - 3.59 (m, 3 H), 3.00 (td, *J* 7.8, 13.8 Hz, 2 H), 2.67 (dt, *J* 6.2, 13.8 Hz, 2 H), 2.15 (s, 3 H); **^{13}C NMR** (101 MHz, $CDCl_3$) δ 201.6, 163.2, 139.7, 136.5, 135.6, 130.4, 130.3, 129.0, 129.0, 128.8, 128.3, 126.1, 113.6, 55.3, 50.0, 38.1, 37.8, 20.9; **m/z** 363.1 **HRMS** (ESI^+) calculated for $[C_{24}H_{24}O_2+Na]^+$ 367.1669, found 367.1661 , (Δ -2.1 ppm).

(±)1-(4-Methoxyphenyl)-2-methylhexan-1-one, 22

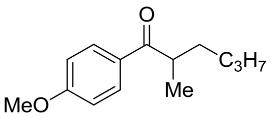

Butylation: 4'-Methoxyacetophenone (300 mg, 2.00 mmol), $[Ir(cod)Cl]_2$ (13.4 mg, 0.0199 mmol), KOH (11.2 mg, 0.200 mmol), PPh_3 (21.0 mg, 0.0801 mmol), and 1-butanol (0.92 mL, 10.0 mmol) were subjected to general procedure A for 6 h. Purification by FCC (Petrol/Et_2O 10:1) afforded 1-(4-methoxyphenyl)hexan-1-one (**7a**, 366 mg, 1.78 mmol, 89 %) as a colourless solid. **m.p.** 32-33 ^{o}C (Lit: 34-35 ^{o}C, Manchand, P. S.; Schwartz, A.; Wolff, S.; Belica, P. S.; Madan, P.; Patel, P.; Saposnik, S. J. *Heterocycles* **1993**, *35*, 1351); **^{1}H NMR** (400 MHz, $CDCl_3$) δ 7.84 (d, *J* 8.0 Hz, 2H), 6.82 (d, *J* 8.0 Hz, 2H), 3.75 (s, 3H), 2.80 (m, 2H), 1.62 (m, 2H), 1.25 (m, 4H). 0.81 (m, 3H); **^{13}C NMR** (101 MHz, $CDCl_3$) δ 199.1, 163.3, 130.3, 130.2, 113.6, 55.4, 38.2, 31.6, 24.3, 22.6, 14.0. Spectroscopic data are consistent with those previously reported: Ruan, J.; Saidi, O.; Iggo, J. A.; Xiao, J. *J. Am. Chem. Soc.* **2008**, *130*, 10510.

Methylation: 1-(4-Methoxyphenyl)hexan-1-one **7a** (61.8 mg, 0.300 mmol), [Ir(cod)Cl]$_2$ (2.0 mg, 0.0030 mmol), KOH (33.7 mg, 0.602 mmol), PPh_3 (3.2 mg, 0.012 mmol), MeOH (1.5 mL) were subjected to general procedure B for 48 h. Purification by FCC (Petrol/Et_2O 10:1) afforded **22** (62.3 mg, 0.283 mmol, 94%) as a colorless oil.

One-pot dialkylation: To a mixture of 4'-Methoxyacetophenone (150 mg, 1.00 mmol), [Ir (cod) Cl]$_2$ (6.7 mg, 0.010 mmol), KOH (5.6 mg, 0.10 mmol) and PPh_3 (10.5 mg, 0.0400 mmol) in a Biotage® microwave vial equipped with a stir bar was added 1-butanol (0.46 mL, 5.0 mmol). The vial was sealed with a microwave vial cap (containing a Reseal™ septa) and degassed *via* a needle with a balloon of Ar. The mixture was stirred at 100 ^oC for 6 h before KOH (224 mg, 4.00 mmol), PPh_3 (10.5 mg, 0.0400 mmol) and MeOH (5 mL) were added, and the mixture was stirred under O_2 at 65 ^0C for 72 h. Purification by FCC (Petrol/Et2O 10:1) afforded **22** (176 mg, 0.800 mmol, 80%) as a colorless oil. **IR** ν_{max} (cm^{-1}) 2932, 1672, 1599, 842, 734; **^{1}H NMR** (400 MHz, $CDCl_3$) δ 7.95 (d, *J* 9.0 Hz, 2H), 6.94 (d, *J* 9.0 Hz, 2H), 3.86 (s, 3H), 3.42 (sxt, *J* 6.8 Hz, 1H), 1.78 (m, 1H), 1.43 (m, 1H), 1.29 (m, 4H), 1.17 (d, J 6.8 Hz, 3H), 0.86 (m, 3H); **^{13}C NMR** (101 MHz, $CDCl_3$) δ 203.2, 163.3, 130.5, 129.8, 113.7, 55.5, 40.1, 33.7, 29.7, 22.8, 17.4, 14.0; **HRMS** (ESI$^+$) calculated for $[C_{14}H_{20}O_2 +H]^+$ 221.1536, found 221.1539, (Δ -0.3 ppm).

(±)1-(4-Methoxyphenyl)-2-methylhexadecan-1-one, 23

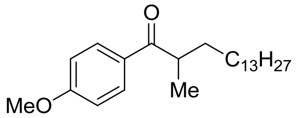

First alkylation: [IrCl(cod)]$_2$ (6.7 mg, 0.010 mmol), KOH (5.6 mg, 0.10 mmol), PPh_3 (10.5 mg, 0.040 mmol), 4-methoxyacetophenone (150 mg, 1.00 mmol), and 1-tetradecanol (1.07 g, 5.00 mmol) were subjected to general procedure A for 6 h. Purification by FCC (40:1 petrol/ether) afforded 1-(4-methoxyphenyl)hexadecan-1-one **(23a**, 272 mg, 0.79 mmol, 79%) as a colourless solid. **m.p.** 68-70 °C; **IR** ν_{max} (cm^{-1}) 3019, 2925, 2852, 1678, 1601, 1258, 1215, 1171; **^{1}H NMR** (400 MHz, $CDCl_3$) δ 7.95 (d, *J* 9.0 Hz, 2H), 6.93 (d, *J* 9.0 Hz, 2H), 3.86 (s, 3H), 2.90 (t, *J* 7.5 Hz, 2H), 1.76-1.68 (m, 2H), 1.34-1.26 (m, 24 H), 0.88 (t, *J* 6.8 Hz, 3H); **^{13}C NMR** (101 MHz, CDCl3) δ 199.2, 163.2, 130.3, 130.2, 113.6, 55.4, 38.3, 31.9, 29.7-29.4, 24.6, 22.7, 14.1; **HRMS** (ESI$^+$) calculated for $[C_{23}H_{38}O_2+H]^+$ 347.2945, found: 347.2937 (Δ−2.29 ppm).

Methylation: 1-(4-Methoxyphenyl)hexadecan-1-one (**23a**, 104 mg, 0.300 mmol), [Ir(cod)Cl]$_2$ (2.0 mg, 0.0030 mmol), KOH (50.6 mg, 0.900 mmol), PPh_3 (3.2 mg, 0.012 mmol), MeOH (1.5 mL) were subjected to general procedure B for 48 h. Purification by FCC (Petrol/Et_2O 20:1) afforded **23** (90 mg, 0.25 mmol, 83%) as a yellow solid.

One-pot dialkylation: To a mixture of 4'-Methoxyacetophenone (150 mg, 1.00 mmol), [Ir (cod) Cl]$_2$ (6.7 mg, 0.010 mmol), KOH (5.6 mg, 0.10 mmol) and PPh_3 (10.5 mg, 0.0400 mmol) in a Biotage® microwave vial equipped with a stir bar was added 1-tetradecanol (1.07 g, 5.00 mmol). The vial was sealed with a microwave vial cap (containing a Reseal™ septa) and degassed *via* a needle with a balloon of Ar. The mixture was stirred at 100 ^oC for 6 h before KOH (224 mg, 4.00 mmol), PPh_3 (10.5 mg, 0.0400 mmol) and MeOH (5 mL) were added, and the mixture was stirred under O_2 at 65 ^0C for 48 h. Purification by FCC (Petrol/Et2O 40:1) afforded **23** (251 mg, 0.63 mmol, 63%) as a yellow solid. **m.p.** 29-32 °C;

IR ν_{max} (cm^{-1}) 2923, 2852, 1675, 1600, 1575, 1509, 1461, 1308, 1257, 1233, 1170, 1034; **^{1}H NMR** (400 MHz, $CDCl_3$) δ 7.95 (d, *J* 9.0 Hz, 2H), 6.94 (d, *J* 9.0 Hz, 2H), 3.87 (s, 3H), 3.46-3.38 (m, 1H), 1.81-1.73 (m, 1H), 1.44-1.40 (m, 1H), 1.30-1.24 (m, 24H), 1.18 (d, *J* 6.8 Hz, 3H), 0.88 (t, *J* 6.8 Hz, 3H); **^{13}C NMR** (101 MHz, CDCl3) δ 203.1, 163.2 , 130.5, 129.7, 113.7, 55.4, 40.1, 33.9, 31.9, 29.8, 29.7, 29.7, 29.7, 29.7, 29.7, 29.6, 29.5, 29.4, 27.4, 22.7, 17.4, 14.1; **HRMS** (ESI^+) calculated for $[C_{24}H_{40}O_2+H]^+$ 361.3101, found: 361.3092 (Δ 2.58 ppm).

(±)1-(4-Methoxyphenyl)-2,4-dimethylpentan-1-one, 24

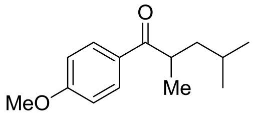

Isobutylation: $[IrCl(cod)]_2$ (13.4 mg, 0.020 mmol), KOH (22.4 mg, 0.40 mmol), PPh_3 (21.0 mg, 0.080 mmol), 4-methoxyacetophenone (300 mg, 2.0 mmol), and 2-methyl-1-propanol (0.93 mL, 10.0 mmol) were subjected to general procedure A for 6 h. Purification by FCC (9:1 petrol/ether) afforded 1-(4'-methoxyphenyl)-4-methylpentan-1-one (**9a**, 298 mg, 1.43 mmol, 72%) as a yellow oil. **^{1}H NMR** (400 MHz, $CDCl_3$) δ 7.87 (d, *J* 8.8 Hz, 2H), 6.85 (d, *J* 9.0 Hz, 2H), 3.79 (s, 3H), 2.83 (m, 2H), 1.56-1.47 (m, 3H), 0.87 (d, *J* 6.4 Hz, 6H); **^{13}C NMR** (101 MHz, $CDCl_3$) δ 199.4, 163.3, 130.3, 130.2, 113.7, 55.4, 36.3, 33.5, 27.9, 22.5. All spectroscopic data were consistent with those previously reported: Teague, S. J. *J. Org. Chem.*, **2008**, *73*, 9765.

Methylation: 1-(4'-methoxyphenyl)-4-methylpentan-1-one (**9a,** 62.4 mg, 0.300 mmol), $[Ir(cod)Cl]_2$ (2.0 mg, 0.0030 mmol), KOH (33.7 mg, 0.602 mmol), PPh_3 (3.2 mg, 0.012 mmol), MeOH (1.5 mL) were subjected to general procedure B for 48 h. Purification by FCC (Petrol/Et_2O 10:1) afforded **24** (60.9 mg, 0.274 mmol, 91%) as a colorless oil.

One-pot dialkylation: To a mixture of 4'-Methoxyacetophenone (150 mg, 1.00 mmol), [Ir (cod) Cl]$_2$ (6.7 mg, 0.010 mmol), KOH (11.2 mg, 0.20 mmol) and PPh_3 (10.5 mg, 0.0400 mmol) in a Biotage® microwave vial equipped with a stir bar was added 2-methyl-1-propanol (0.46 mL, 5.0 mmol). The vial was sealed with a microwave vial cap (containing a Reseal™ septa) and degassed *via* a needle with a balloon of Ar. The mixture was stirred at 100 ^{o}C for 6 h before KOH (168 mg, 3.00 mmol), PPh_3 (10.5 mg, 0.0400 mmol) and MeOH (5 mL) were added, and the mixture was stirred under O_2 at 65 ^{0}C for 72 h. Purification by FCC (Petrol/Et2O 10:1) afforded **24** (150 mg, 0.676 mmol, 68%) as a colorless oil. **IR** ν_{max} (cm^{-1}) 2957, 1673, 1600, 1246, 892, 803; **^{1}H NMR** (400 MHz, $CDCl_3$) δ 7.97 (m, 2H), 6.95 (m, 2H), 3.87 (s, 3H), 3.53 (sxt, *J* 6.9 Hz, 1H), 1.74-1.59 (m, 2H), 1.28(ddd, *J* 13.3, 7.2, 6.1 Hz, 1H), 1.16 (d, *J* 6.8 Hz, 3H), 0.92 (d, *J* 6.6 Hz, 3H), 0.89 (d, *J* 6.6 Hz, 3H); **^{13}C NMR** (101 MHz, $CDCl_3$) δ 203.2, 163.3, 130.5, 129.7, 113.8, 55.4, 43.0, 38.0, 25.9, 23.1, 22.4, 17.7. **HRMS** (ESI^+) calculated for $[C_{14}H_{20}O_2 +H]^+$ 221.1536, found 221.1544, (Δ -3.5 ppm).

(±)1-(4-Methoxyphenyl)-2,5-dimethylhexan-1-one, 25

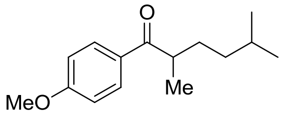

First alkylation: $[IrCl(cod)]_2$ (13.4 mg, 0.020 mmol), KOH (11.2 mg, 0.20 mmol), PPh_3 (21.0 mg, 0.080 mmol), 4-methoxyacetophenone (300 mg, 2.0 mmol), and 3-methyl-1-butanol (1.10 mL, 10.0 mmol) were subjected to general procedure A for 6 h. Purification by FCC (10:1 petrol/ether) afforded 1-(4-methoxyphenyl)-5-methylhexan-1-one (**11a**, 425 mg, 1.93 mmol, 97%) as a yellow oil. **IR** ν_{max} (cm^{-1}) 2954, 1675, 834, 807; **^{1}H NMR** (400 MHz, $CDCl_3$) δ 7.91 (d, *J* 8.6 Hz, 2H), 6.89 (d, *J* 8.3 Hz, 2H), 3.82 (s, 3H), 2.86 (t, *J* 7.5 Hz, 2H), 1.69 (m, 2H), 1.56 (m, 1H), 1.23 (m, 2H), 0.87 (d, *J* 6.6 Hz, 6H); **^{13}C NMR** (101 MHz, $CDCl_3$) δ 199.2, 163.3, 130.3, 130.2, 113.7, 55.4, 38.7, 38.5, 28.0, 22.6, 22.5; **HRMS** (ESI^+) calculated for $[C_{14}H_{20}O_2+H]^+$ 221.1536, found 221.1540, (Δ -1.4 ppm).

Methylation: 1-(4-Methoxyphenyl)-5-methylhexan-1-one (**11a,** 66.0 mg, 0.300 mmol), $[Ir(cod)Cl]_2$ (2.0 mg, 0.0030 mmol), KOH (33.7 mg, 0.602 mmol), PPh_3 (3.2 mg, 0.012 mmol), MeOH (1.5 mL) were subjected to general procedure B for 72 h. Purification by FCC (Petrol/Et_2O 10:1) afforded **25** (58.4 mg, 0.250 mmol, 83%) as a colorless oil.

One-pot dialkylation: To a mixture of 4'-Methoxyacetophenone (150 mg, 1.00 mmol), $[Ir (cod) Cl]_2$ (6.7 mg, 0.010 mmol), KOH (5.6 mg, 0.10 mmol) and PPh_3 (10.5 mg, 0.0400 mmol) in a Biotage® microwave vial equipped with a stir bar was added 3-methyl-1-butanol (0.55 mL, 5.0 mmol). The vial was sealed with a microwave vial cap (containing a Reseal™ septa) and degassed *via* a needle with a balloon of Ar. The mixture was stirred at 100 ^oC for 6 h before KOH (168 mg, 3.00 mmol), PPh_3 (10.5 mg, 0.0400 mmol) and MeOH (5 mL) were added, and the mixture was stirred under O_2 at 65 ^0C for 72 h. Purification by FCC (Petrol/Et_2O 10:1) afforded (**25**,192 mg, 0.821 mmol, 82%) as a colorless oil. **IR** ν_{max} (cm^{-1}) 2956, 1673, 843, 762; **^{1}H NMR** (400 MHz, $CDCl_3$) δ 7.95 (m, 2H), 6.94 (m, 2H), 3.86 (s, 3H), 3.39 (sxt, J 6.8 Hz, 1H), 1.78 (m, 1H), 1.45 (m, 2H), 1.20-1.14 (m, 2H), 1.18 (d, *J* 6.8, 3H), 0.85 (m, 6H); **^{13}C NMR** (101 MHz, $CDCl_3$) δ 203.2, 163.3, 130.5, 129.8, 113.7, 55.4, 40.4, 36.7, 31.8, 28.2, 22.6, 22.4, 17.5; **HRMS** (ESI^+) calculated for $[C_{15}H_{22}O_2+Na]^+$ 257.1512, found 257.1524, (Δ -4.5 ppm).

1-(4-Methoxyphenyl)-2,5,5-trimethylhexan-1-one, 26

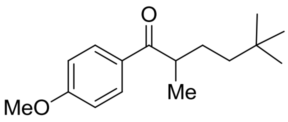

First alkylation: $[IrCl(cod)]_2$ (6.7 mg, 0.010 mmol), KOH (5.6 mg, 0.10 mmol), PPh_3 (10.5 mg, 0.040 mmol), 4-methoxyacetophenone (150 mg, 1.0 mmol), and 3,3-dimethylbutan-1-ol (0.63 mL, 5.00 mmol) were subjected to general procedure A for 6 h. Purification by FCC (40:1 petrol/ether) afforded 1-(4-methoxyphenyl)-5,5-dimethylhexan-1-one (**26a**, 234 mg, 1.00 mmol, quant.) as a yellow solid. **m.p.** 52-53 °C; **IR** ν_{max} (cm^{-1}) 3000, 2954, 2901, 2865, 1668, 1602, 1578, 1508, 1442, 1256, 1183, 1033; **^{1}H NMR** (400 MHz, $CDCl_3$) δ 7.94 (d, *J* 9.0 Hz, 2H), 6.93 (d, *J* 9.0 Hz, 2H), 3.86 (s, 3H), 2.88 (t, *J* 7.4 Hz, 2H), 1.74-1.66 (m, 2H), 1.28-1.24 (m, 2H), 0.90 (s, 9H); **^{13}C NMR** (101 MHz, $CDCl_3$) δ 199.1, 163.3, 130.2, 130.2, 113.6, 55.4, 43.9, 39.0, 30.4, 29.3, 19.8; **HRMS** (ESI^+) calculated for $[C_{15}H_{22}O_2+H]^+$ 235.1693, found 235.1689, (Δ -1.58 ppm).

Methylation: 1-(4-methoxyphenyl)-5,5-dimethylhexan-1-one (**26a,** 70.0 mg, 0.300 mmol), $[Ir(cod)Cl]_2$ (2.0 mg, 0.0030 mmol), KOH (84.2 mg, 1.500 mmol), PPh_3 (3.2 mg, 0.012

mmol), MeOH (1.5 mL) were subjected to general procedure B for 48 h. Purification by FCC (Petrol/Et_2O 40:1) afforded **26** (53 mg, 0.21 mmol, 70%) as a colorless oil.

One-pot dialkylation: To a mixture of 4'-Methoxyacetophenone (150 mg, 1.00 mmol), [Ir (cod) Cl]$_2$ (6.7 mg, 0.010 mmol), KOH (5.6 mg, 0.10 mmol) and PPh_3 (10.5 mg, 0.0400 mmol) in a Biotage® microwave vial equipped with a stir bar was added 3,3-dimethylbutan-1-ol (0.63 mL, 5.00 mmol). The vial was sealed with a microwave vial cap (containing a Reseal™ septa) and degassed *via* a needle with a balloon of Ar. The mixture was stirred at 100 ^{o}C for 6 h before KOH (336 mg, 6.00 mmol), PPh_3 (10.5 mg, 0.0400 mmol) and MeOH (5 mL) were added, and the mixture was stirred under O_2 at 65 ^{0}C for 48 h. Purification by FCC (Petrol/Et_2O 40:1) afforded **26** (158 mg, 0.64 mmol, 64%) as a colorless oil. **IR** ν_{max} (cm^{-1}) 2953, 2867, 1673, 1599, 1575, 1510, 1462, 1419, 1393, 1308, 1253, 1224, 1168, 1032; **^{1}H NMR** (400 MHz, $CDCl_3$) δ 7.95 (d, *J* 9.0 Hz, 2H), 6.94 (d, *J* 9.0 Hz, 2H), 3.87 (s, 3H), 3.39-3.30 (m, 1H), 1.82-1.73 (m, 1H), 1.44-1.35 (m, 1H), 1.26-1.11 (m, 5H), 0.85 (s, 9H); **^{13}C NMR** (101 MHz, $CDCl_3$) δ 203.1, 163.2, 130.4, 129.7, 113.7, 55.4, 41.7, 40.8, 30.2, 29.2, 28.8, 17.5; **HRMS** (ESI$^+$) calculated for $[C_{16}H_{24}O_2+H]^+$ 249.1849, found249.1845, (Δ -1.7 ppm).

(±)3-(2-Methoxyphenyl)-1-(4-methoxyphenyl)-2-methylpropan-1-one, 28

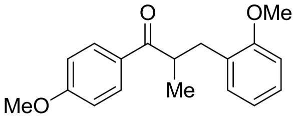

First alkylation: [IrCl(cod)]$_2$ (6.7 mg, 0.010 mmol), KOH (5.6 mg, 0.10 mmol), PPh_3 (10.5 mg, 0.040 mmol), 4-methoxyacetophenone (150 mg, 1.0 mmol), and 2-methoxybenzyl alcohol (0.27 mL, 2.00 mmol) were subjected to general procedure A for 6 h. Purification by FCC (9:1 petrol/ether) afforded 3-(2-methoxyphenyl)-1-(4-methoxyphenyl)propan-1-one (**28a**, 270 mg, 1.00 mmol, quant.) as a colorless oil. **^{1}H NMR** (400 MHz, $CDCl_3$) δ7.99 (d, *J* 9.0 Hz, 2H), 7.25-7.20 (m, 2H), 6.96-6.87 (m, 4H), 3.87 (s, 3H), 3.85 (s, 3H), 3.23 (t, *J* 7.7 Hz, 2H), 3.06 (t, *J* 7.7 Hz, 2H); **^{13}C NMR** (101 MHz, $CDCl_3$) δ 198.5, 163.2, 157.4, 130.3, 130.0, 130.0, 129.6, 127.4, 120.4, 113.6, 110.1, 55.3, 55.1, 38.5, 25.8. All spectroscopic data were consistent with those previously reported: Xu, Q.; Chen, J.; Tian, H.; Yuan, X.; Li, S.; Zhou, C.; Liu, J. *Angew. Chem. Int. Ed.* **2014**, *53*, 225.

Methylation: 3-(2-Methoxyphenyl)-1-(4-methoxyphenyl)propan-1-one (**28a,** 81 mg, 0.300 mmol), [Ir(cod)Cl]$_2$ (2.0 mg, 0.0030 mmol), KOH (84.2 mg, 1.500 mmol), PPh_3 (3.2 mg, 0.012 mmol), MeOH (1.5 mL) were subjected to general procedure B for 48 h. Purification by FCC (Petrol/Et_2O 20:1) afforded **28** (65 mg, 0.23 mmol, 77%) as a yellow solid.

One-pot dialkylation: To a mixture of 4'-Methoxyacetophenone (150 mg, 1.00 mmol), [Ir (cod) Cl]$_2$ (6.7 mg, 0.010 mmol), KOH (5.6 mg, 0.10 mmol) and PPh_3 (10.5 mg, 0.0400 mmol) in a Biotage® microwave vial equipped with a stir bar was added 2-methoxybenzyl alcohol (0.27 mL, 2.00 mmol). The vial was sealed with a microwave vial cap (containing a Reseal™ septa) and degassed *via* a needle with a balloon of Ar. The mixture was stirred at 100 ^{o}C for 6 h before KOH (336 mg, 6.00 mmol), PPh_3 (10.5 mg, 0.0400 mmol) and MeOH (5 mL) were added, and the mixture was stirred under O_2 at 65 ^{0}C for 48 h. Purification by FCC (Petrol/Et_2O 20:1) afforded **28** (210 mg, 0.74 mmol, 74%) as a yellow solid. **m.p.** 61–

63°C; **IR** ν_{max} (cm^{-1}) 2966, 2934, 2838, 1671, 1598, 1509, 1458, 1419, 1235, 1169, 1125, 1027; **^{1}H NMR** (400 MHz, $CDCl_3$) δ8.03 (d, *J* 9.0 Hz, 2H), 7.20 (td, *J* 7.7, 1.6 Hz, 1H), 7.13 (dd, 7.4, 1.6 Hz, 1H), 6.94 (d, *J* 9.0 Hz, 2H), 6.87 (m, 2H), 3.76 (s, 3H), 3.76 (s, 3H), 3.89-3.71 (m, 1H), 3.19 (dd, *J* 13.3, 5.4 Hz, 1H), 2.61 (dd, *J* 13.3, 8.7 Hz, 1H), 1.15 (d, *J* 6.8 Hz, 3H); **^{13}C NMR** (101 MHz, $CDCl_3$) δ 202.7, 163.2, 157.4, 130.6, 129.6, 128.2, 131.3, 127.5, 120.2, 113.5, 110.1, 55.4 , 55.1, 39.9, 35.2, 16.7; **HRMS** (ESI^+) calculated for $[C_{18}H_{20}O_3+H]^+$ 285.1485, found 285.1478, (Δ -2.5 ppm).

(±)1-(4-methoxyphenyl)-2-methyl-3-(4-(trifluoromethyl)phenyl)propan-1-one, 29

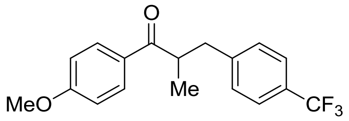

First alkylation: $[IrCl(cod)]_2$ (6.7 mg, 0.010 mmol), KOH (5.6 mg, 0.10 mmol), PPh_3 (10.5 mg, 0.040 mmol), 4-methoxyacetophenone (150 mg, 1.0 mmol), and 4-(trifluoromethyl)benzyl alcohol (0.68 mL, 5.00 mmol) were subjected to general procedure A for 6 h. Purification by FCC (9:1 petrol/ether) afforded 1-(4-methoxyphenyl)-3-(4-(trifluoromethyl)phenyl)propan-1-one (**29a**, 261 mg, 0.85 mmol, 85%) as a colorless solid. **m.p.** 56-61 °C; **IR** ν_{max} (cm^{-1}) 2938, 2844, 1676, 1601, 1325, 1169, 1117, 1068; **^{1}H NMR** (400 MHz, $CDCl_3$) δ 7.95 (d, *J* 8.8 Hz, 2H), 7.55 (d, *J* 8.0 Hz, 2H), 7.37 (d, *J* 8.0 Hz, 2H), 6.94 (d, *J* 8.8 Hz, 2H), 3.86 (s, 3H), 3.28 (t, *J* 7.5 Hz, 2H), 3.12 (t, *J* 7.5 Hz, 2H); **^{13}C NMR** (101 MHz, $CDCl_3$) 197.1, 163.5, 145.6, 130.2, 129.7, 128.8, 128.3 (q, *J* 32.3 Hz), 125.3 (q, *J* 3.7), 124.2 (q, *J* 271.8 Hz), 113.7, 55.4, 39.4, 29.9; **^{19}F NMR** (377 MHz, $CDCl_3$) δ −62.3; **HRMS** (ESI^+) calculated for $[C_{17}H_{15}O_2F_3+Na]^+$ 331.0916, found 331.0912, (Δ 1.2 ppm).

Methylation: 1-(4-Methoxyphenyl)-3-(4-(trifluoromethyl)phenyl)propan-1-one (**29a,** 93mg, 0.300 mmol), $[Ir(cod)Cl]_2$ (2.0 mg, 0.0030 mmol), KOH (50.0 mg, 0.900 mmol), PPh_3 (3.2 mg, 0.012 mmol), MeOH (1.5 mL) were subjected to general procedure B for 48 h. Purification by FCC (Petrol/Et_2O 9:1) afforded **29** (82 mg, 0.25 mmol, 83%).) as a yellow oil.

One-pot dialkylation: To a mixture of 4'-Methoxyacetophenone (150 mg, 1.00 mmol), [Ir (cod) $Cl]_2$ (6.7 mg, 0.010 mmol), KOH (5.6 mg, 0.10 mmol) and PPh_3 (10.5 mg, 0.0400 mmol) in a Biotage® microwave vial equipped with a stir bar was added 4-(trifluoromethyl)benzyl alcohol (352 mg, 5.00 mmol). The vial was sealed with a microwave vial cap (containing a Reseal™ septa) and degassed *via* a needle with a balloon of Ar. The mixture was stirred at 100 ^{o}C for 6 h before KOH (224 mg, 4.00 mmol), PPh_3 (10.5 mg, 0.0400 mmol) and MeOH (5 mL) were added, and the mixture was stirred under O_2 at 65 ^{0}C for 48 h. Purification by FCC (Petrol/Et_2O 20:1) afforded **29** (188 mg, 0.58 mmol, 58%) as a yellow oil. **IR** ν_{max} (cm^{-1}) 2970, 2936, 1672, 1599, 1323, 1260, 1235, 1164, 1114, 1066; **^{1}H NMR** (400 MHz, $CDCl_3$) δ7.91 (d, *J* 8.9 Hz, 2H), 7.51 (d, *J* 8.1 Hz, 2H), 7.31 (d, *J* 8.1 Hz, 2H), 6.93 (d, *J* 8.9 Hz, 2H), 3.86 (s, 3H), 3.78-3.68 (m, 1H), 3.23 (dd, *J* 13.7, 7.0 Hz, 1H), 2.77 (dd, *J* 13.7, 7.0 Hz, 1H), 1.22 (d, *J* 7.0 Hz, 3H); **^{13}C NMR** (101 MHz, $CDCl_3$) δ; 201.5, 163.5, 144.3, 130.5, 129.3, 129.1, 128.4 (q, *J* 32.4 Hz), 124.1 (q, *J* 272.0 Hz), 125.2 (q, *J* 3.7 Hz), 113.8, 55.4, 42.0, 39.1, 17.9; **^{19}F NMR** (377 MHz, $CDCl_3$) δ −62.3; **HRMS** (ESI^+) calculated for $[C_{18}H_{17}O_2F_3+H]^+$ 323.1253, found 323.1247, (Δ -2.1 ppm).

(±)3-(4-bromophenyl)-1-(4-methoxyphenyl)-2-methylpropan-1-one, 30

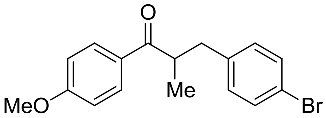

First alkylation: $[IrCl(cod)]_2$ (6.7 mg, 0.010 mmol), KOH (5.6 mg, 0.10 mmol), PPh_3 (10.5 mg, 0.040 mmol), 4-methoxyacetophenone (150 mg, 1.0 mmol), and 4-bromobenzyl alcohol (935 mg, 5.00 mmol) were subjected to general procedure A for 6 h. Purification by FCC (9:1 petrol/ether) afforded 3-(4-bromophenyl)-1-(4-methoxyphenyl)propan-1-one (**30a**, 273 mg, 0.86 mmol, 86%) as a colourless solid. **m.p.** 97-100 °C; **^{1}H NMR** (400 MHz, $CDCl_3$) δ 7.93 (d, *J* 8.9 Hz, 2H), 7.41 (d, *J* 8.4 Hz, 2H), 7.13 (d, *J* 8.4 Hz, 2H), 6.93 (d, *J* 8.9 Hz, 2H), 3.86 (s, 3H), 3.23 (t, *J* 7.7 Hz, 2H), 3.01 (t, *J* 7.7 Hz, 2H); **^{13}C NMR** (101 MHz, $CDCl_3$) δ197.3, 163.4, 140.4, 131.4, 130.2, 130.2, 129.8, 119.7, 113.7 , 55.4, 39.6, 29.6. All spectroscopic data were consistent with those previously reported: Xu, Q.; Chen, J.; Tian, H.; Yuan, X.; Li, S.; Zhou, C.; Liu, J. *Angew. Chem. Int. Ed.* **2014**, *53*, 225.

Methylation: 3-(4-Bromophenyl)-1-(4-methoxyphenyl)propan-1-one (**30a,** 96 mg, 0.300 mmol), $[Ir(cod)Cl]_2$ (2.0 mg, 0.0030 mmol), KOH (50.0 mg, 0.900 mmol), PPh_3 (3.2 mg, 0.012 mmol), MeOH (1.5 mL) were subjected to general procedure B for 48 h. Purification by FCC (Petrol/Et_2O 9:1) afforded **30** (86 mg, 0.26 mmol, 87%) as a yellow oil.

One-pot dialkylation: To a mixture of 4'-Methoxyacetophenone (150 mg, 1.00 mmol), [Ir (cod) Cl]$_2$ (6.7 mg, 0.010 mmol), KOH (5.6 mg, 0.10 mmol) and PPh_3 (10.5 mg, 0.0400 mmol) in a Biotage® microwave vial equipped with a stir bar was 4-bromobenzyl alcohol (935 mg, 5.00 mmol). The vial was sealed with a microwave vial cap (containing a Reseal™ septa) and degassed *via* a needle with a balloon of Ar. The mixture was stirred at 100 ^{o}C for 6 h before KOH (224 mg, 4.00 mmol), PPh_3 (10.5 mg, 0.0400 mmol) and MeOH (5 mL) were added, and the mixture was stirred under O_2 at 65 ^{0}C for 48 h. Purification by FCC (Petrol/Et_2O 20:1) afforded **30** (164 mg, 0.49 mmol, 62%) as a yellow oil. **IR** ν_{max} (cm^{-1}) 2968, 2932, 2839, 1671, 1598, 1574, 1509, 1488, 1458, 1259, 1169, 1030, 1011; **^{1}H NMR** (400 MHz, $CDCl_3$) δ7.91 (d, *J* 9.0 Hz, 2H), 7.37 (d, *J* 8.4 Hz, 2H), 7.07 (d, *J* 8.4 Hz, 2H), 6.92 (d, *J* 9.0 Hz, 2H), 3.86 (s, 3H), 3.72-3.63 (m, 1H), 3.11 (dd, *J* 13.7, 6.9 Hz, 1H), 2.66 (dd, *J* 13.7, 7.4 Hz, 1H), 1.19 (d, *J* 7.0 Hz, 3H); **^{13}C NMR** (101 MHz, $CDCl_3$) δ201.7, 163.4, 139.1, 131.3, 130.8, 130.5, 129.2, 119.9, 113.8, 55.4, 42.1, 38.7, 17.8; **HRMS** (ESI$^+$) calculated for $[C_{17}H_{17}O_2Br+H]^+$ 333.0485, found 333.0477, (Δ -2.5 ppm).

(±)4-Methoxyphenyl 2-methyl-3-phenylpropanoate, 31

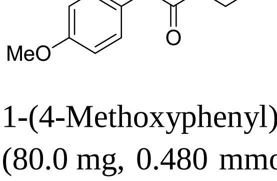

1-(4-Methoxyphenyl)-2-methyl-3-phenylpropan-1-one(**27**, 30.0 mg, 0.120 mmol), mCPBA (80.0 mg, 0.480 mmol), trifluoroacetic acid (19 μL, 0.24 mmol) and CH_2Cl_2, (2.4 mL) were subjected to general procedure D. Purification by FCC (Petrol/Et_2O 20:1) afforded **31** as a colourless oil (29.5 mg, 0.110 mmol, 92 %). **IR ν_{max}** (cm^{-1}) 3063, 3028, 2973, 2935, 2837,

1751, 1606, 1597, 1505, 1455, 1248, 1192, 1137; **^{1}H NMR** (400 MHz, $CDCl_3$) δ 7.27-7.20 (m, 2H), 7.19-7.14 (m, 3H), 6.79-6.72 (m, 4H), 3.70 (s, 3H), 3.04 (dd, *J* 13.3, 7.6 Hz, 1H), 2.90 (sxt, *J* 7.0 Hz, 1H), 2.75 (dd, *J* 13.3, 7.2 Hz, 1H), 1.24 (d, *J* 6.9 Hz, 3H); **^{13}C NMR** (126 MHz, $CDCl_3$) δ 179.9, 157.1, 144.1, 139.0, 129.1, 128.4, 126.5, 122.2, 144.3, 55.5, 41.6, 39.8, 16.9; **HRMS** (ESI^+) calculated for $[C_{17}H_{18}O_3+Na]^+$ 293.1148, found 293.1149.

(±)4-Methoxyphenyl 2-methylhexanoate, 32

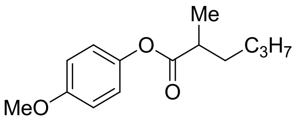

1-(4-Methoxyphenyl)-2-methylhexan-1-one (**22**, 50.0 mg, 0.227 mmol), mCPBA (157 mg, 0.909 mmol), trifluoroacetic acid (35 μL, 0.454 mmol) and CH_2Cl_2, (1.2 mL) were subjected to general procedure D. Purification by FCC (Petrol/Et_2O 10:1) afforded **32** as a colourless oil (46.8 mg, 0.198 mmol, 87%). **IR ν_{max}** (cm^{-1}) 2935, 1754, 1506, 1195, 819, 746; **^{1}H NMR** (400 MHz, $CDCl_3$) δ 6.99 (d, *J* 9.0 Hz, 2H), 6.89 (d, *J* 9.0 Hz, 2H), 3.81 (s, 3H), 2.67 (sxt, *J* 7.0 Hz, 1H), 1.85-1.75 (m, 1H), 1.61-1.50 (m, 1H), 1.48-1.30 (m, 4H), 1.29 (d, *J* 6.9 Hz, 3H), 0.94 (t, *J* 7.1 Hz, 3H); **^{13}C NMR** (126 MHz, $CDCl_3$) δ 175.8, 157.1, 144.4, 122.3, 114.4, 55.6, 39.6, 33.5, 29.4, 22.6, 17.1, 14.0; **HRMS** (ESI^+) calculated for $[C_{14}H_{20}O_3+Na]^+$ 259.1305, found 259.1316, (Δ -4.5 ppm).

(±)4-Methoxyphenyl 2,4-dimethylpentanoate, 33

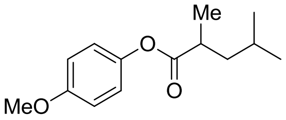

1-(4-Methoxyphenyl)-2,4-dimethylpentan-1-one (**24**, 30.0 mg, 0.135 mmol), mCPBA (93.2 mg, 0.540 mmol), trifluoroacetic acid (21 μL, 0.270 mmol) and CH_2Cl_2, (2.4 mL) were subjected to general procedure D. Purification by FCC (Petrol/Et_2O 10:1) afforded **33** as a colourless oil (28.5 mg, 0.120 mmol, 89%). **IR ν_{max}** (cm^{-1}) 2957, 1752, 1505, 1194, 815, 757; **^{1}H NMR** (400 MHz, $CDCl_3$) δ 6.99 (d, *J* 9.0 Hz, 2H), 6.89 (d, *J* 9.0 Hz, 2H), 3.81 (s, 3H), 2.75 (sxt, *J* 7.1 Hz, 1H), 1.80-1.68 (m, 2H), 1.40-1.34 (m, 1H), 1.28 (d, *J* 7.1 Hz, 3H), 0.98 (d, *J* 6.5Hz, 3H), 0.95 (d, *J* 6.3 Hz, 3H) ; **^{13}C NMR** (126 MHz, $CDCl_3$) δ 176.0, 157.1, 144.3, 122.3, 114.4, 55.6, 43.0, 37.7, 26.0, 22.5, 22.5, 17.5; **HRMS** (ESI^+) calculated for $[C_{14}H_{20}$-$O_3+Na]^+$ 259.1305, found 259.1314, (Δ -3.4 ppm).

(±)4-Methoxyphenyl 3-(4-bromophenyl)-2-methylpropanoate, 34

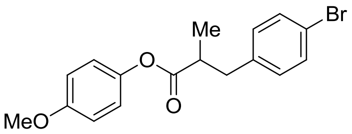

3-(4-Bromophenyl)-1-(4-methoxyphenyl)-2-methylpropan-1-one (**30**, 30.0 mg, 0.0900 mmol), mCPBA (62.1 mg, 0.360 mmol), trifluoroacetic acid (14 μL, 0.180 mmol) and CH_2Cl_2, (2.4 mL) were subjected to general procedure D. Purification by FCC (toluene) afforded **34** as a

yellow oil (27.7 mg, 0.0794 mmol, 88%). **IR ν$_{max}$** (cm^{-1}) 2934, 2836, 1751, 1505, 1489, 1460, 1248, 1192, 1140, 1103, 1011; **^{1}H NMR** (400 MHz, CDCl3) δ 7.45 (d, *J* 8.4 Hz, 2H), 7.13 (d, *J* 8.4 Hz, 2H), 6.90-6.84 (m, 4H), 3.80 (s, 3H), 3.09 (dd, *J* 13.4, 7.7 Hz, 1H), 3.00-2.91 (m, 1H), 2.79 (dd, *J* 13.4, 7.0 Hz, 1H), 1.32 (d, *J* 7.0 Hz, 3H); **^{13}C NMR** (101 MHz, CDCl3) δ 174.6, 157.2 , 144.0, 138.0, 131.5, 130.8, 122.4, 120.3, 114.4, 55.5, 41.4 , 39.1, 17.0; **HRMS** (ESI+) calculated for $[C_{17}H_{17}O_3Br+Na]^+$ 371.0253, found: 371.0249 (Δ −1.07 ppm).

4-Methoxyphenyl 2-benzyloxirane-2-carboxylate, 35

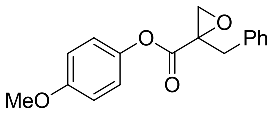

(2-Benzyloxiran-2-yl)(4-methoxyphenyl)methanone (**19**, 26.0 mg, 0.100 mmol), mCPBA (66.0 mg, 0.400 mmol), trifluoroacetic acid (14 µL, 0.20 mmol) and CH_2Cl_2 (2.0 mL) were subjected to general procedure D. Purification by FCC (Petrol/Et_2O 20:1→10:1) afforded **35** (25.0 mg, 0.0880 mmol, 88 %) as a colourless oil. **IR ν$_{max}$** (cm^{-1}) 2925, 1753, 1503, 1248, 1190, 1097; **^{1}H NMR** (400 MHz, $CDCl_3$) δ 7.38-7.24 (m, 5H), 6.91-6.84 (m, 4H), 3.79 (s, 3H), 3.51 (d, *J* 14.9 Hz, 1H), 3.24 (d, *J* 5.8 Hz, 1H), 3.22 (d, *J* 14.9 Hz, 1H), 2.86 (d, *J* 5.8 Hz, 1H); **^{13}C NMR** (126 MHz, $CDCl_3$) δ 169.1, 157.5, 143.7, 135.3, 129.8, 128.4, 127.0, 121.9, 114.5, 57.2, 55.5, 51.2, 36.9; **HRMS** (ESI$^+$) calculated for $[C_{17}H_{16}O_4+Na]^+$ 307.0941, found 307.0932, (Δ −2.8 ppm).

4-Methoxyphenyl 2-isopentyloxirane-2-carboxylate, 36

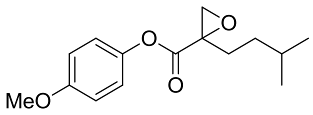

(2-Isopentyloxiran-2-yl)(4-methoxyphenyl)methanone (**18**, 22.0 mg, 0.090 mmol), mCPBA (61.0 mg, 0.360 mmol), trifluoroacetic acid (14 µL, 0.20 mmol) and CH_2Cl_2 (1.8 mL) were subjected to general procedure D. Purification by FCC (Petrol/Et_2O 10:1) afforded **36** (17.5 mg, 0.0660 mmol, 74%) as a colourless oil. **IR ν$_{max}$** (cm^{-1}) 2956, 1753, 1505, 1248, 1192; **^{1}H NMR** (400 MHz, $CDCl_3$) δ 7.02 (d, *J* 9.1 Hz, 2H), 6.90 (d, *J* 9.1 Hz, 2H), 3.81 (s, 3H), 3.23 (d, *J* 5.8 Hz, 1H), 2.91 (d, *J* 5.8 Hz, 1H), 2.19 (ddd, *J* 5.0, 11.6, 14.2 Hz, 1H), 1.79 (ddd, *J* 5.2, 11.4, 14.2 Hz, 1H), 1.67-1.56 (m, 1H), 1.53-1.34 (m, 2H), 0.93 (d, *J* 6.7 Hz, 6H); **^{13}C NMR** (126 MHz, $CDCl_3$) δ 169.5, 157.4, 143.8, 122.0, 114.5, 57.3, 55.6, 52.0, 33.6, 29.2, 28.0, 22.4, 22.3; **m/z** (ESI+) 287.1 **HRMS** (ESI$^+$) calculated for $[C_{15}H_{20}O_4+Na]^+$ 287.1254 , found 287.1255 (Δ −1.9 ppm).

3,5-Dibenzyl-2-cyclopropyl-6-(4-methoxyphenyl)pyridine, 37

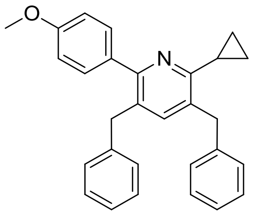

(±)(2*RS*,4*SS*)-2,4-Dibenzyl-1-cyclopropyl-5-(4-methoxyphenyl)pentane-1,5-dione (**16**, 30 mg, 0.0703 mmol), ammonium acetate (16.3 mg, 0.211 mmol), copper (II) acetate monohydrate (35.1 mg, 0.176 mmol) were subjected to general procedure F for 24 h. Purification by FCC (toluene/Et_2O 20:1) afforded **37** as a yellow oil (27.0 mg, 0.0667 mmol, 95%). ν_{max} (thin film)/cm^{-1} 3002, 1608, 1513, 1449, 1249, 1175, 840, 727, 698; ^{1}H NMR (500MHz , $CDCl_3$) δ 7.36 - 7.30 (m, 2 H), 7.22 - 7.04 (m, 9 H), 6.92 (dd, *J* = 0.9, 7.8 Hz, 2 H), 6.85 - 6.79 (m, 2 H), 4.03 (s, 2 H), 3.91 (s, 2 H), 3.74 (s, 3 H), 2.04 - 1.91 (m, 1 H), 1.08 - 0.97 (m, 2 H), 0.82 - 0.68 (m, 2 H); ^{13}C NMR (126MHz , $CDCl_3$) δ 159.3, 157.9, 155.5, 141.1, 140.1, 140.0, 133.4, 131.7, 130.5, 129.5, 128.7, 128.7, 128.5, 128.5, 126.2, 126.0, 113.4, 55.3, 38.2, 37.9, 13.7, 9.0; HRMS (ESI$^+$) calculated for $[C_{29}H_{27}NO+H]^+$ 406.2165, found 406.2162 (Δ –0.72 ppm).

3,5-Dibenzyl-2-(4-methoxyphenyl)-6-phenylpyridine, 38

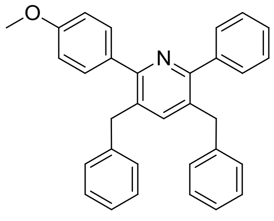

(±)(2*SS*,4*RS*)-2,4-Dibenzyl-1-(4-methoxyphenyl)-5-phenylpentane-1,5-dione (**17**, 30 mg, 0.0649 mmol), hydroxylamine hydrochloride (13.6 mg, 0.0974 mmol) were subjected to general procedure E. Purification by FCC (Petrol/Et2O 8:2) afforded **38** as a colorless oil (26.9 mg, 0.0609 mmol, 94%). ν_{max} (thin film)/cm^{-1} 3060, 3026, 2932, 2836, 1609, 1434, 1248, 1175, 728, 699; ^{1}H NMR (500MHz , $CDCl_3$) δ 7.42 (d, *J* 7.6 Hz, 2 H), 7.38 (d, *J* 7.9 Hz, 2 H), 7.33 - 7.24 (m, 4 H), 7.15 (q, *J* 8.0 Hz, 4 H), 7.12 - 7.08 (m, 2 H), 6.93 (d, *J* 7.6 Hz, 2 H), 6.90 (d, *J* 7.4 Hz, 2 H), 6.83 (d, *J* 8.4 Hz, 2 H), 3.96 (s, 2 H), 3.93 (s, 2 H), 3.74 (s, 3 H); ^{13}C NMR (126MHz , $CDCl_3$) δ 159.5, 156.5, 156.3, 141.2, 140.6, 140.2, 132.7, 140.5, 132.2, 132.0, 130.6, 129.3, 128.8, 128.8, 128.5, 128.5, 128.1, 127.9, 126.2, 126.2, 113.6, 55.4, 38.3, 38.2; HRMS (ESI$^+$) calculated for $[C_{32}H_{27}NO+H]^+$ 442.2165, found 442.2157 (Δ –1.97ppm).

3,5-Dibenzyl-2,6-bis(4-methoxyphenyl)pyridine, 39

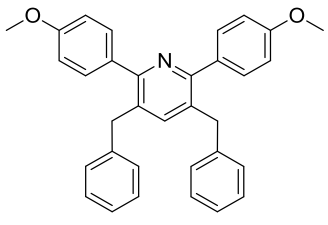

(±)(2*RS*,4*SS*)-2,4-Dibenzyl-1,5-bis(4-methoxyphenyl)pentane-1,5-dione (**5**, 30 mg, 0.0609 mmol), hydroxylamine hydrochloride (12.7 mg, 0.183 mmol) were subjected to general procedure E. Purification by FCC (toluene/Et2O 15:1) afforded **39** as an oily solid (23.2 mg,

0.0492 mmol, 81%). ν_{max} (thin film)/cm-1 3015, 2970, 1609, 1511, 1434, 1294, 1175, 1031, 839; ^{1}H NMR (400 MHz, $CDCl_3$) δ 7.48 (d, *J* 8.6 Hz, 4H), 7.34 (s, 1H), 7.25 (t, *J* 7.7 Hz, 4H), 7.18 (t, *J* 6.8 Hz, 2H), 7.02 (d, *J* 7.6, 4H), 6.93 (d, *J* 8.6, 4H), 4.04 (s, 4H), 3.83 (s, 6H); ^{13}C NMR (101 MHz, $CDCl_3$) δ 159.6, 156.3, 141.4, 140.8, 132.9, 131.9, 130.7, 128.8, 128.6, 126.2, 113.6, 55.4, 38.4; HRMS (ESI^{+}) Calculated for $[C_{33}H_{29}NO_2+H]^+$ 472.2271, found 472.2264 (Δ –1.6 ppm).

IX. Spectral Data

(±)1-(4-Methoxyphenyl)-2-methyl-3-phenylpropan-1-one, 2

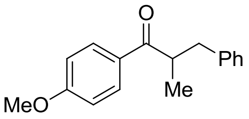

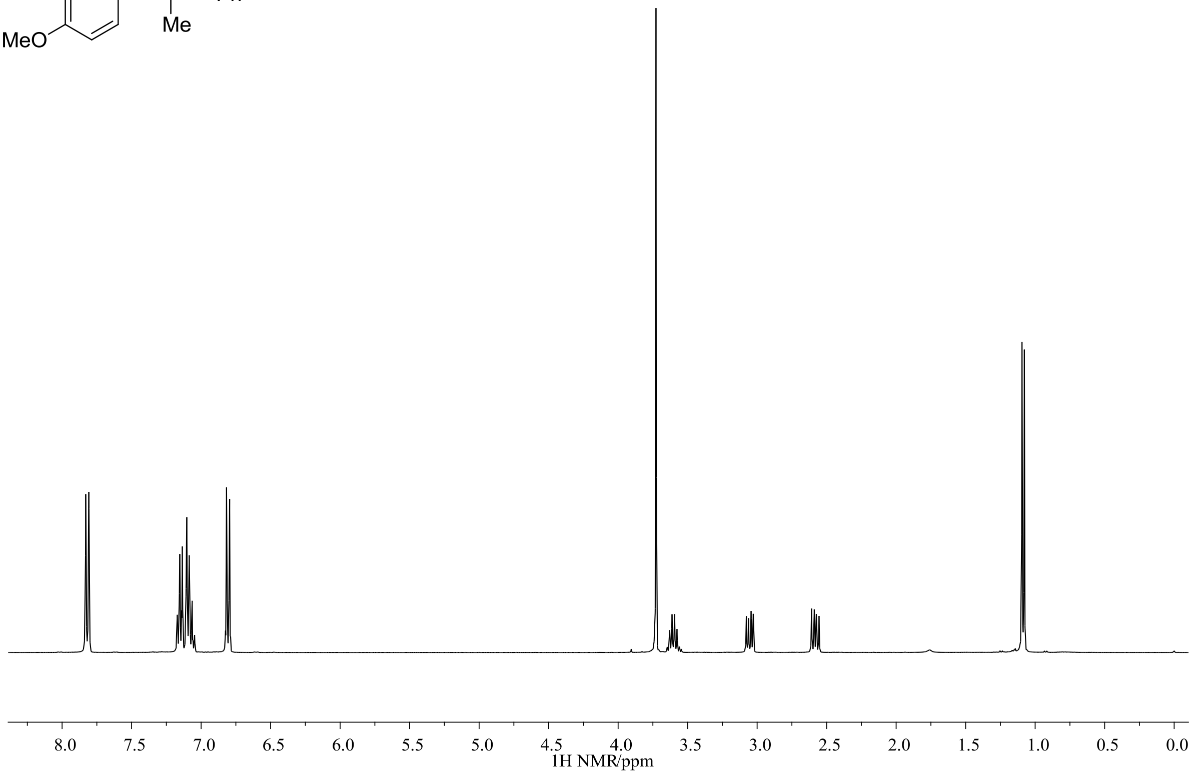

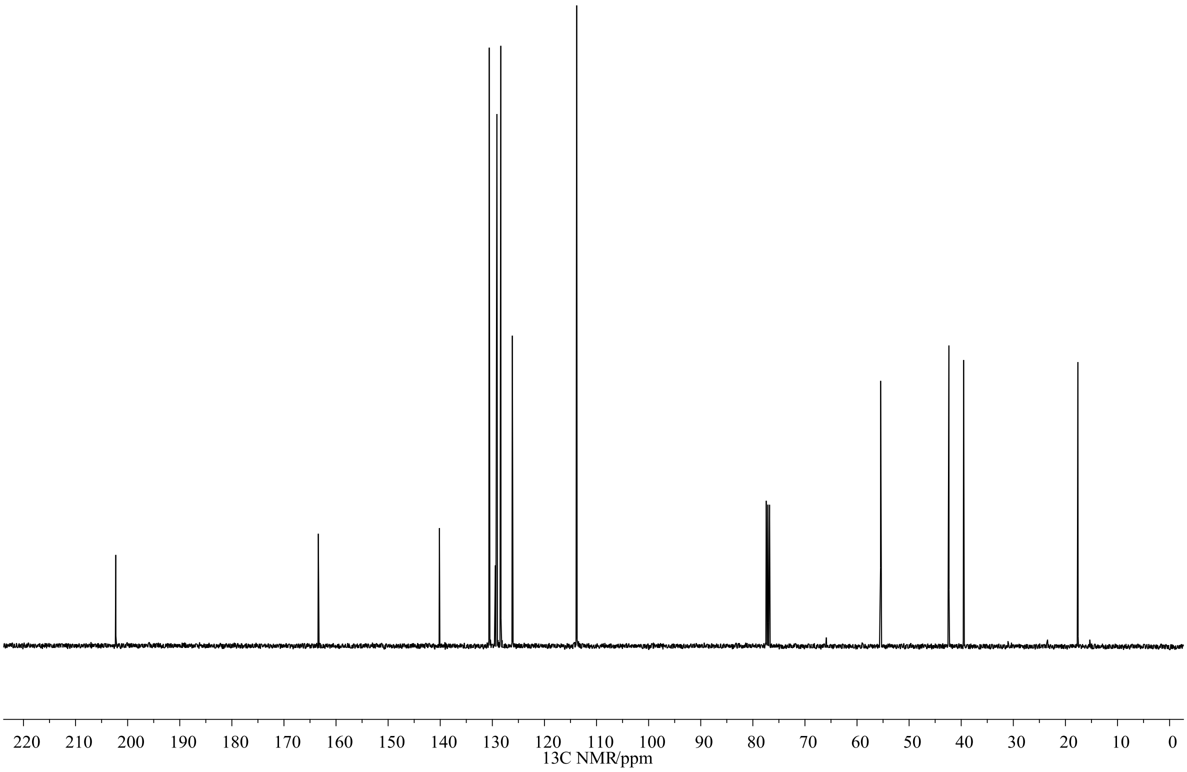

(±)2-Benzyl-3-methoxy-1-(4-methoxyphenyl)propan-1-one, 4

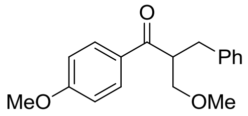

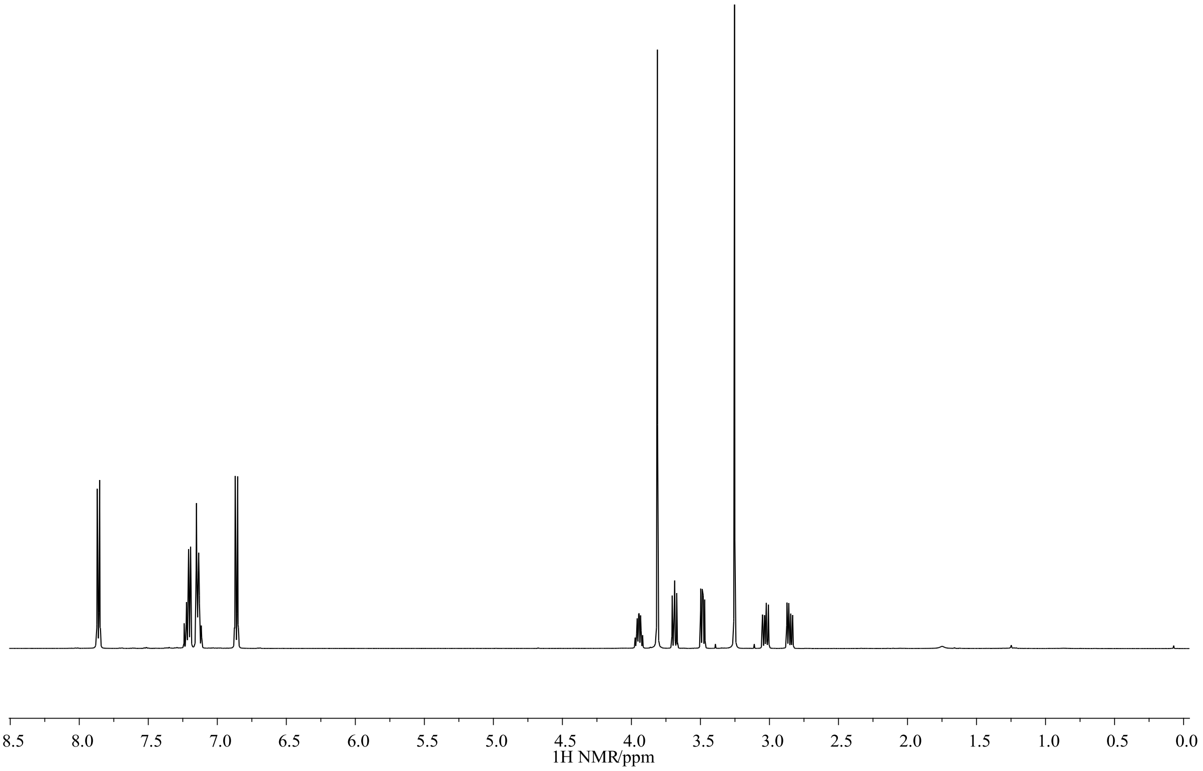

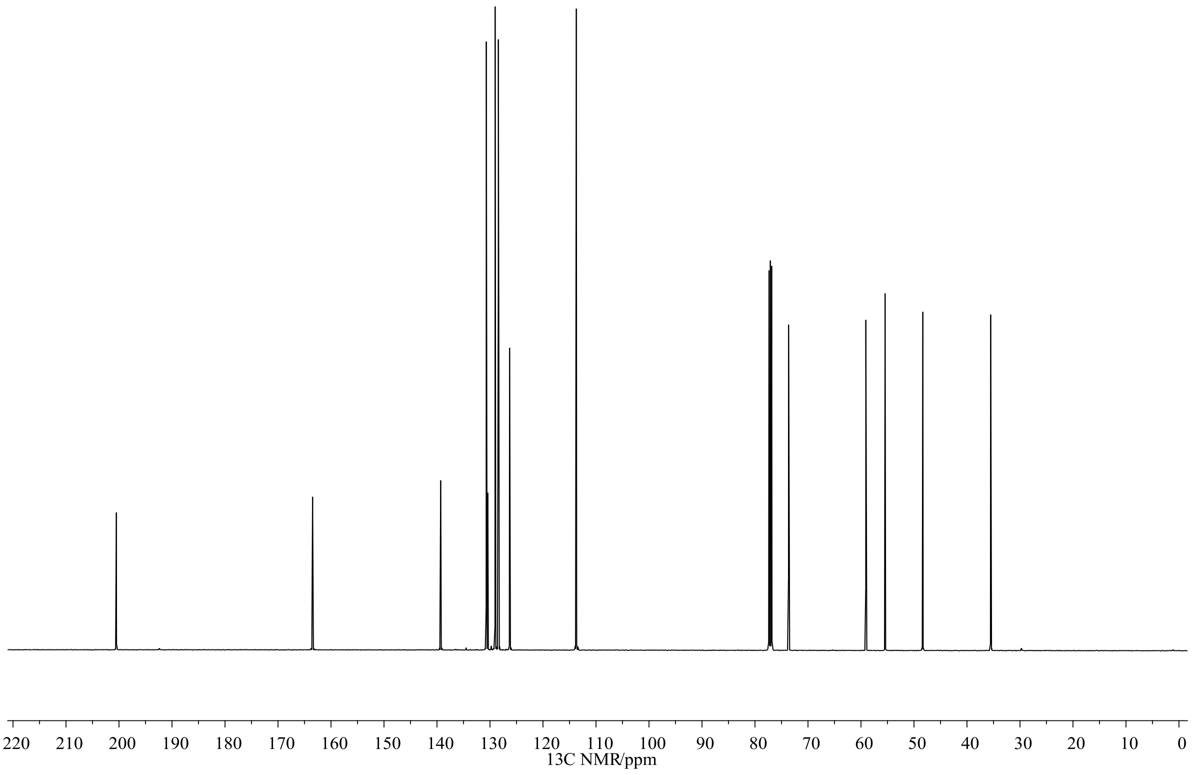

(±)-(2RR, 4RS)-2,4-dibenzyl-1,5-bis(4-methoxyphenyl)pentane-1,5-dione, 5

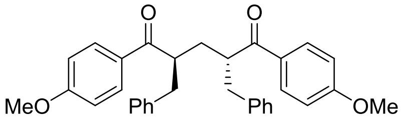

anti-**5**

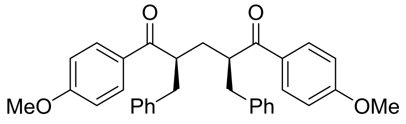

syn-**5**

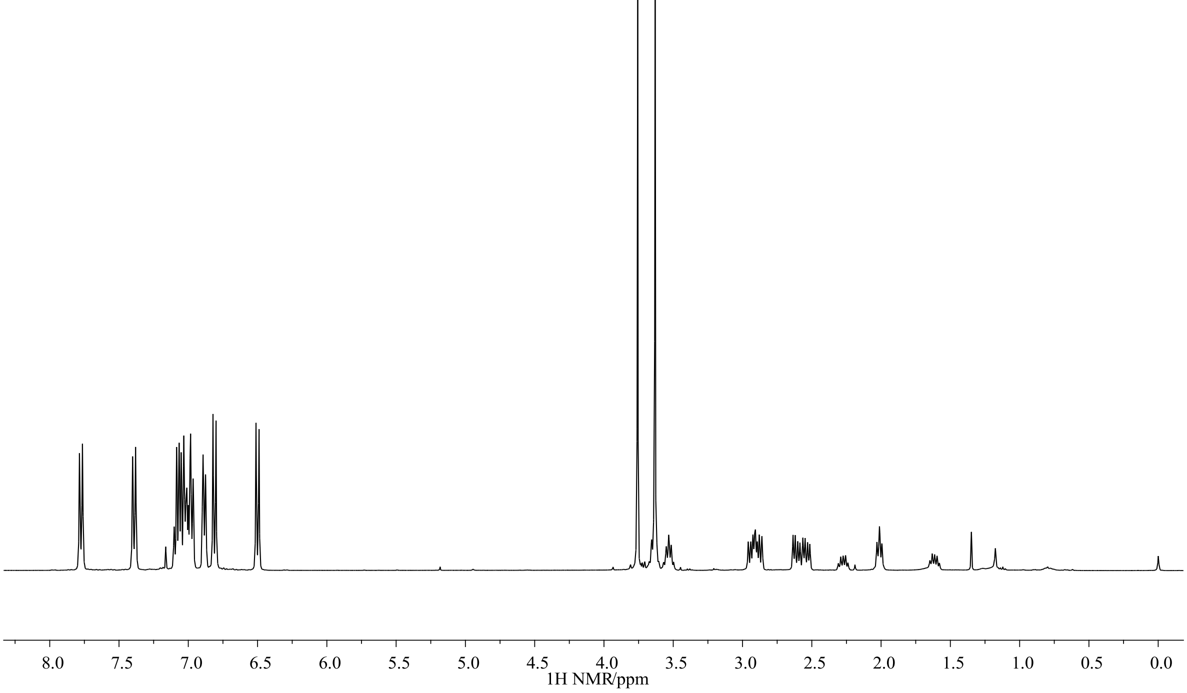

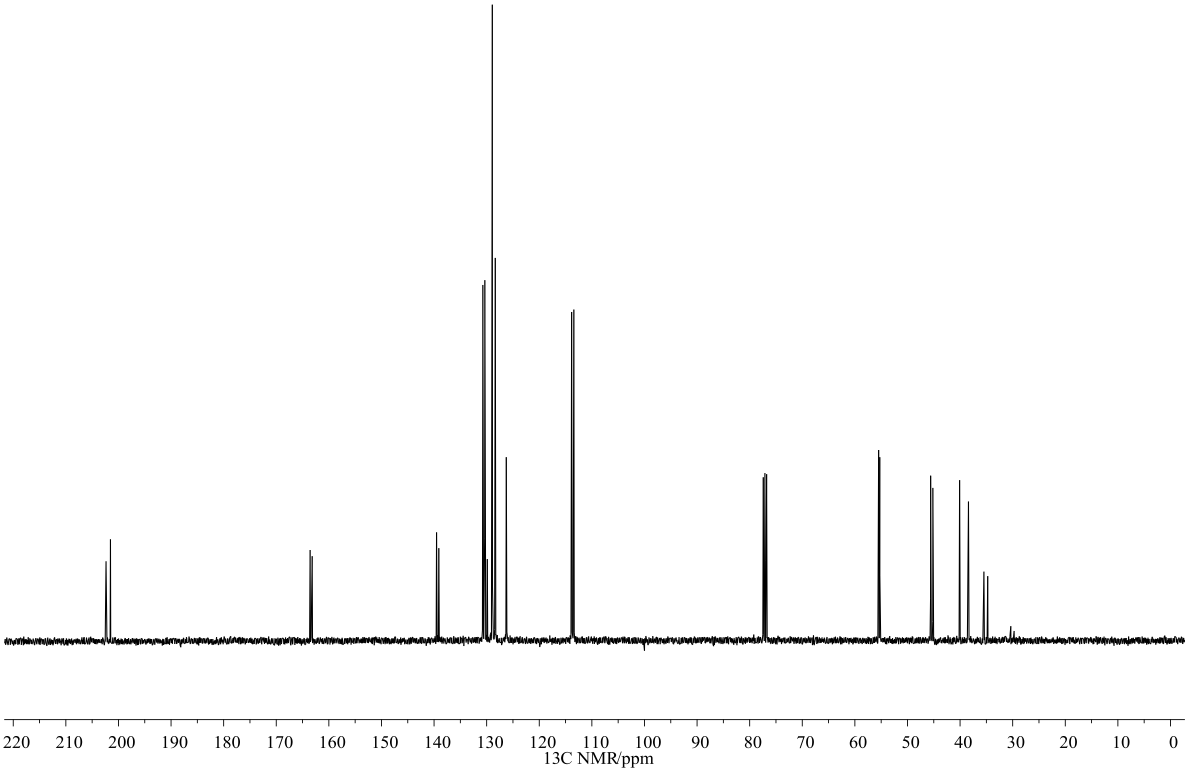

(±)2-(Methoxymethyl)-1-(4-methoxyphenyl)hexan-1-one, 7

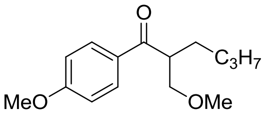

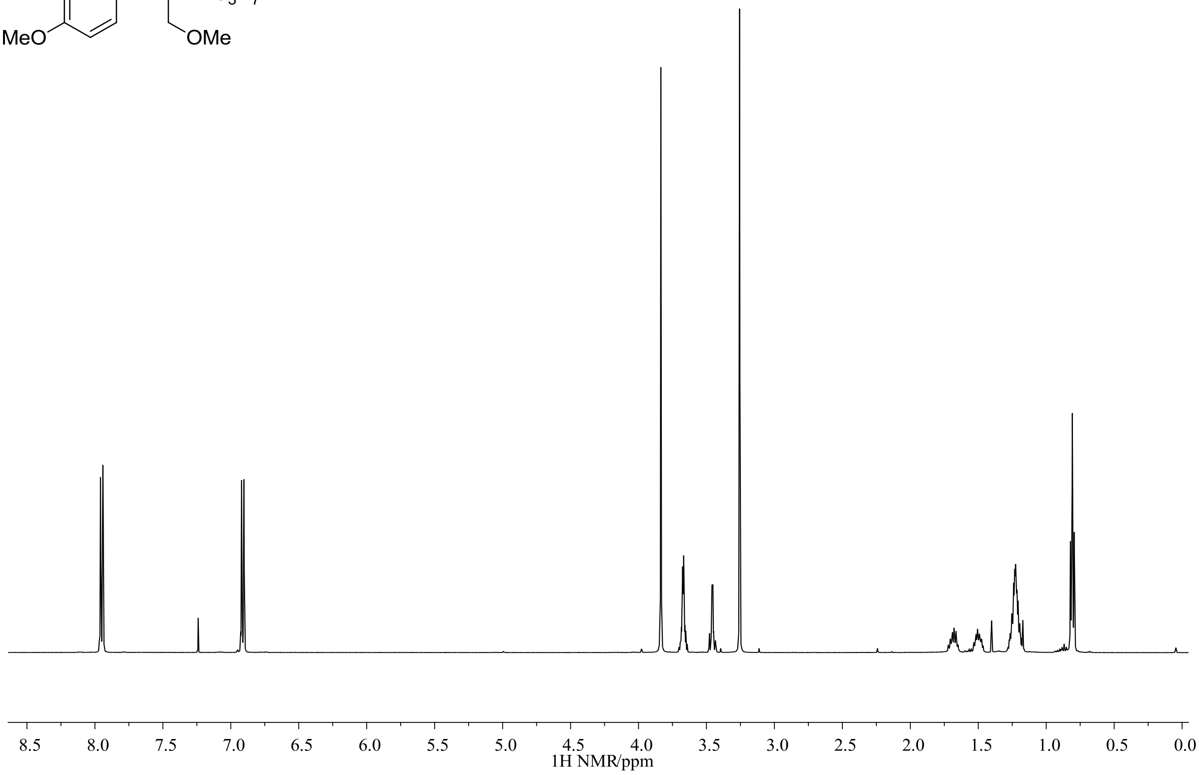

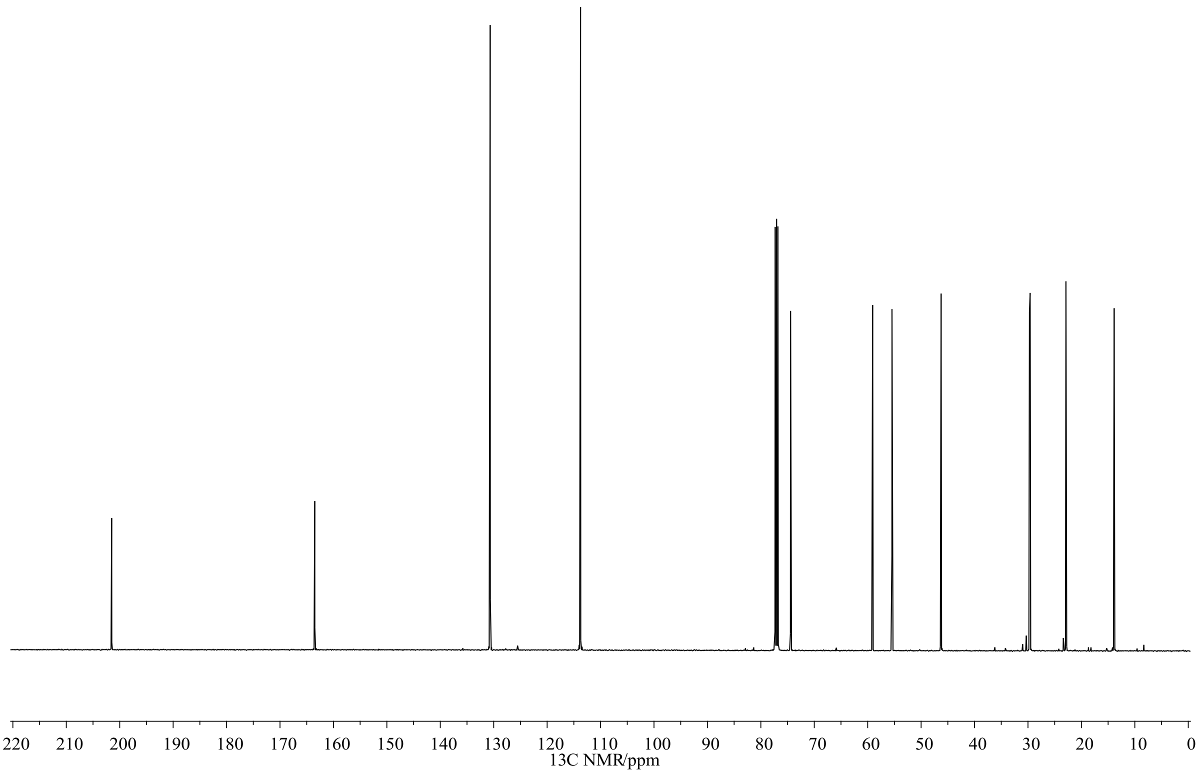

(±)2-(Methoxymethyl)-1-(4-methoxyphenyl)-4-methylpentan-1-one, 9

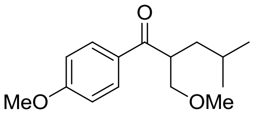

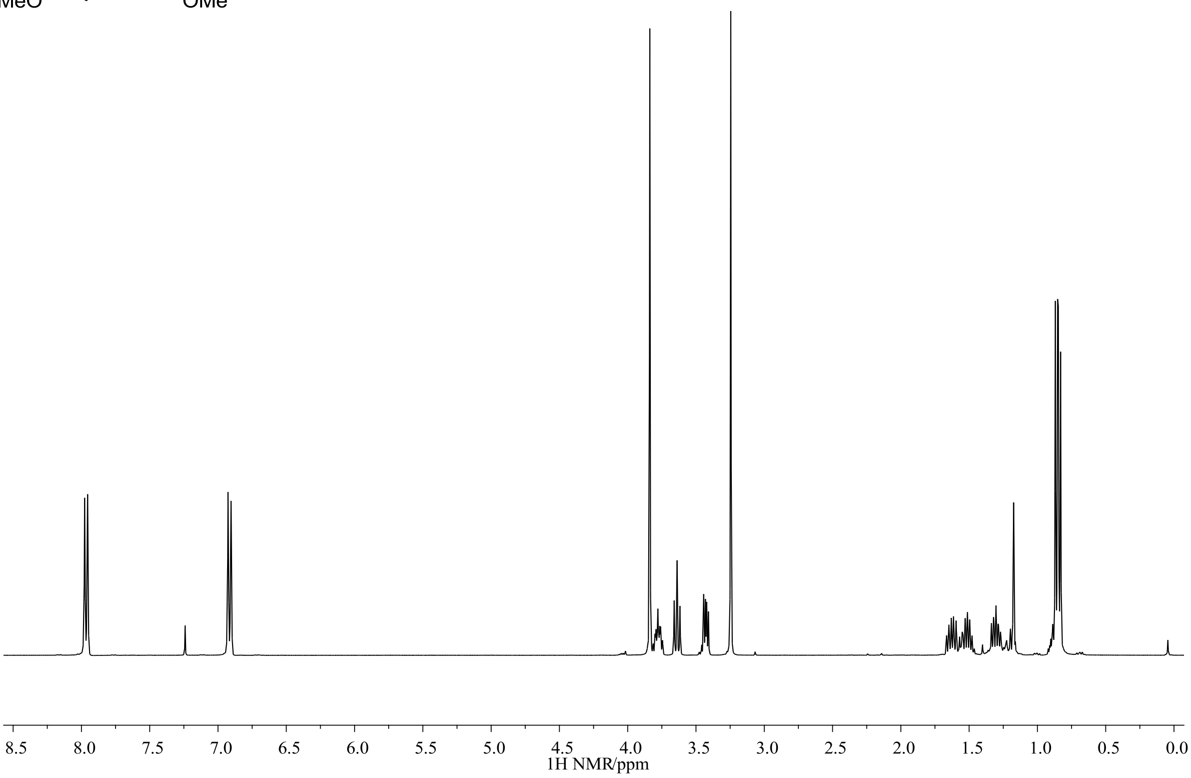

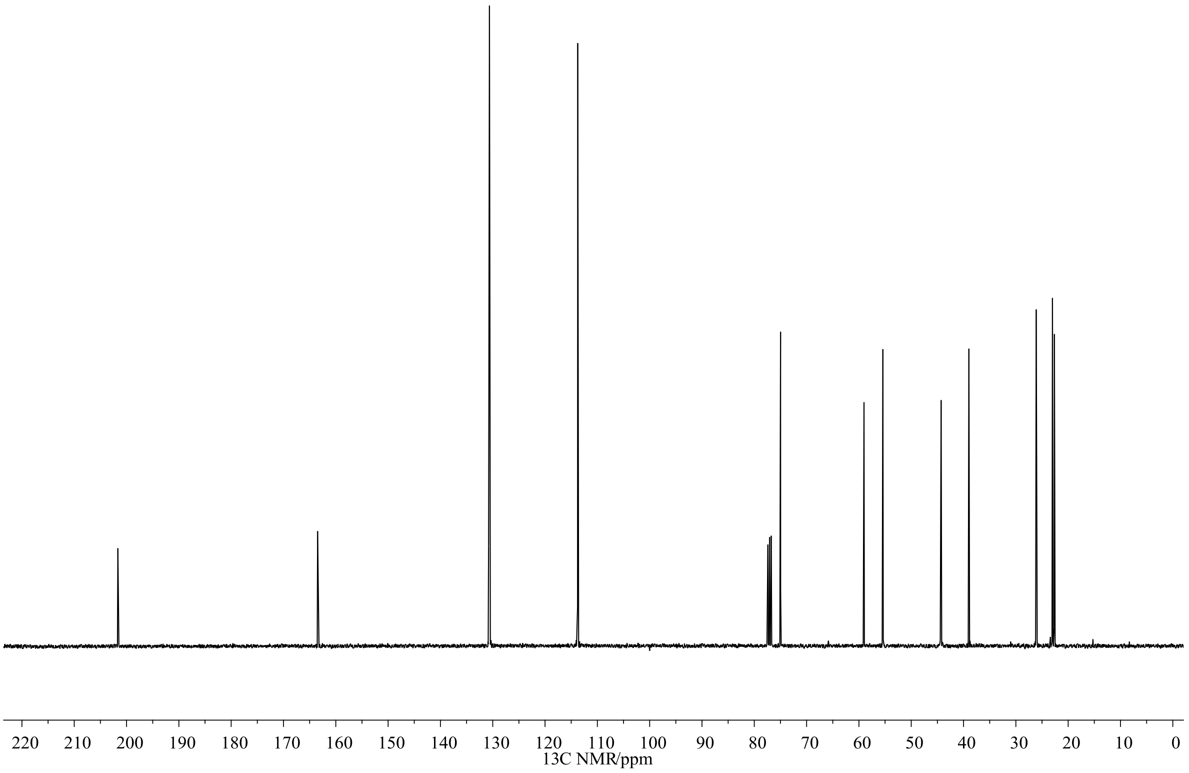

(±)2-(Methoxymethyl)-1-(4-methoxyphenyl)-5-methylhexan-1-one, 11

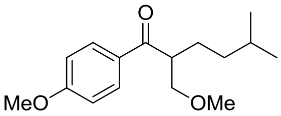

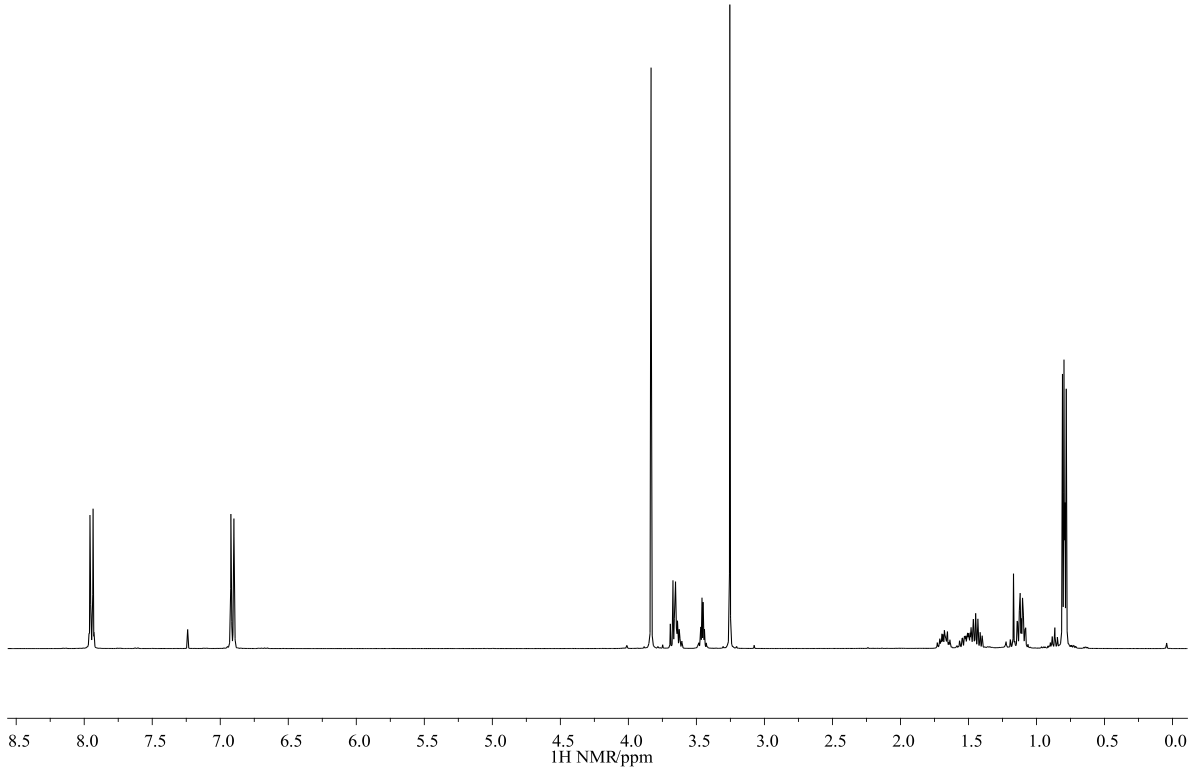

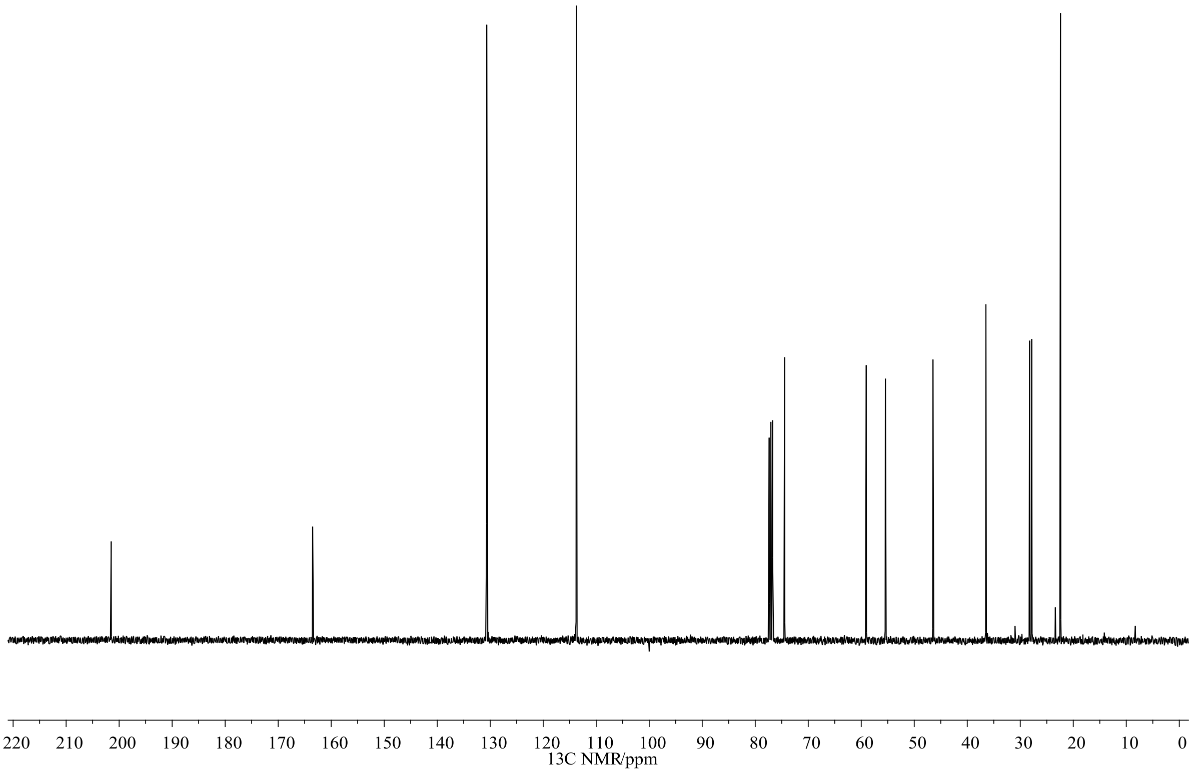

(±)3-Methoxy-2-(4-methoxybenzyl)-1-(4-methoxyphenyl)propan-1-one, 13

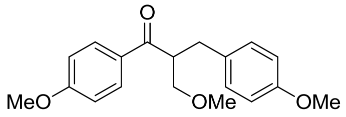

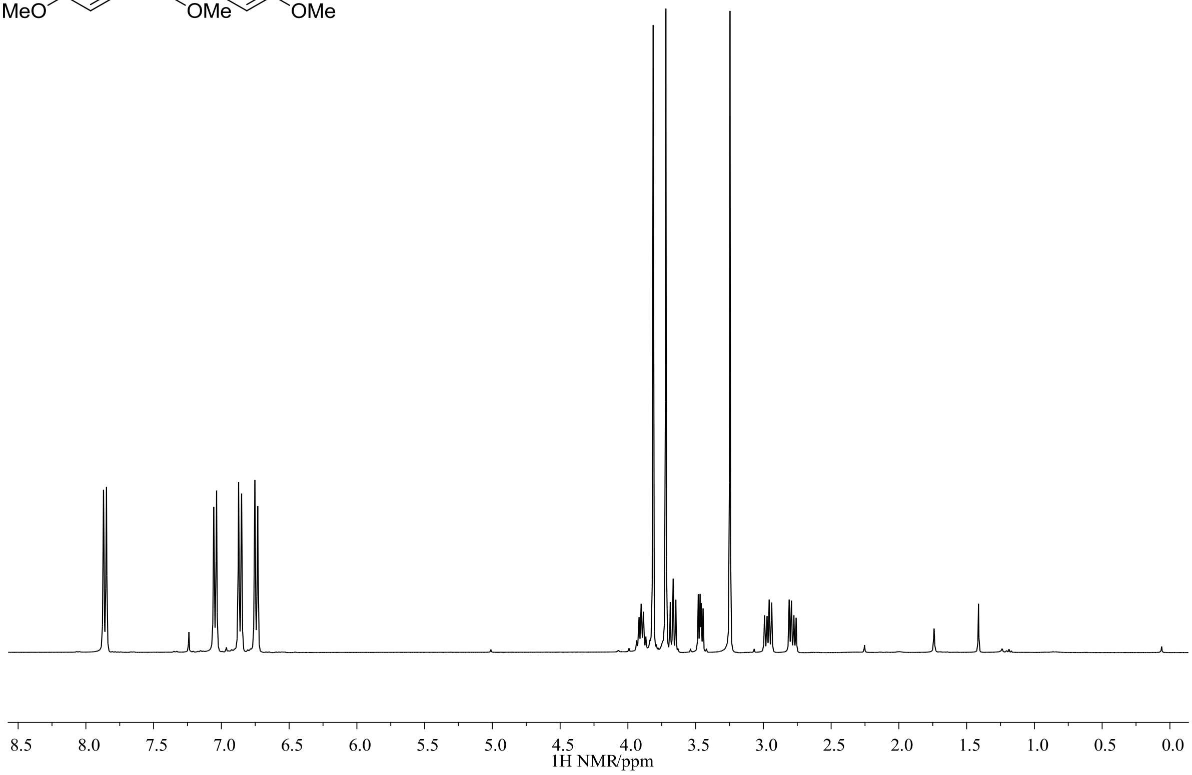

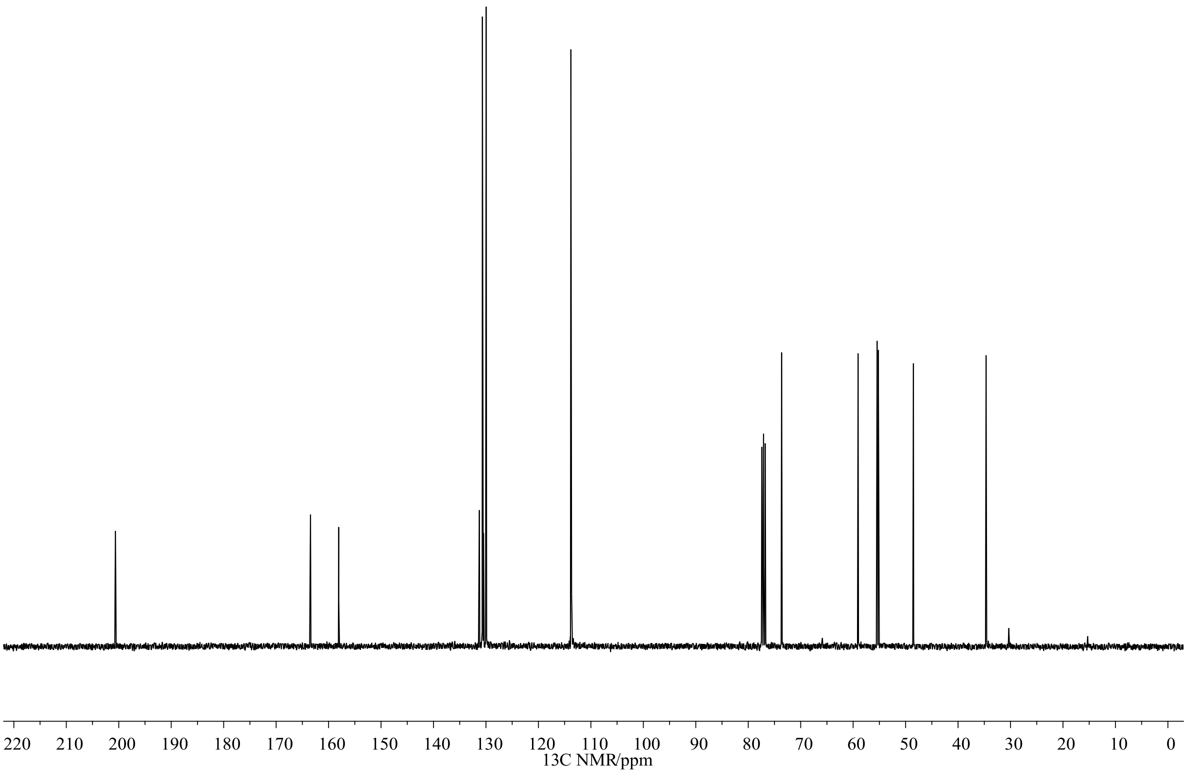

(±)1-(4-Methoxyphenyl)-5-methyl-2-(2-methyl-2-nitropropyl)hexan-1-one, 14

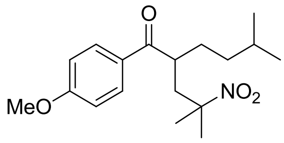

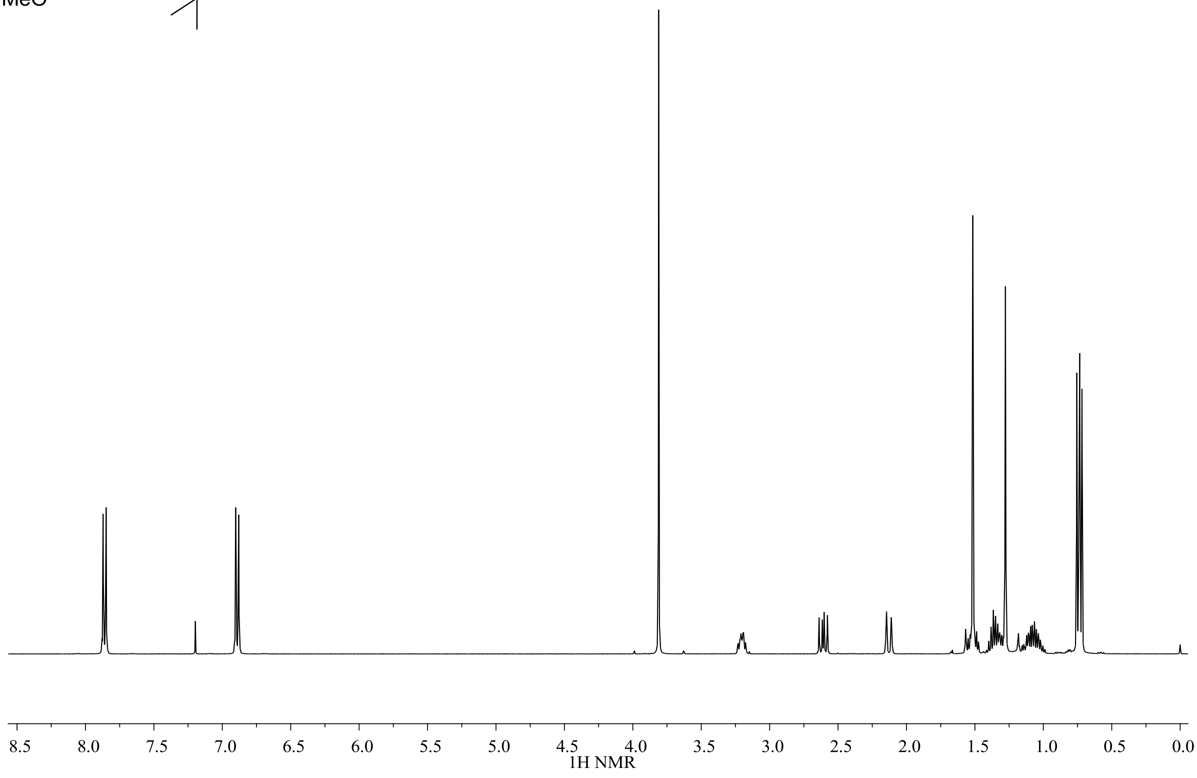

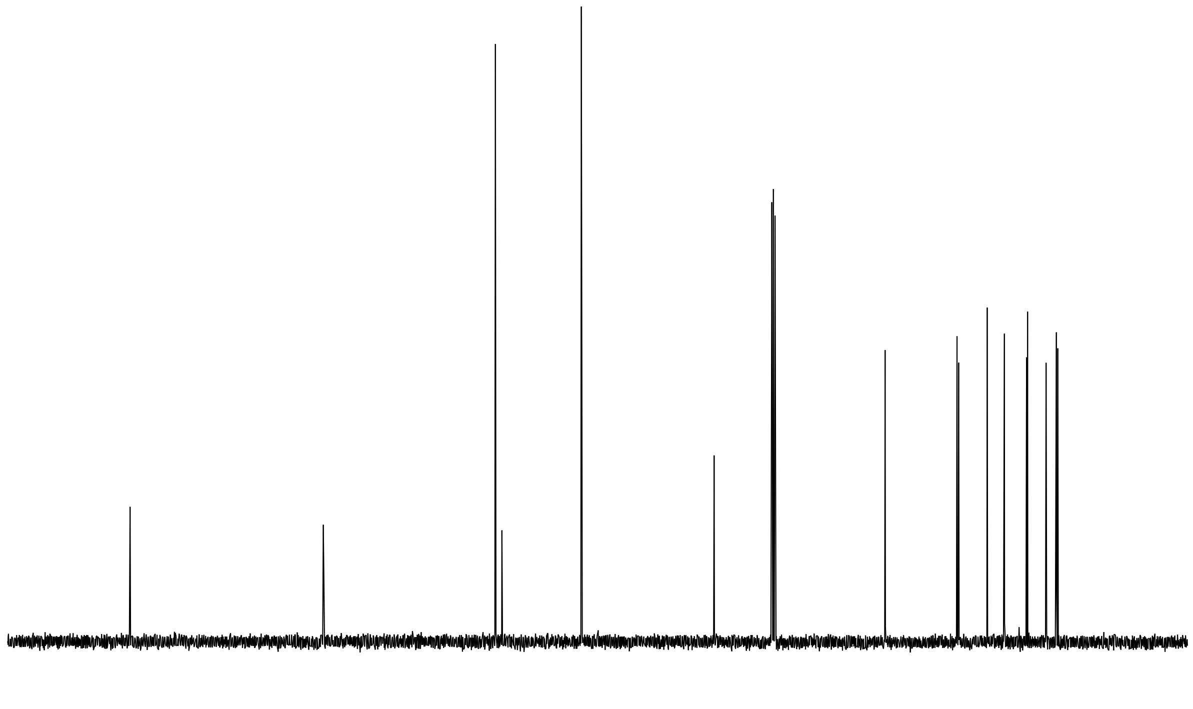

(±)2-Benzyl-1-(4-methoxyphenyl)-4-methyl-4-nitropentan-1-one, 15

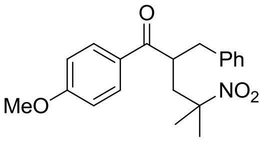

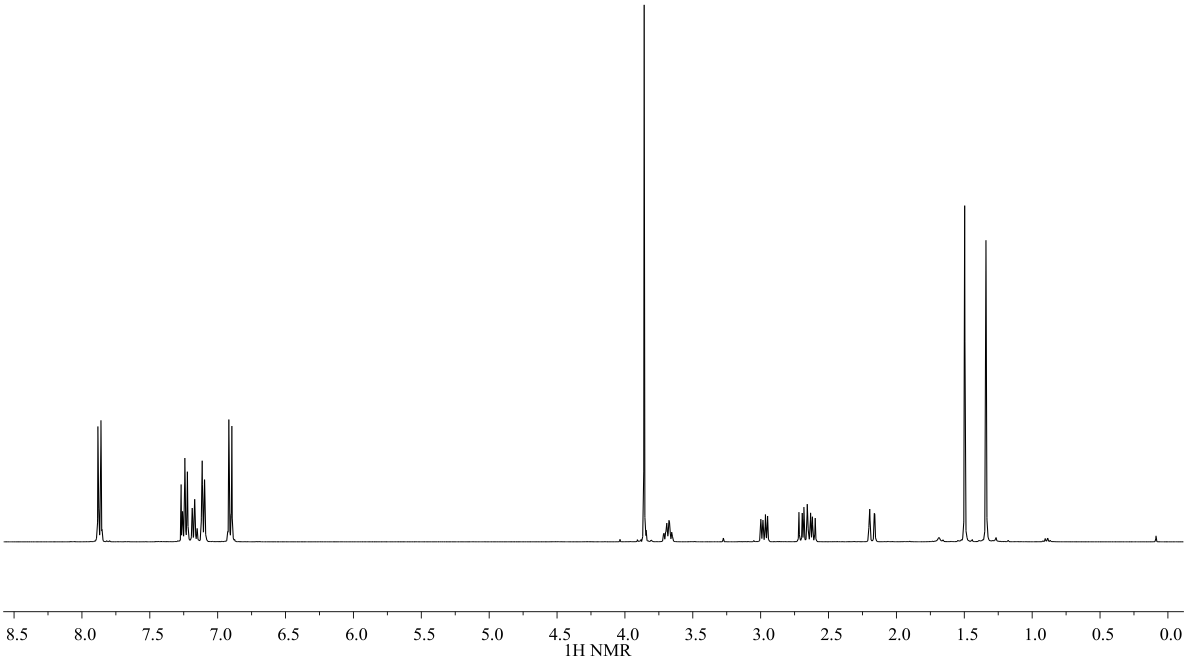

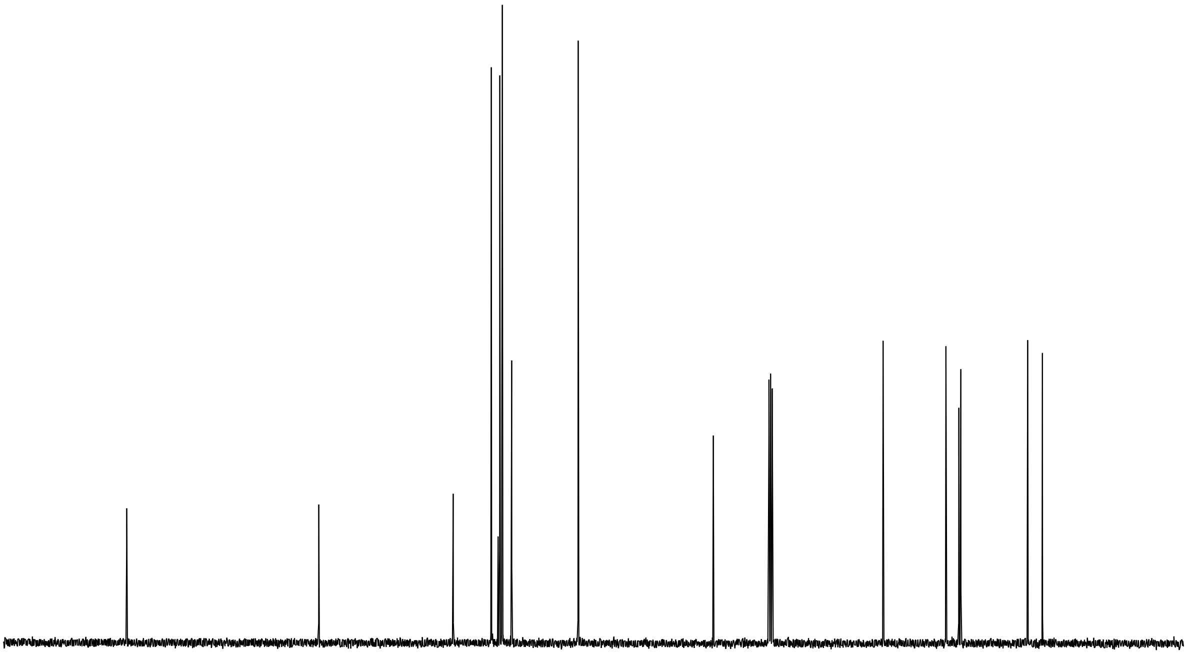

(±)(2*RS*,4*SS*)-2,4-Dibenzyl-1-cyclopropyl-5-(4-methoxyphenyl)pentane-1,5-dione, 16

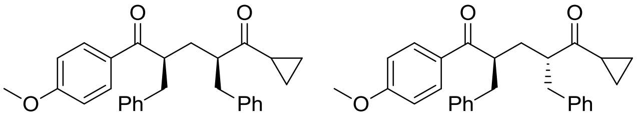

Syn-XX *Anti*-XX

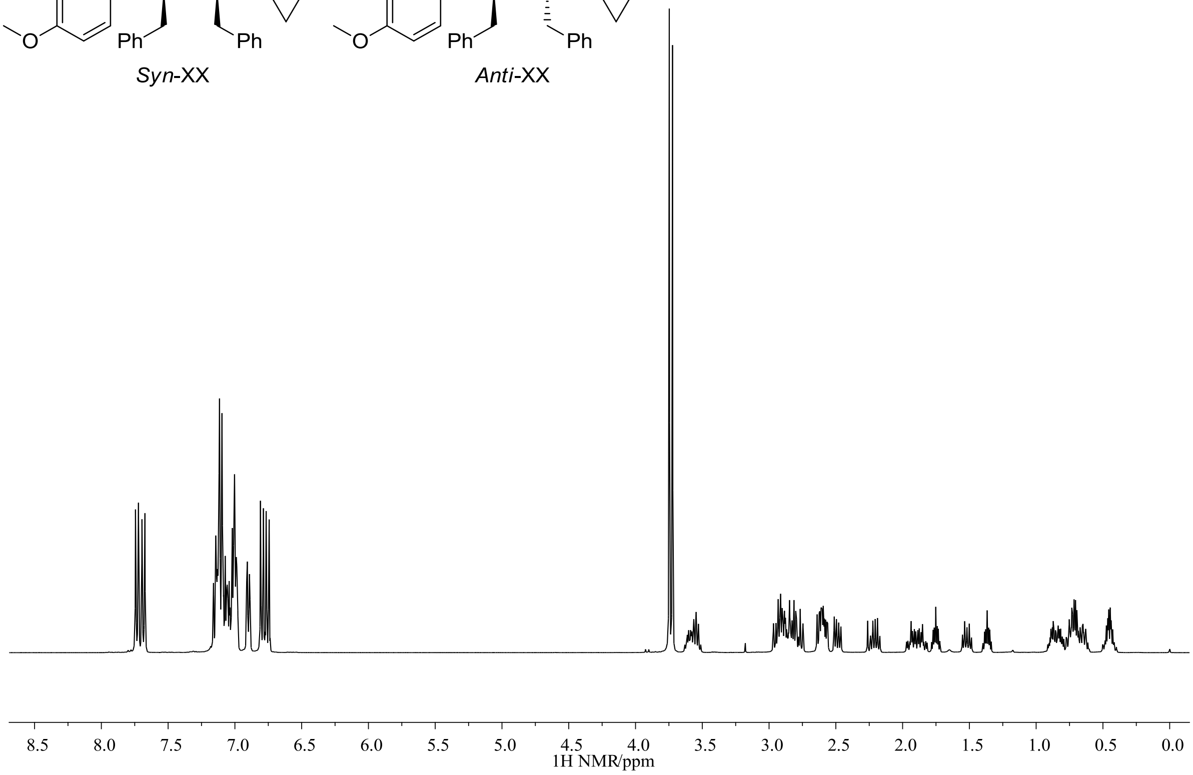

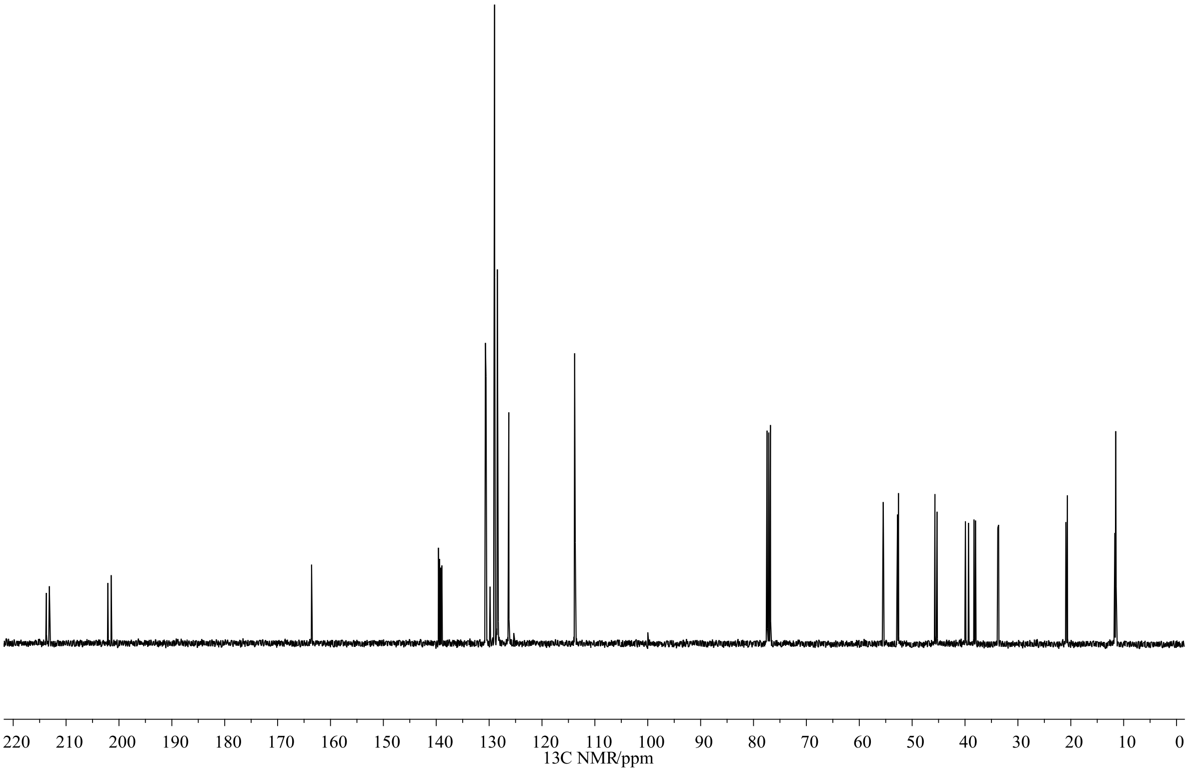

(±) (2SS,4RS)-2,4-Dibenzyl-1-(4-methoxyphenyl)-5-phenylpentane-1,5-dione, 17

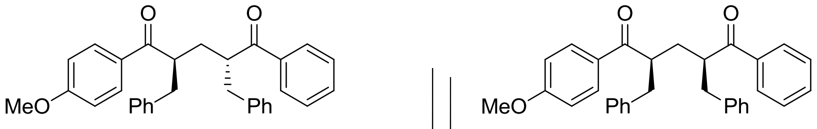

anti-XX *syn*-XX

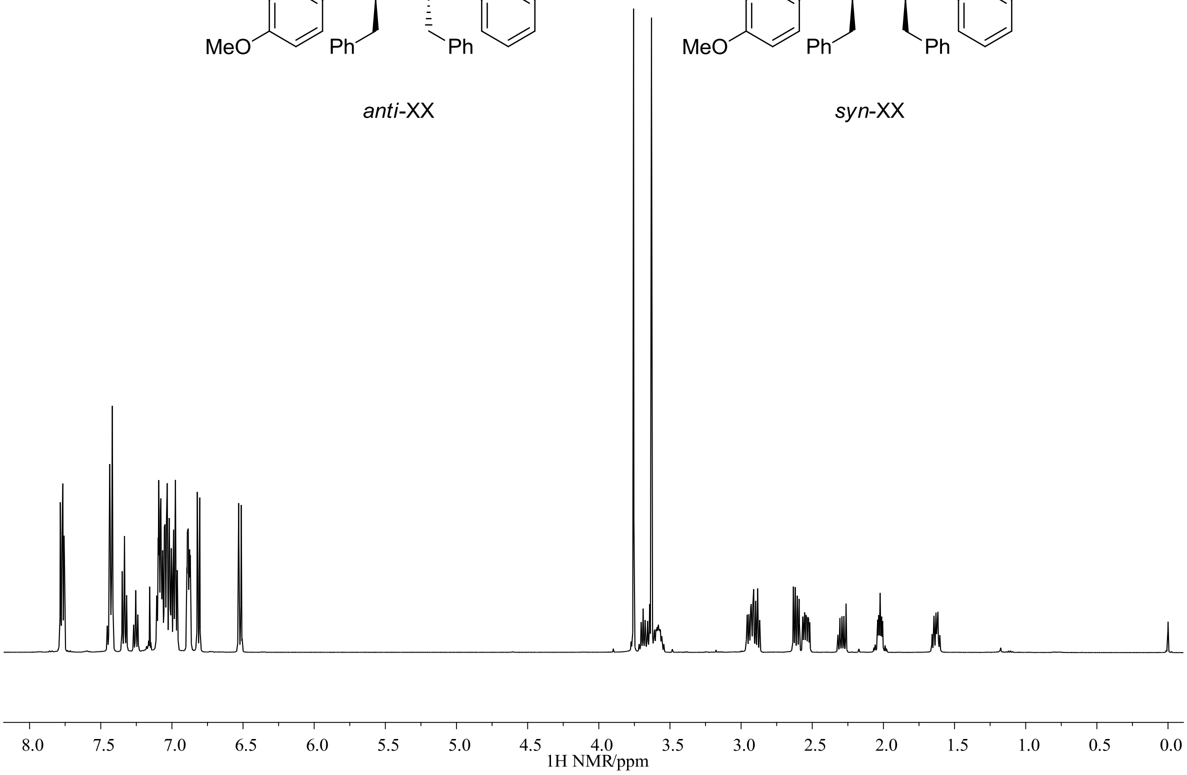

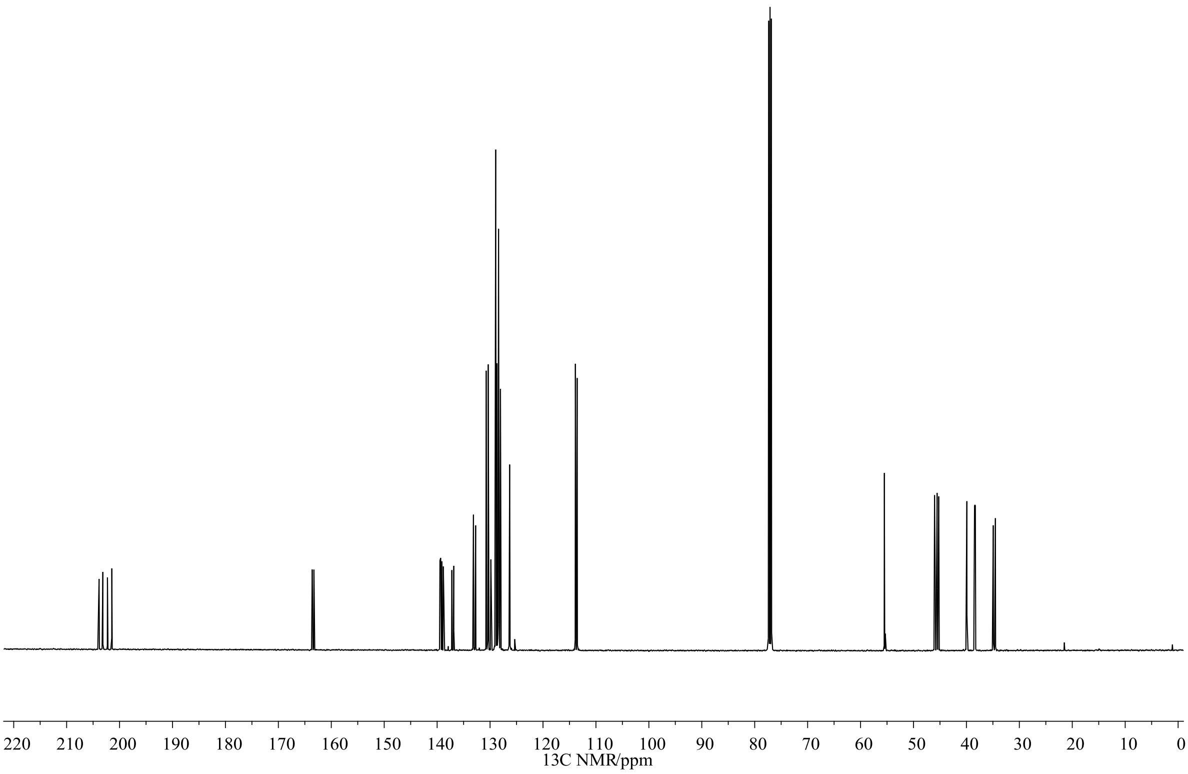

(±) (2-Benzyloxiran-2-yl)(4-methoxyphenyl)methanone, 18

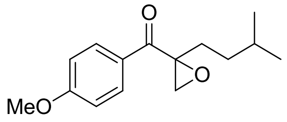

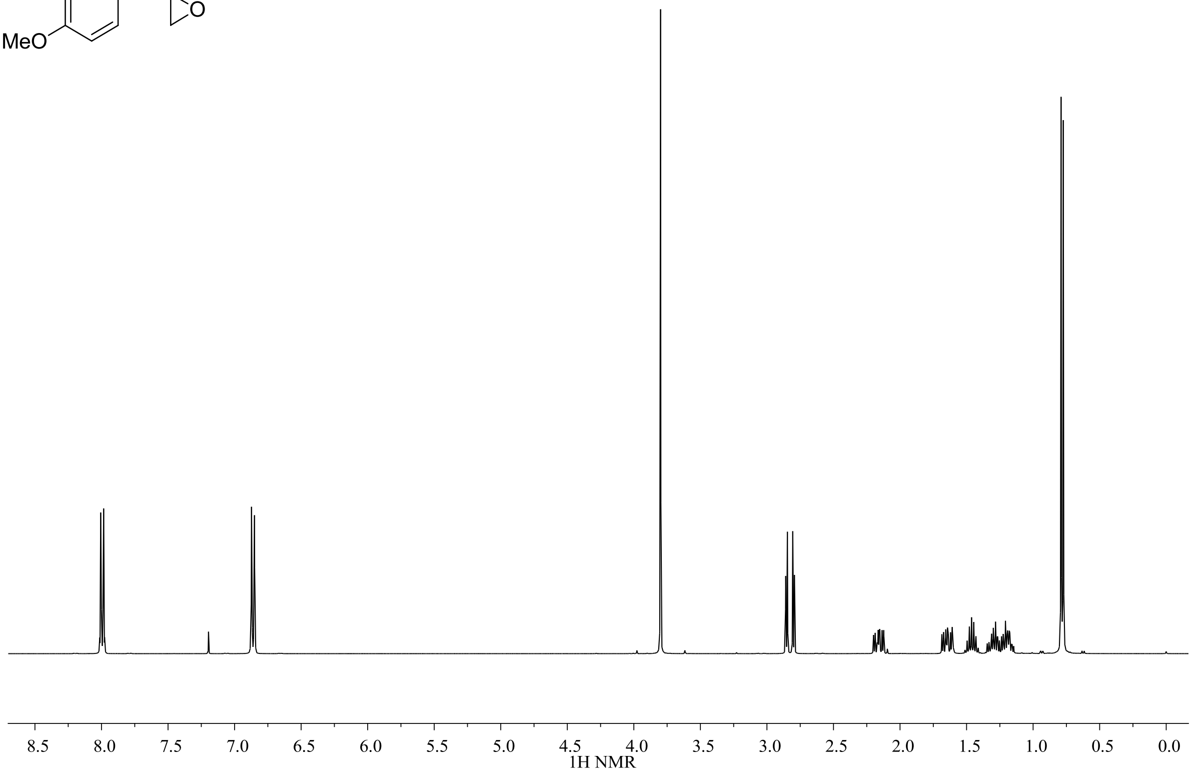

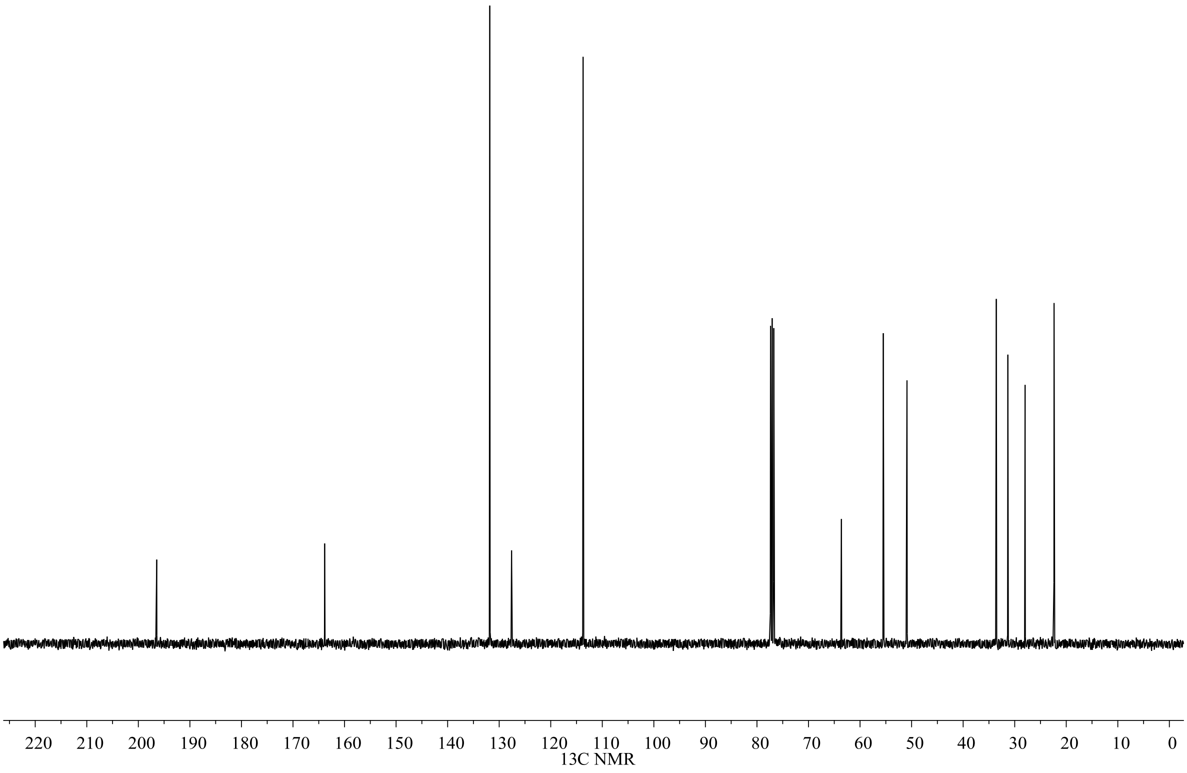

(±) (2-Benzyloxiran-2-yl)(4-methoxyphenyl)methanone, 19

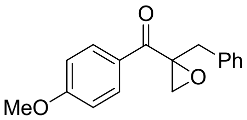

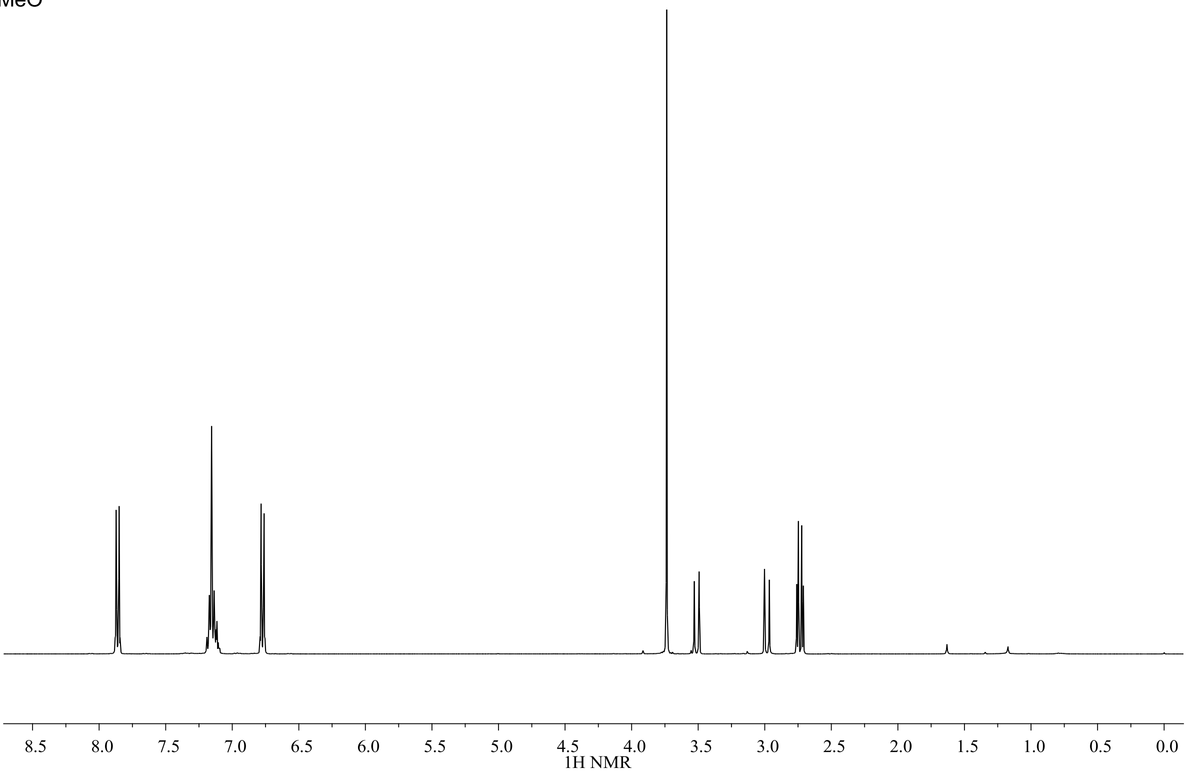

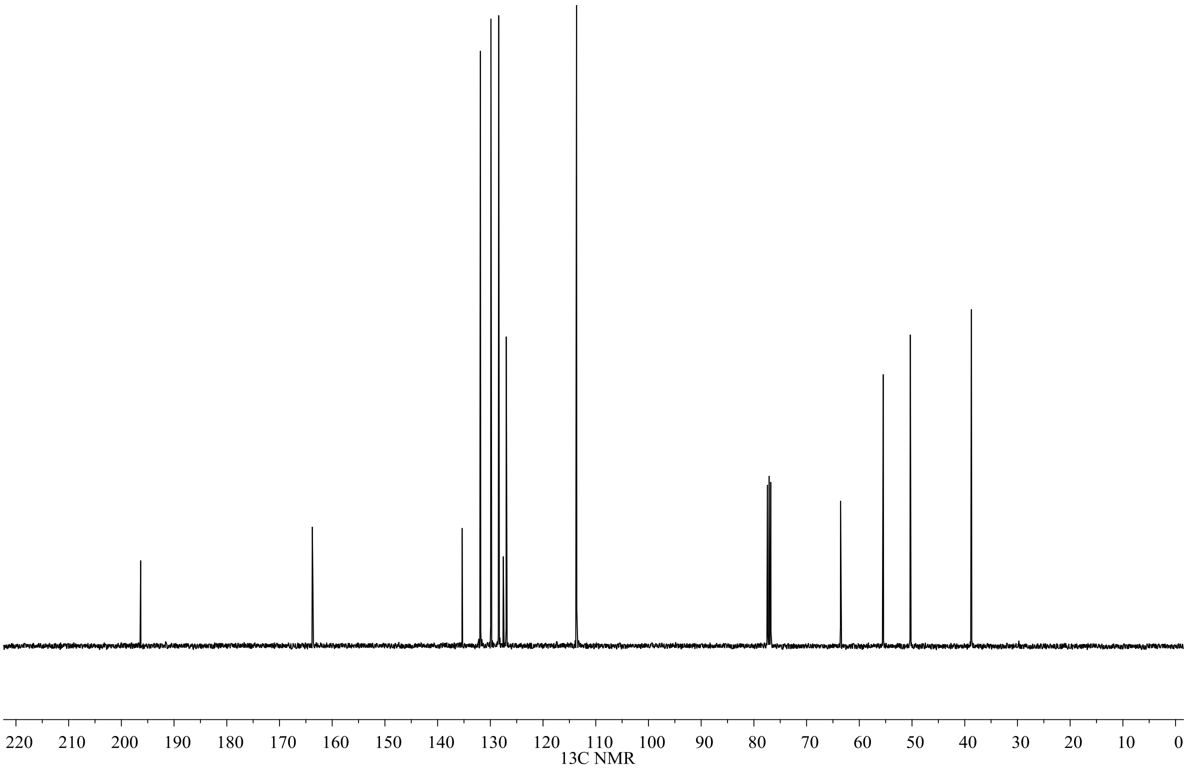

2-Benzyl-1-(4-methoxyphenyl)-3-phenylpropan-1-one, 20

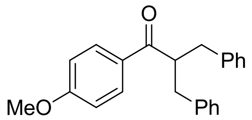

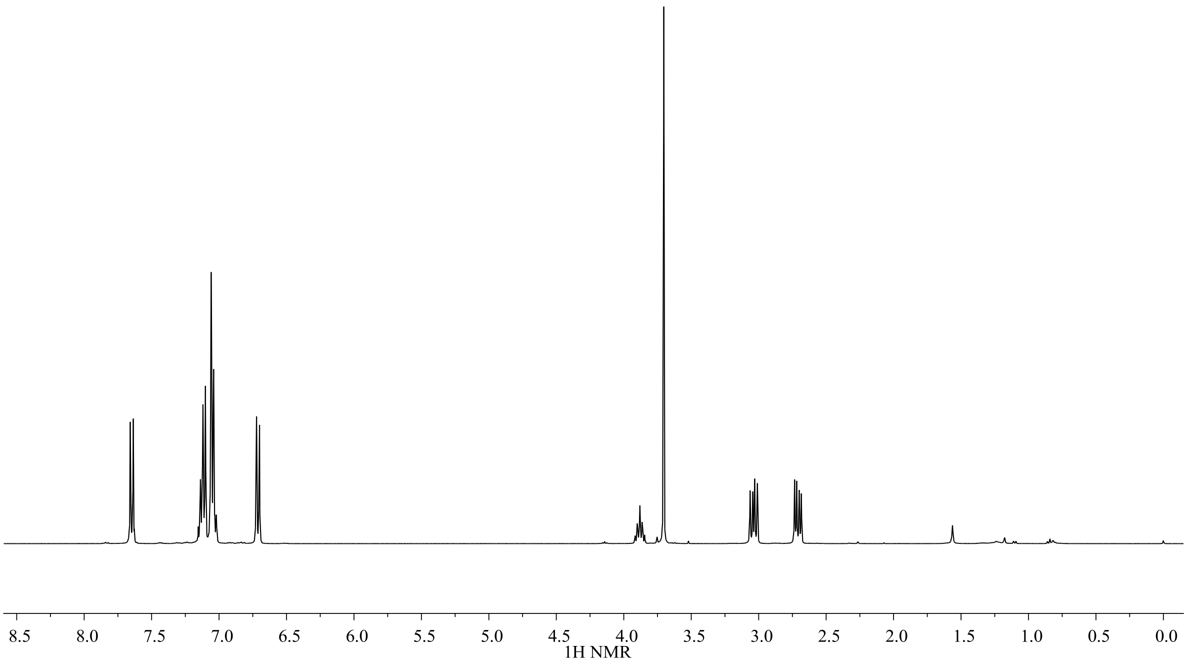

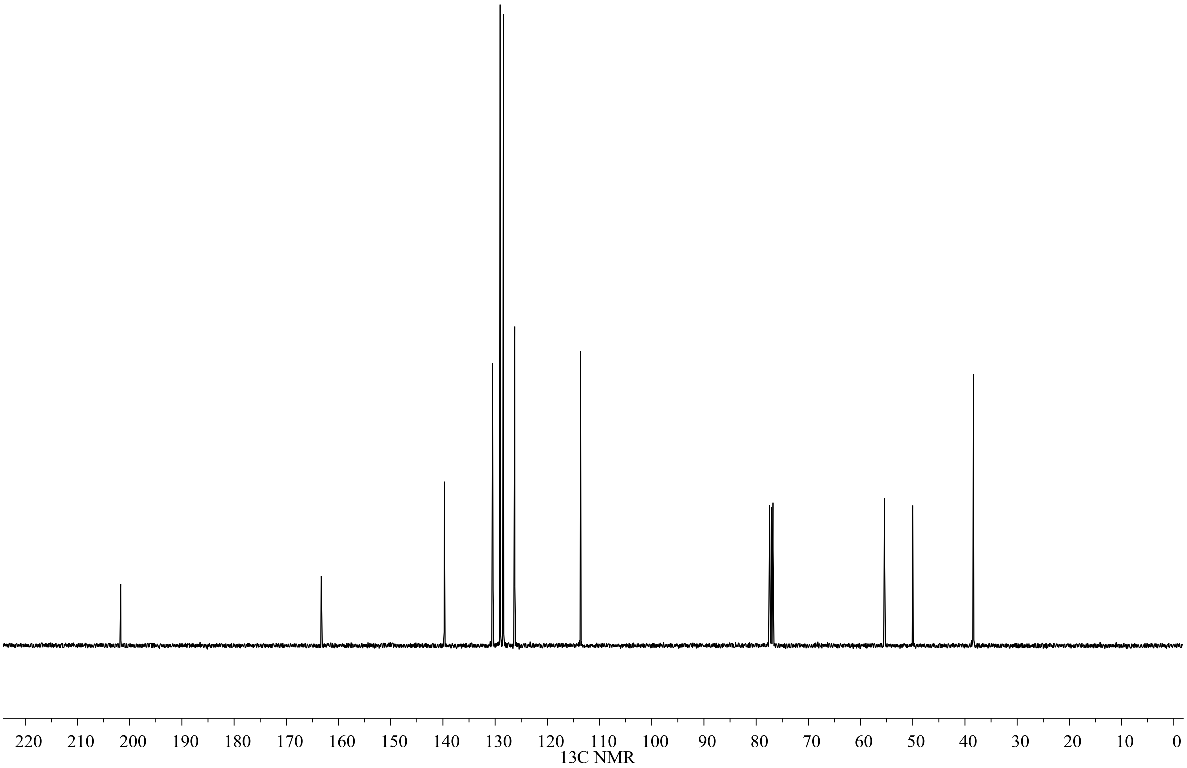

2-Benzyl-1-(4-methoxyphenyl)-3-(p-tolyl)propan-1-one, 21

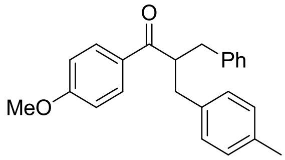

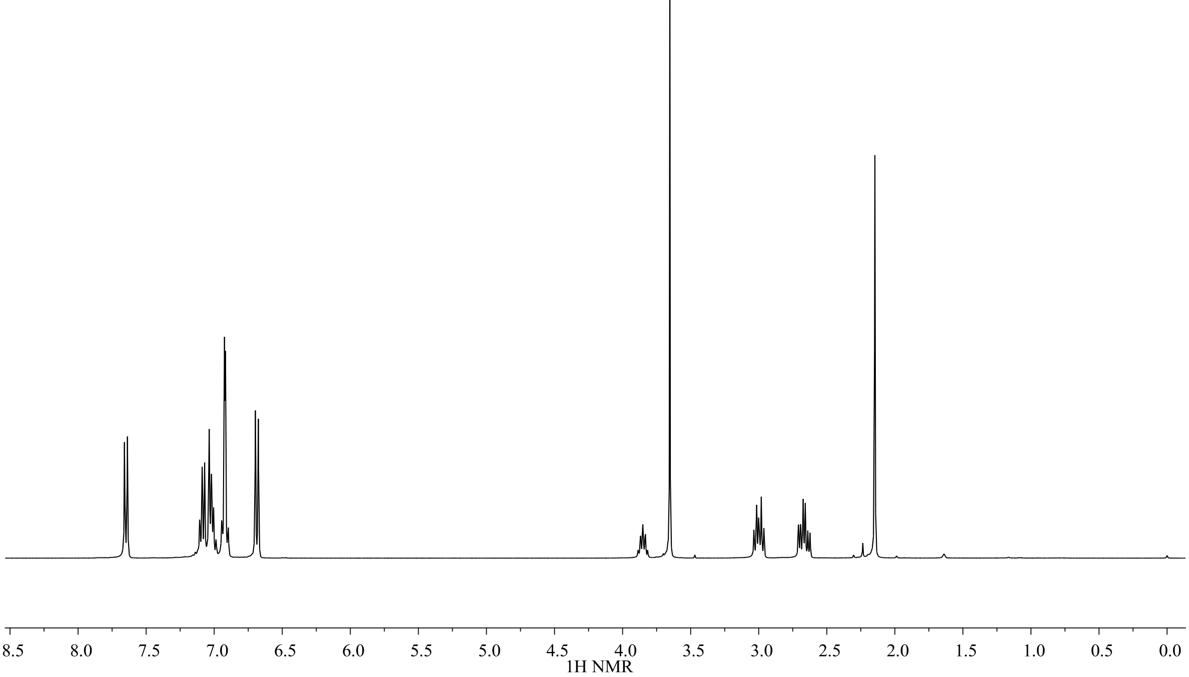

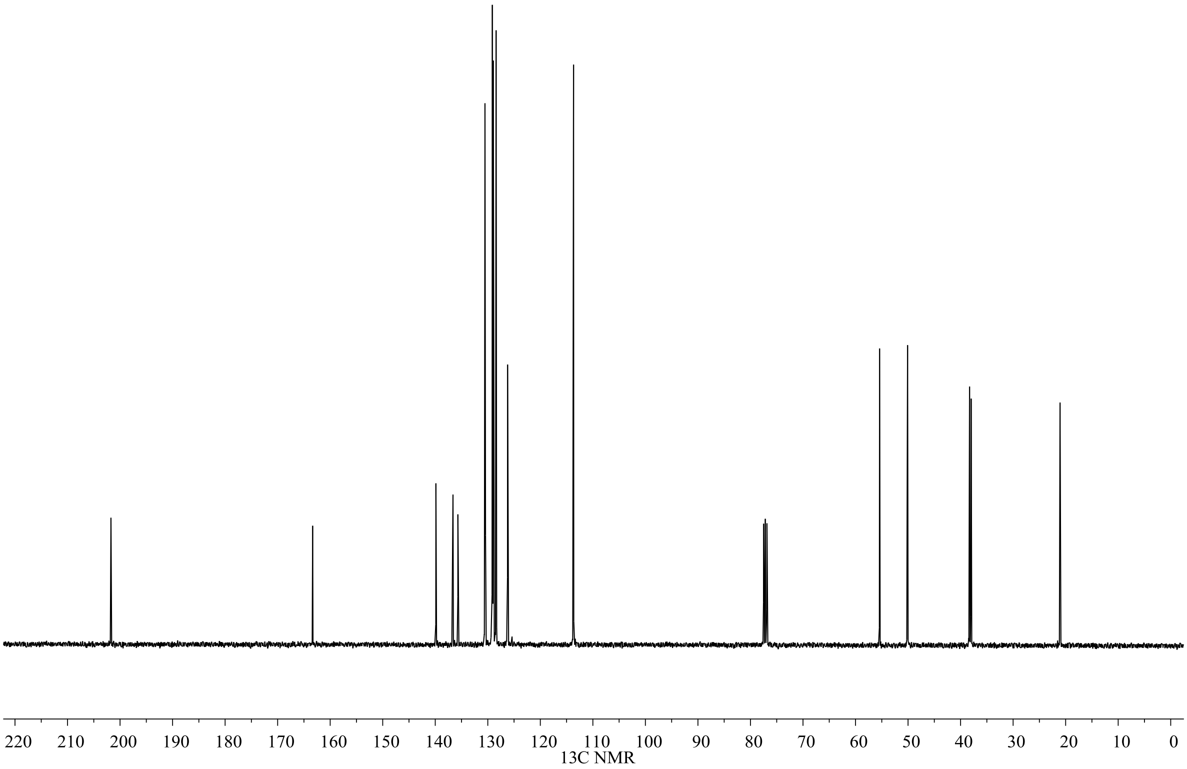

(±)1-(4-Methoxyphenyl)-2-methylhexan-1-one, 22

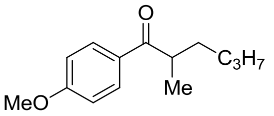

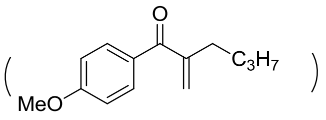

contains 2.5%, inseparable

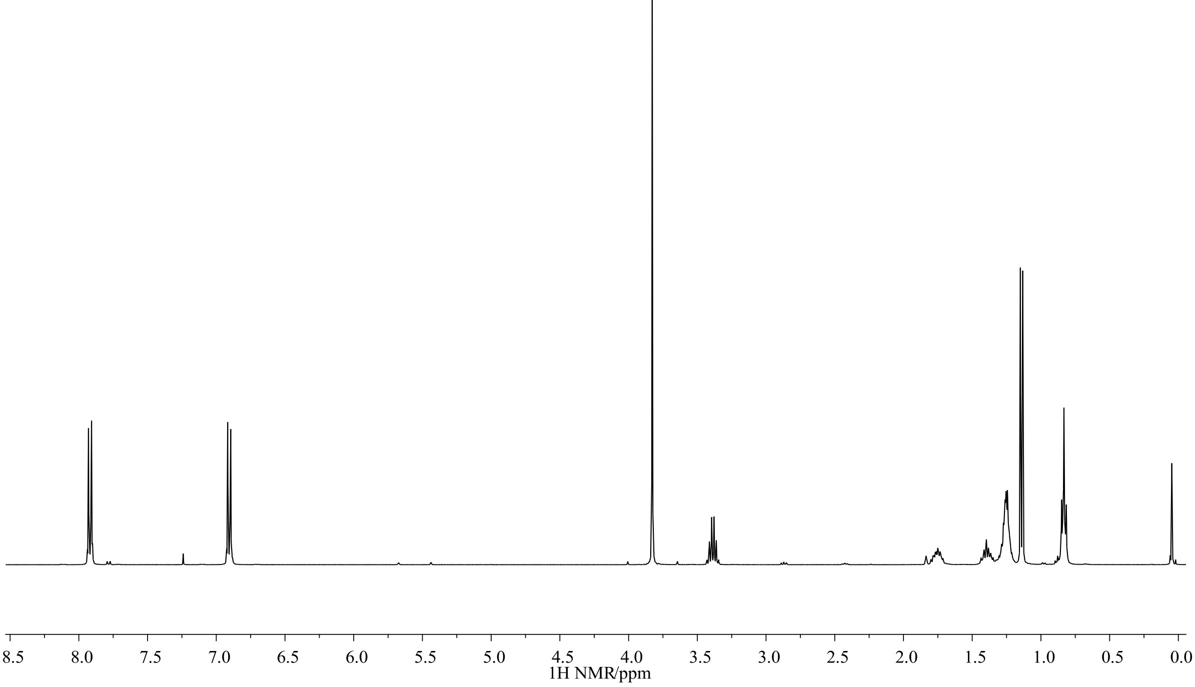

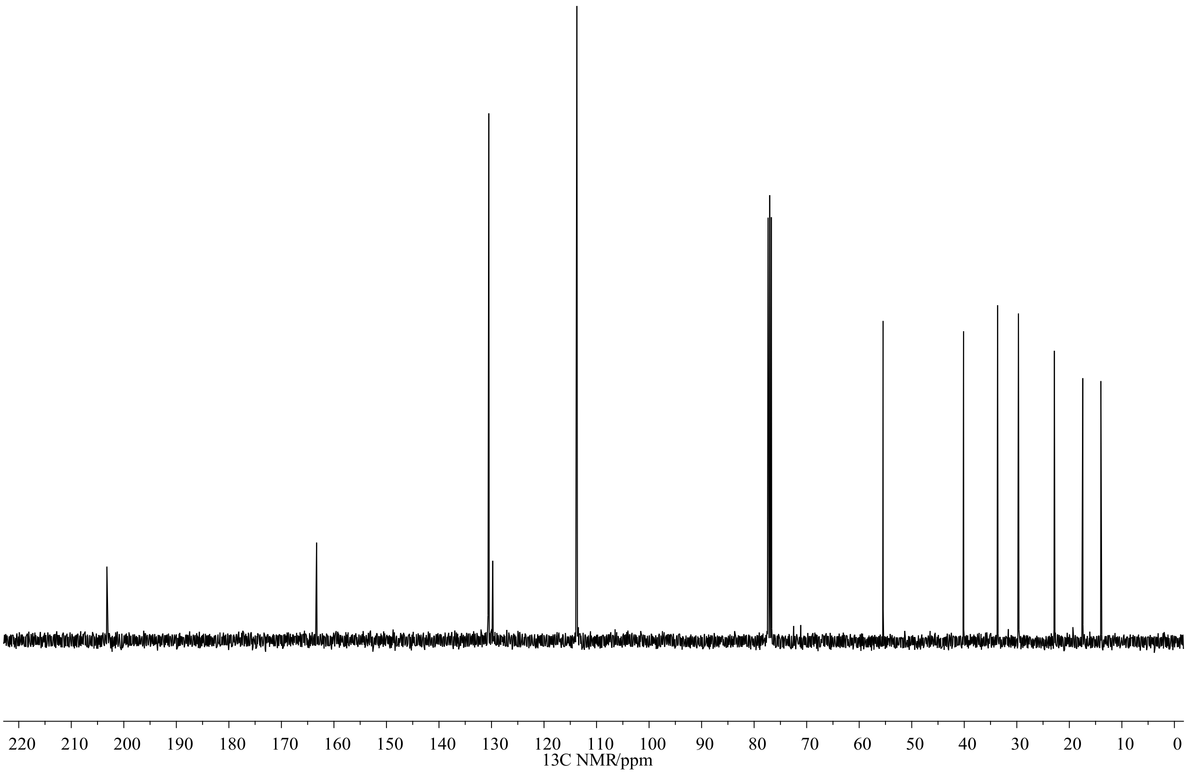

(±)1-(4-Methoxyphenyl)-2-methylhexadecan-1-one, 23

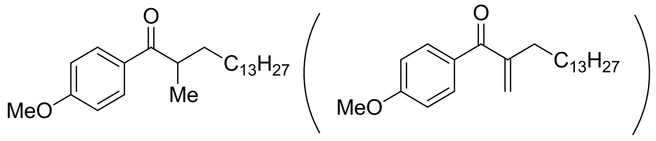

contains 2%, inseparable

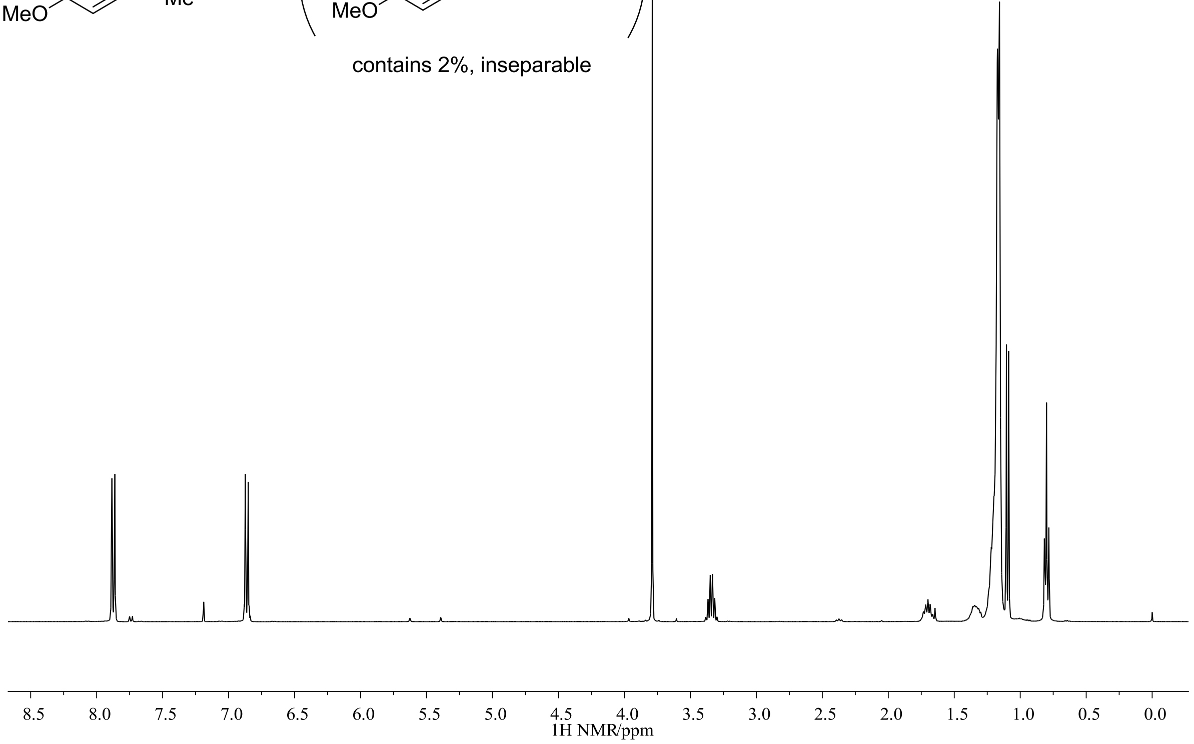

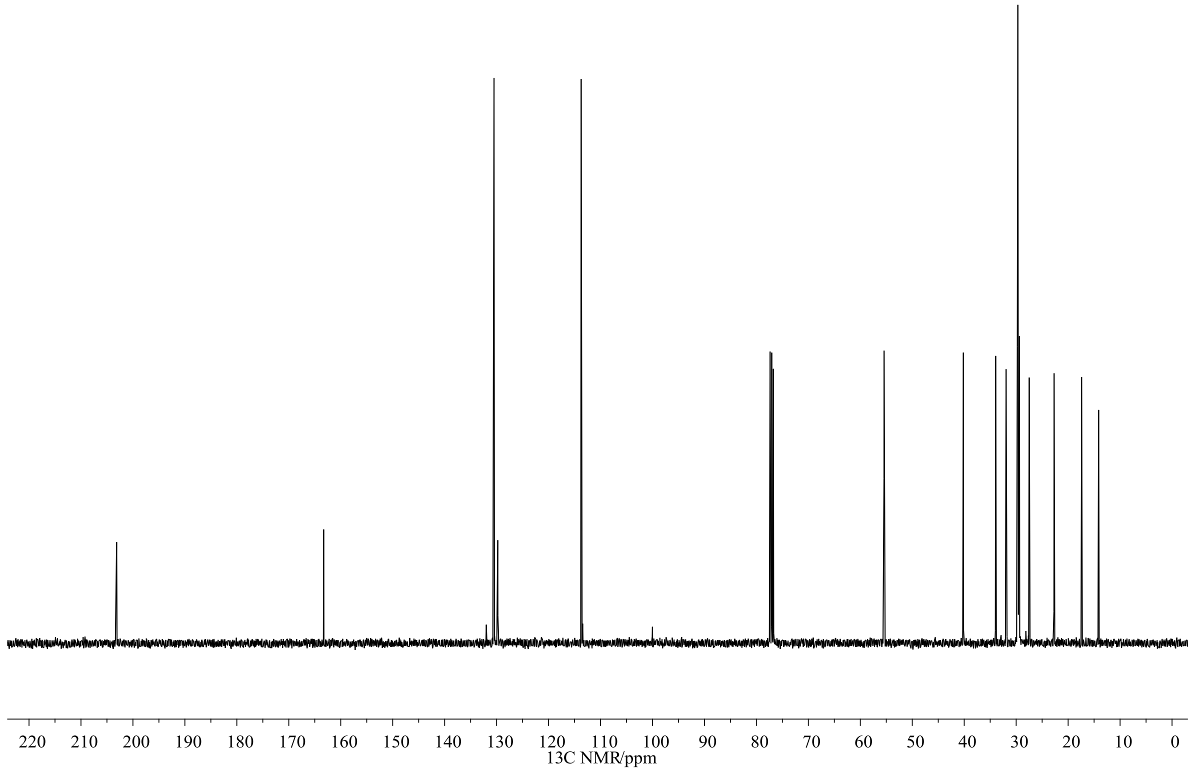

(±)1-(4-methoxyphenyl)-2,4-dimethylpentan-1-one, 24

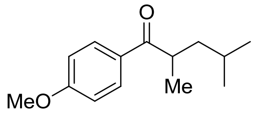

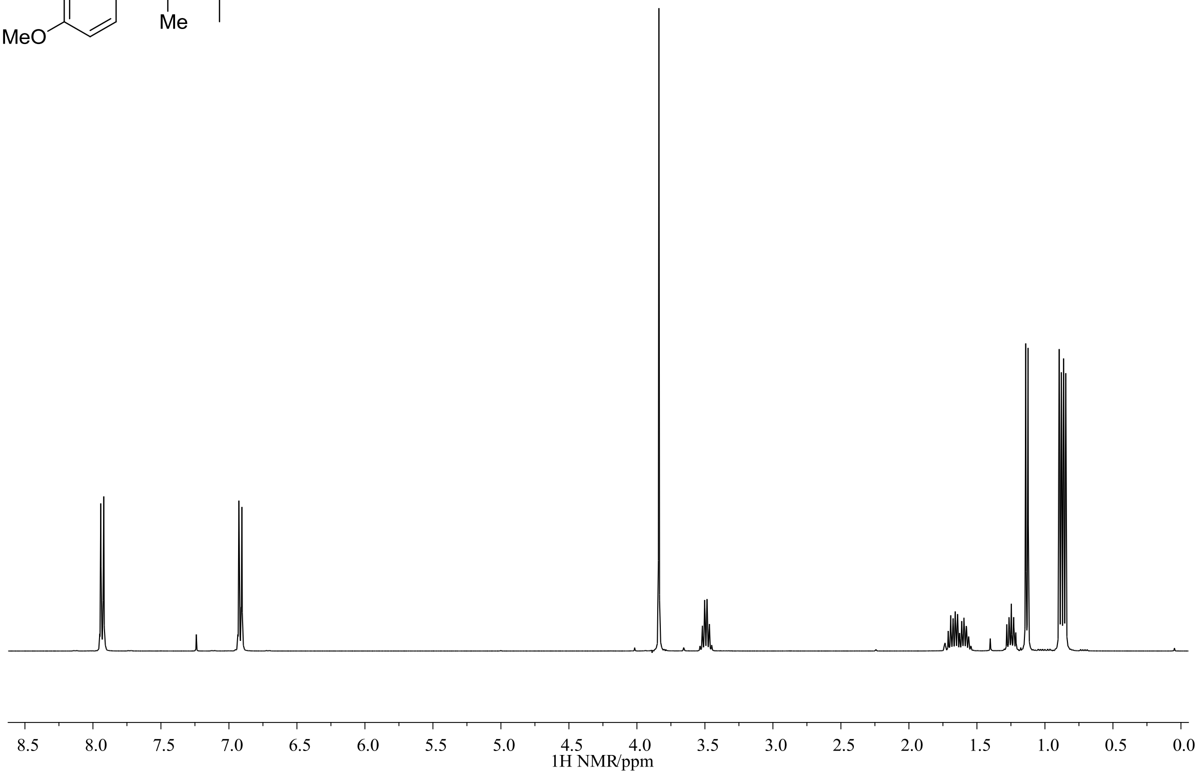

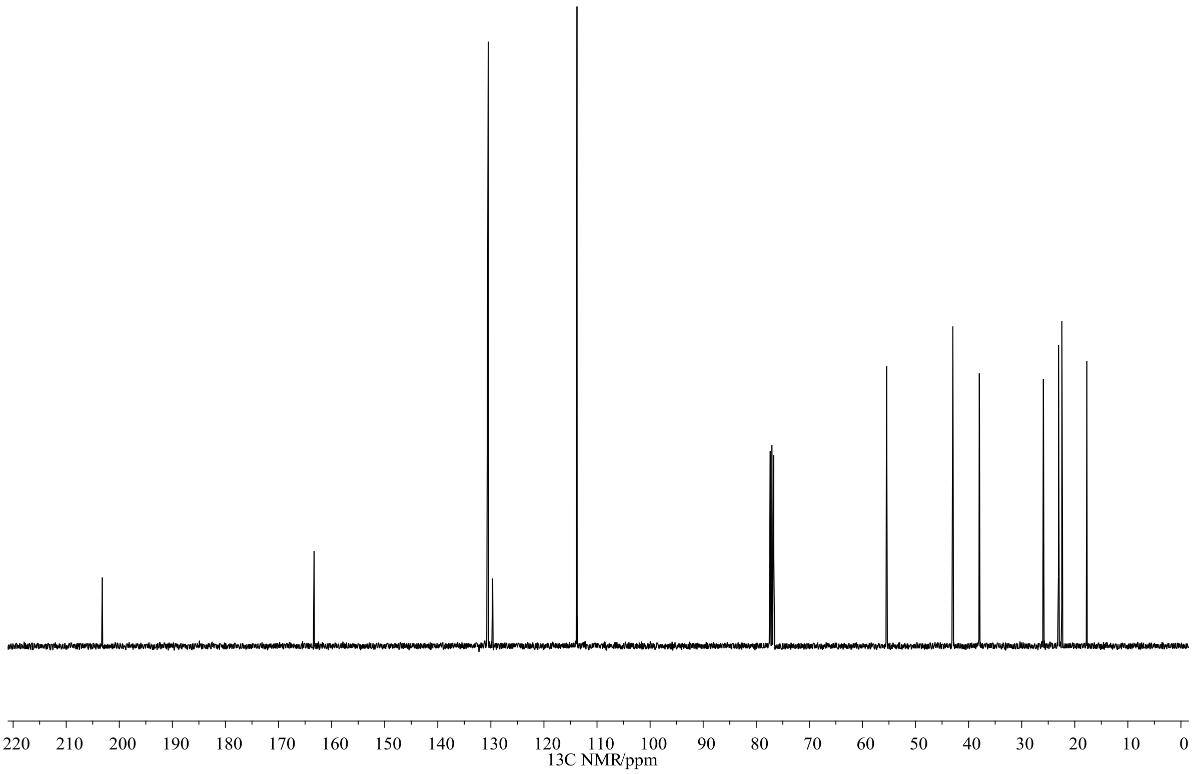

(±)1-(4-Methoxyphenyl)-2,5-dimethylhexan-1-one, 25

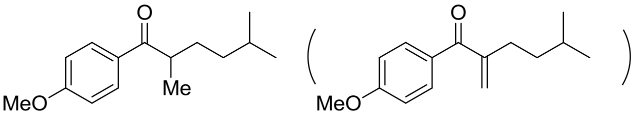

contains 4%, inseparable

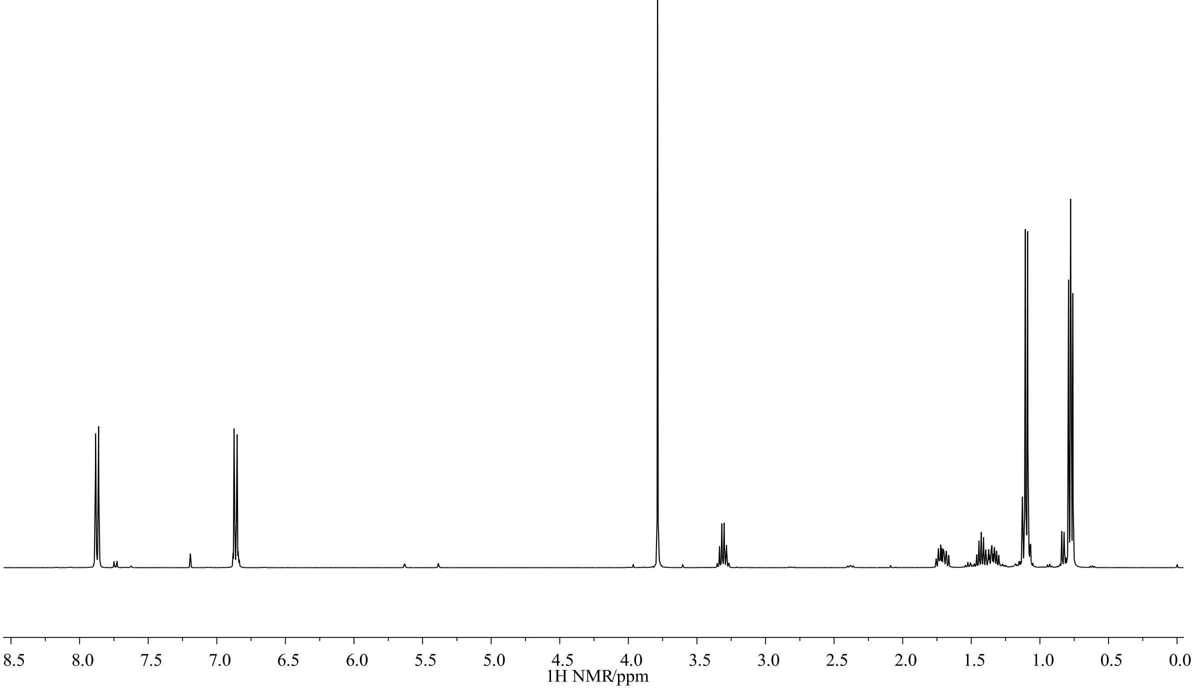

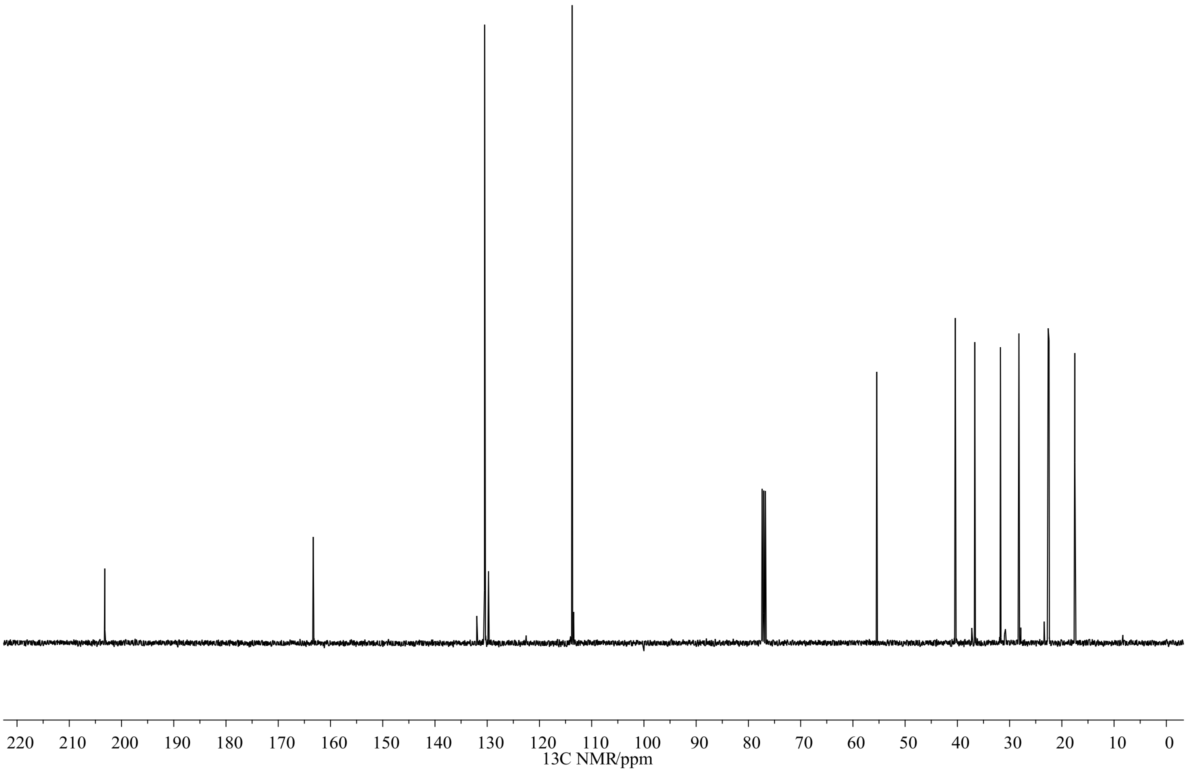

1-(4-Methoxyphenyl)-2,5,5-trimethylhexan-1-one, 26

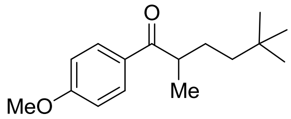

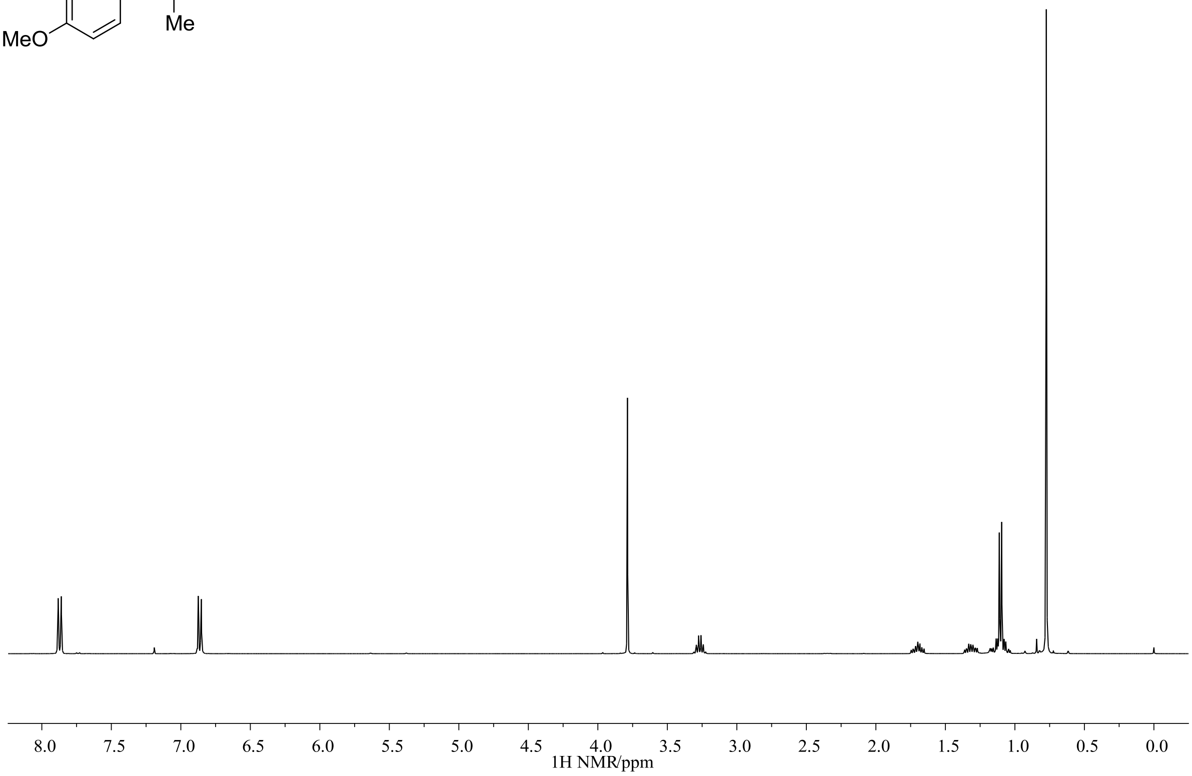

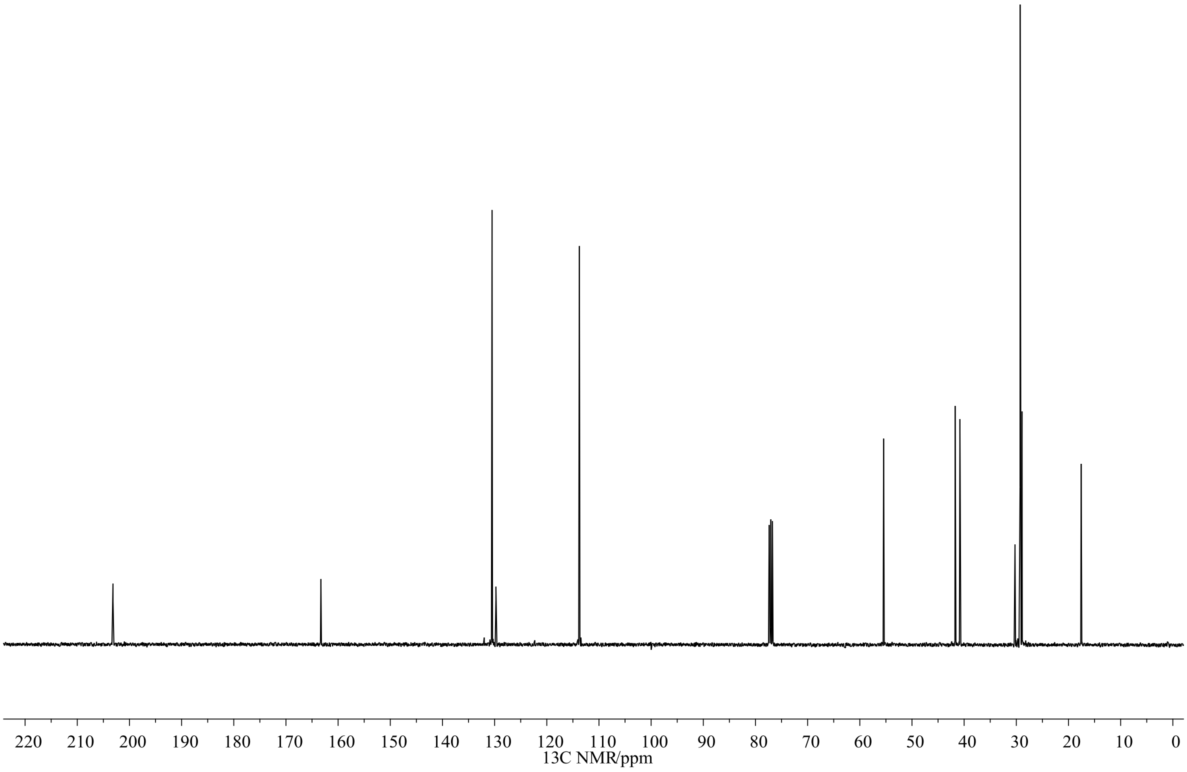

(±)3-(2-methoxyphenyl)-1-(4-methoxyphenyl)-2-methylpropan-1-one, 28

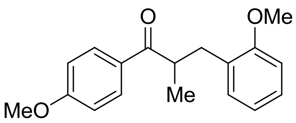

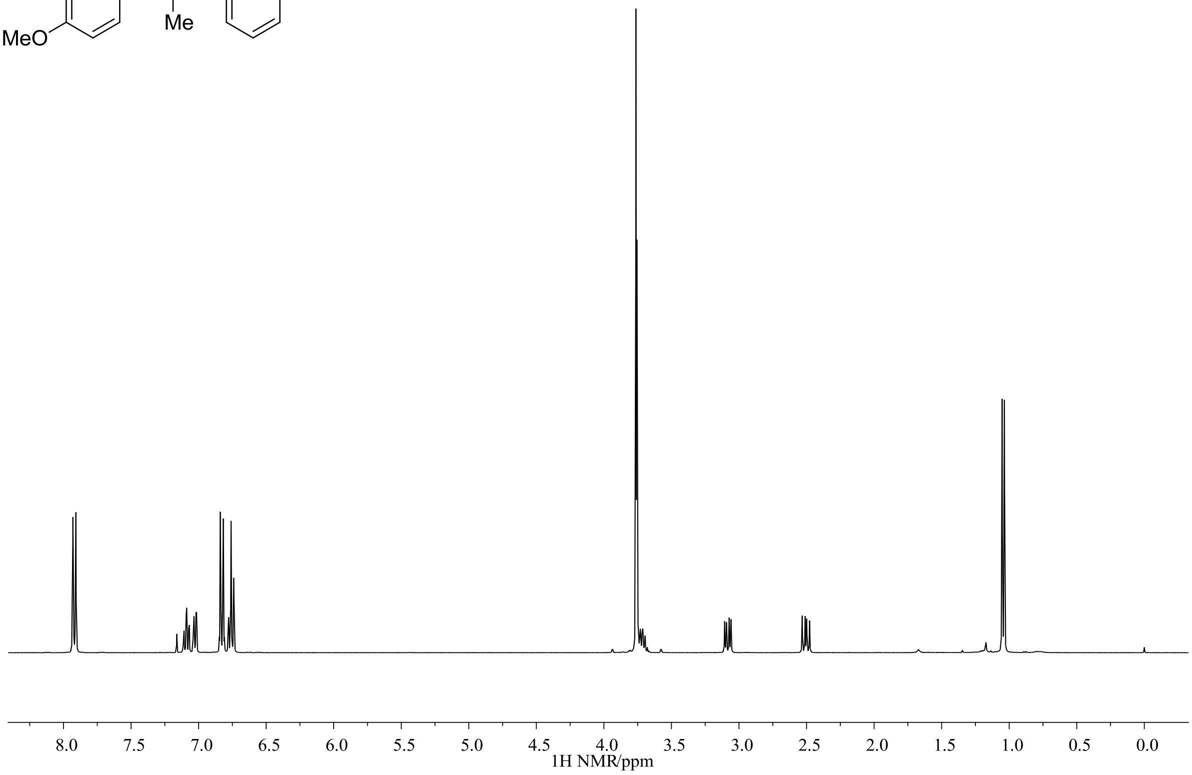

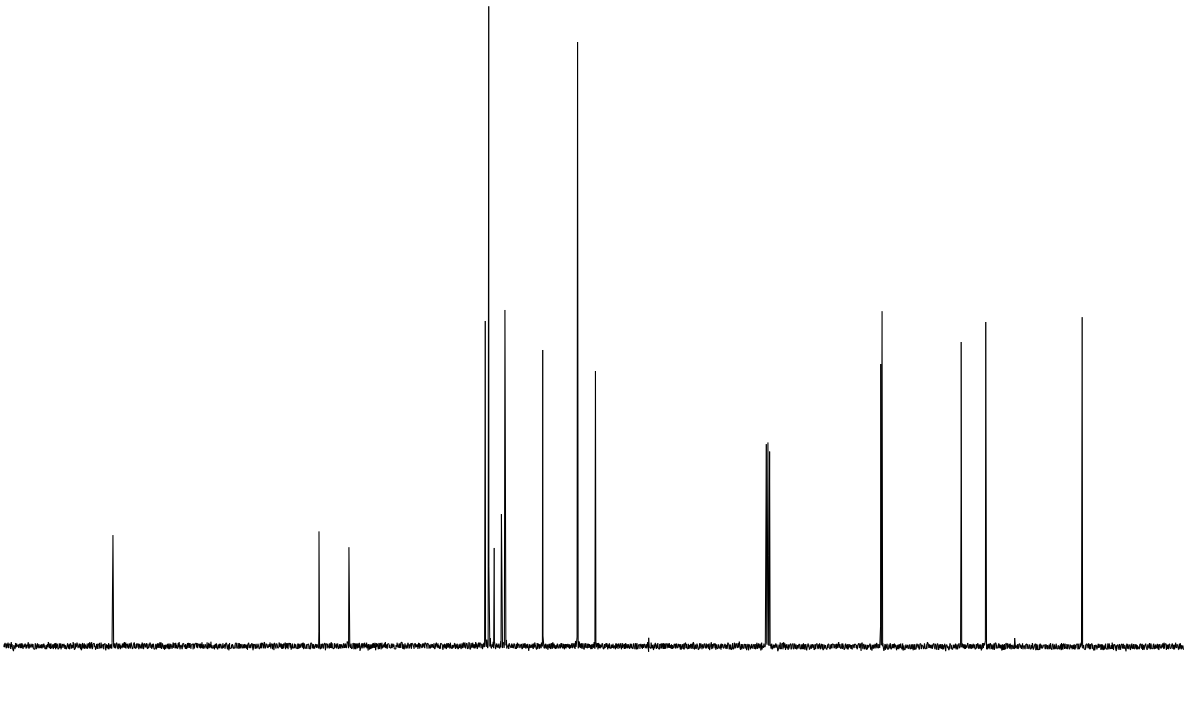

(±)1-(4-methoxyphenyl)-2-methyl-3-(4-(trifluoromethyl)phenyl)propan-1-one, 29

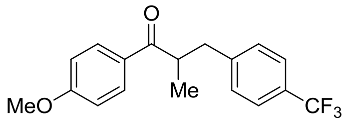

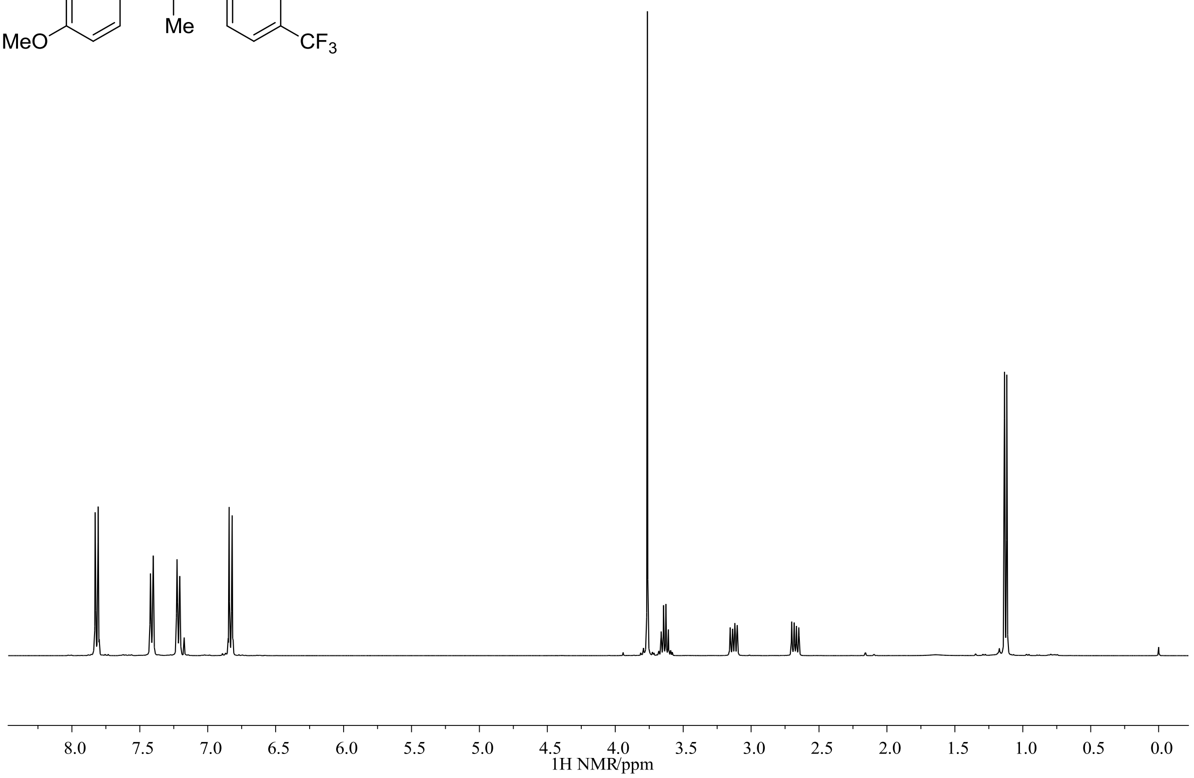

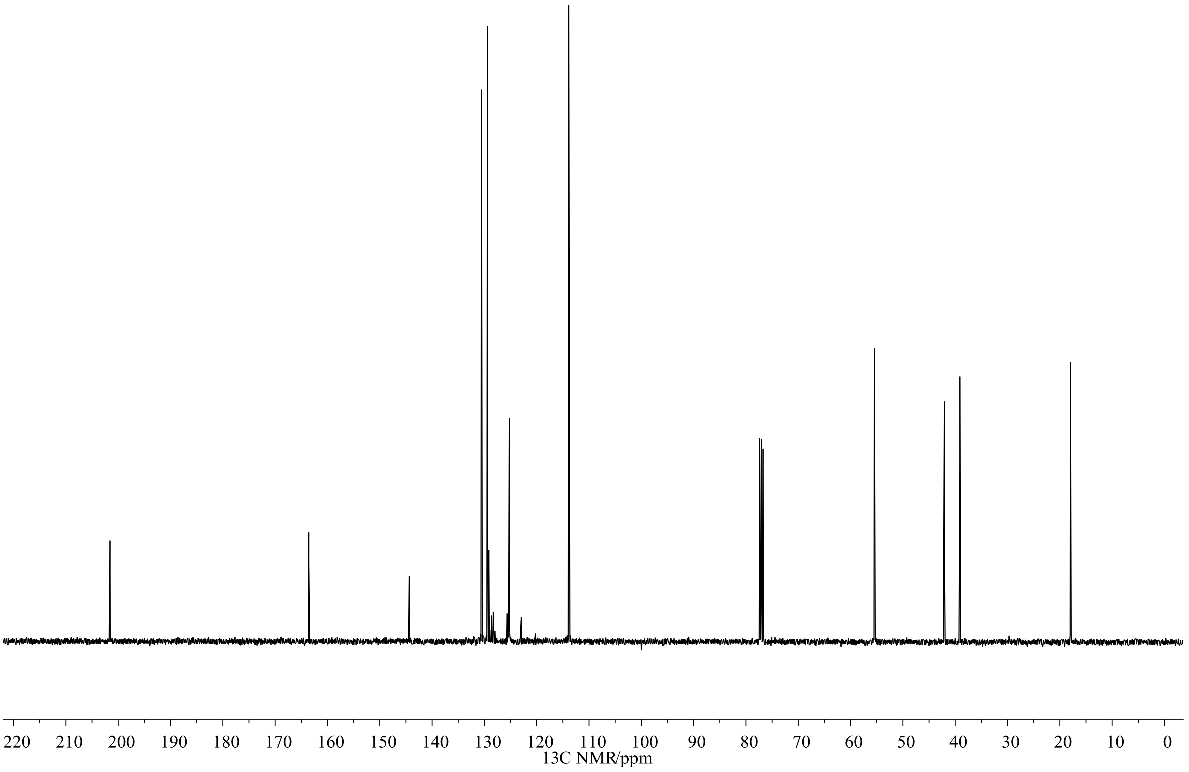

(±)3-(4-bromophenyl)-1-(4-methoxyphenyl)-2-methylpropan-1-one, 30

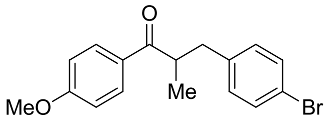

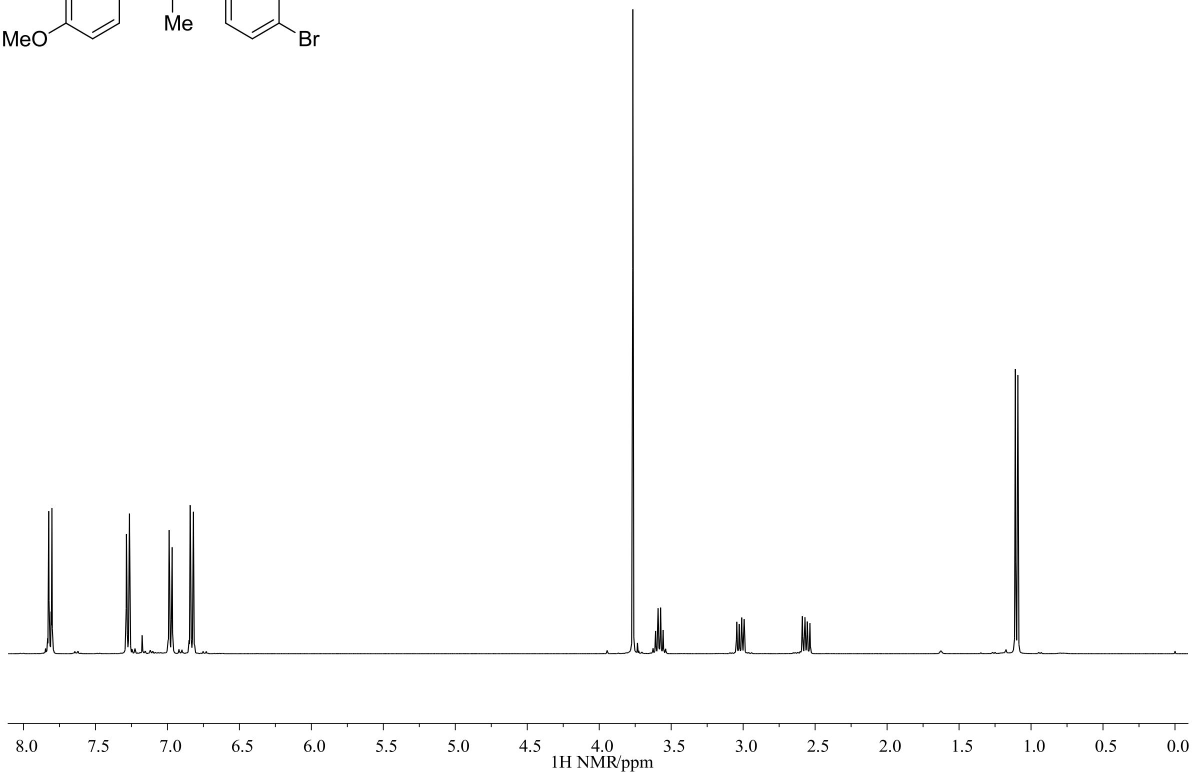

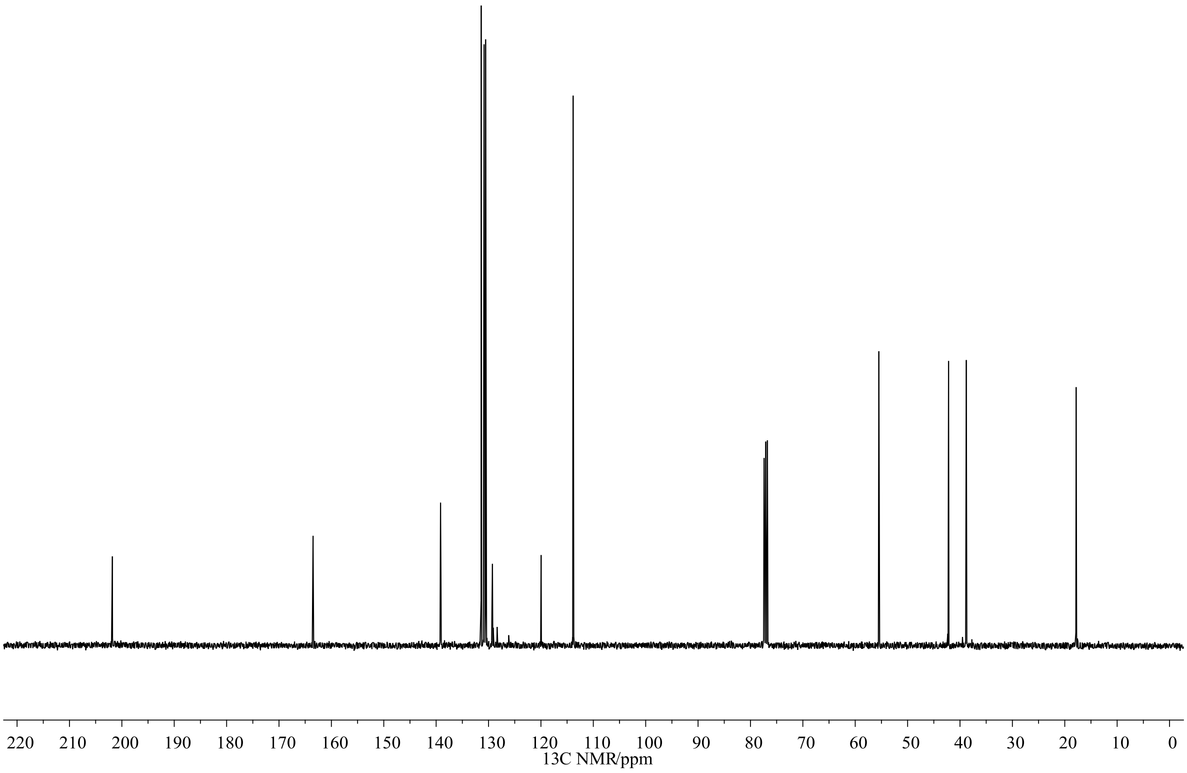

(±)4-Methoxyphenyl 2-methyl-3-phenylpropanoate, 31

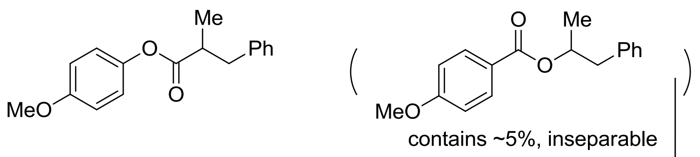

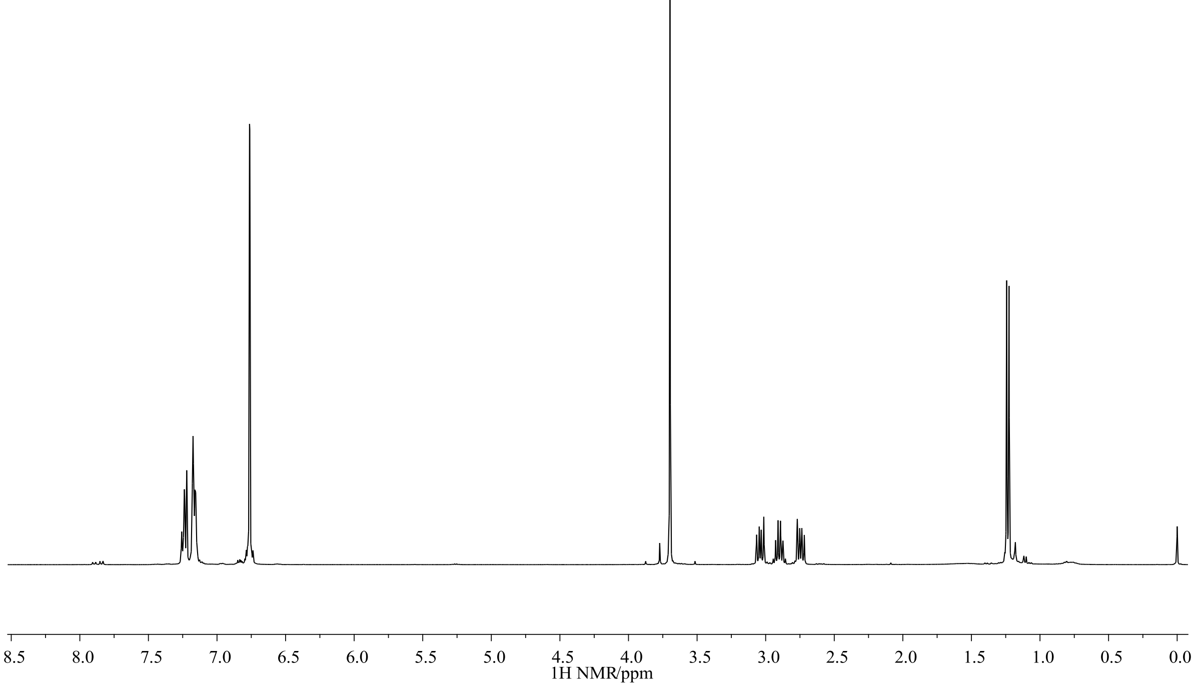

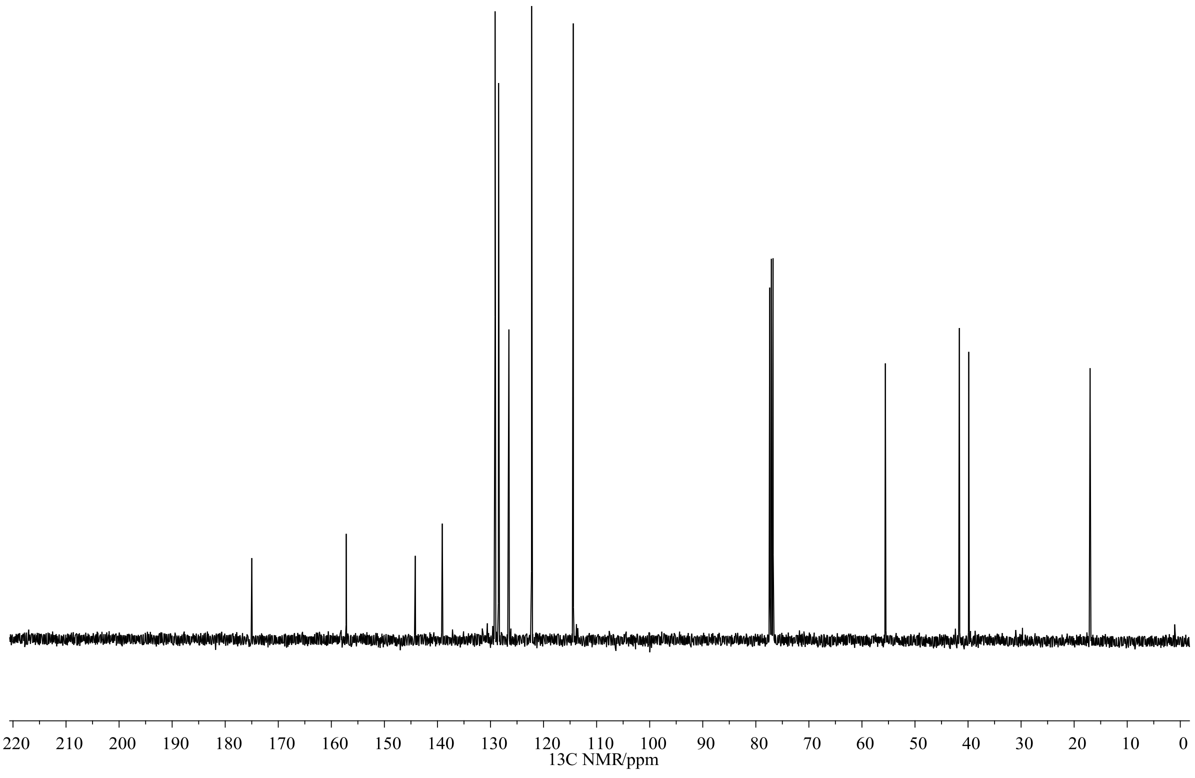

(±)4-Methoxyphenyl 2-methylhexanoate, 32

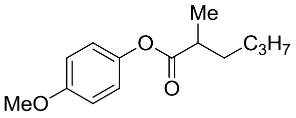

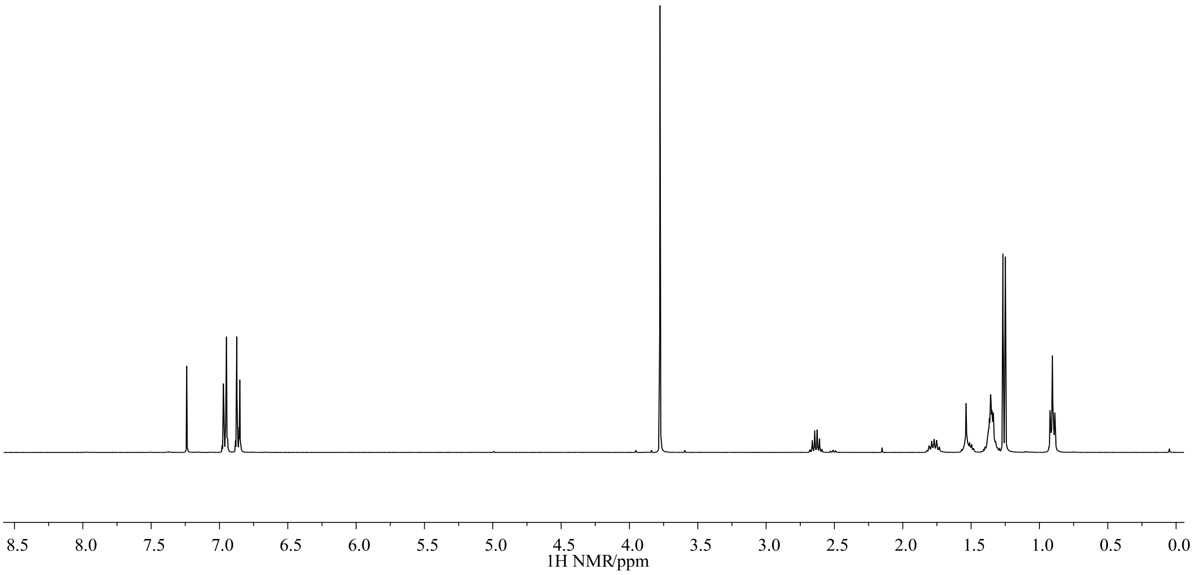

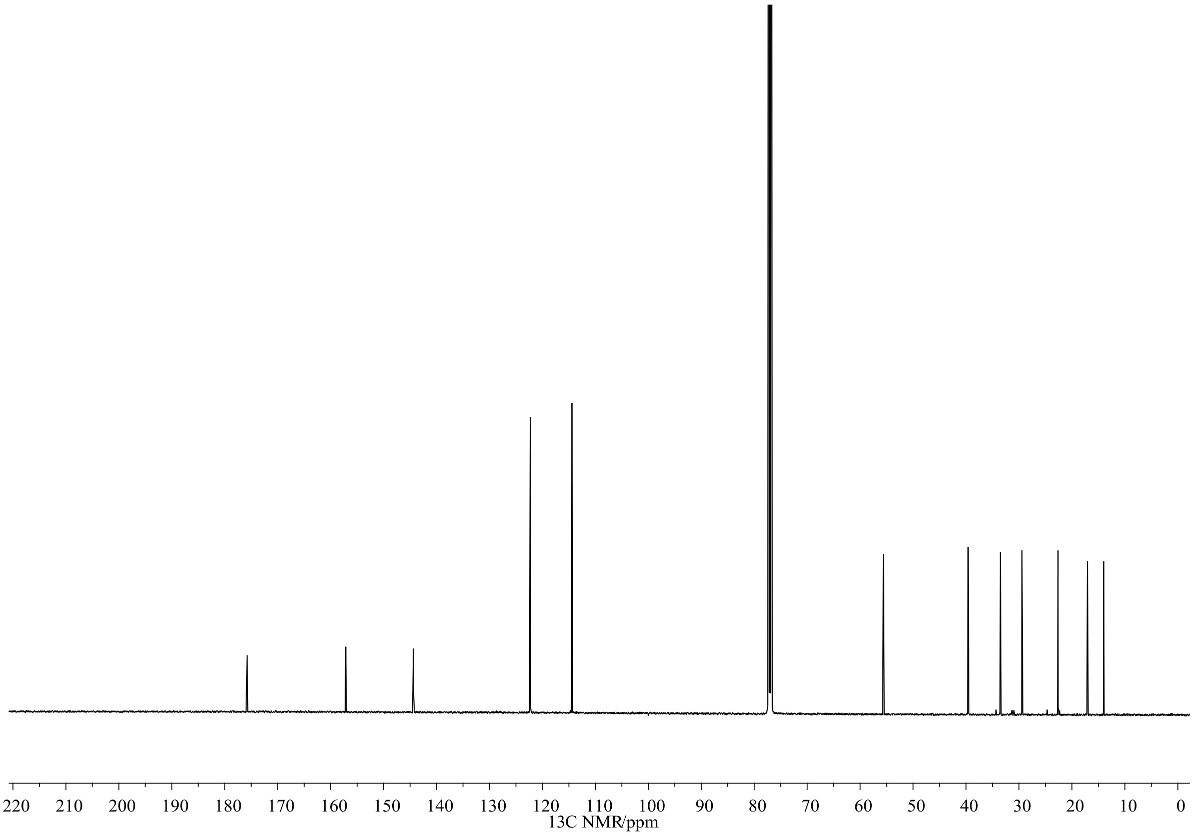

(±)4-Methoxyphenyl 2,4-dimethylpentanoate, 33

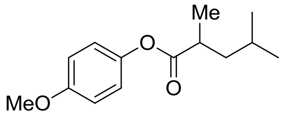

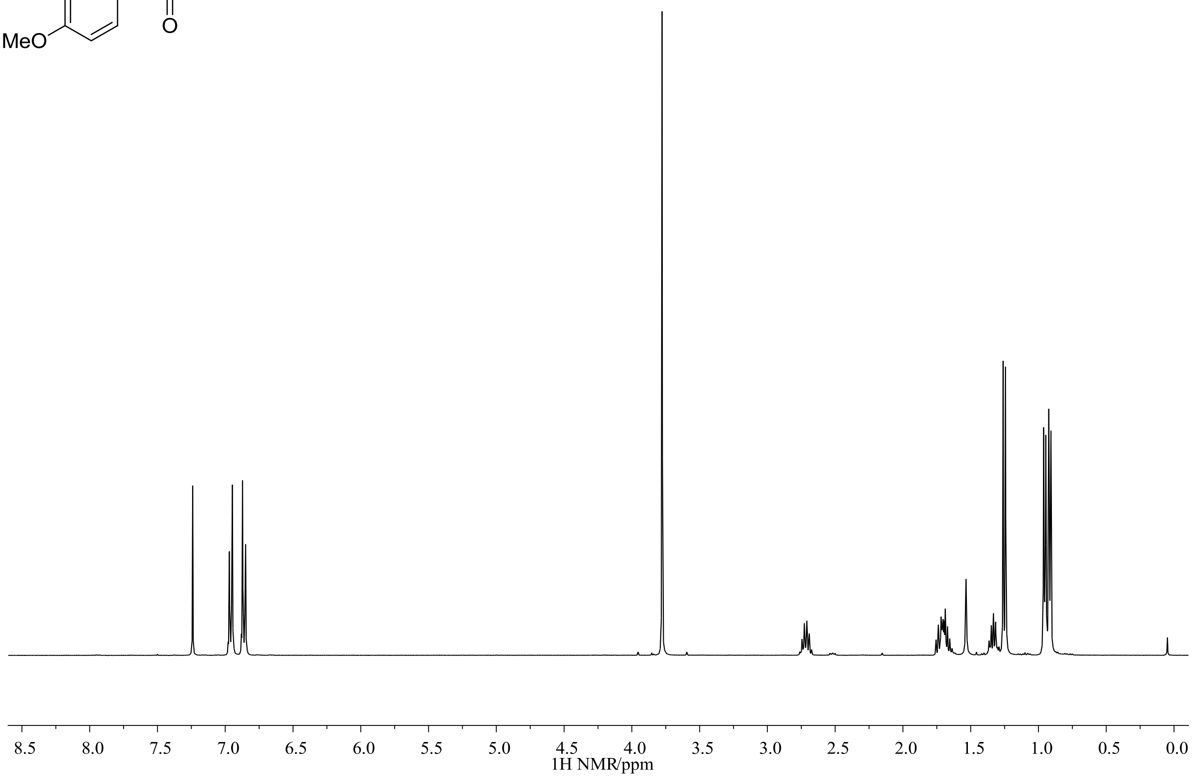

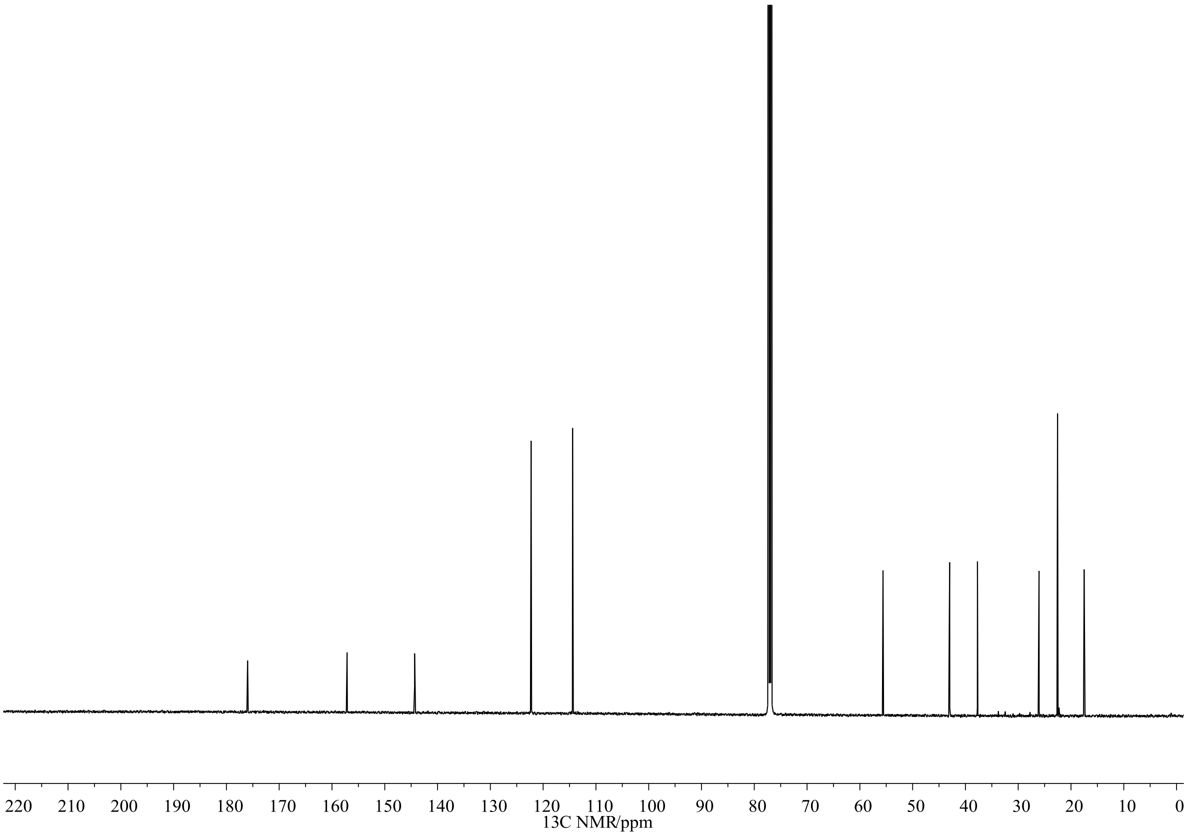

(±)4-Methoxyphenyl 3-(4-bromophenyl)-2-methylpropanoate, 34

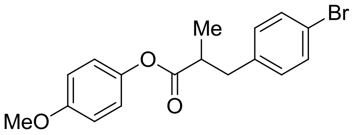

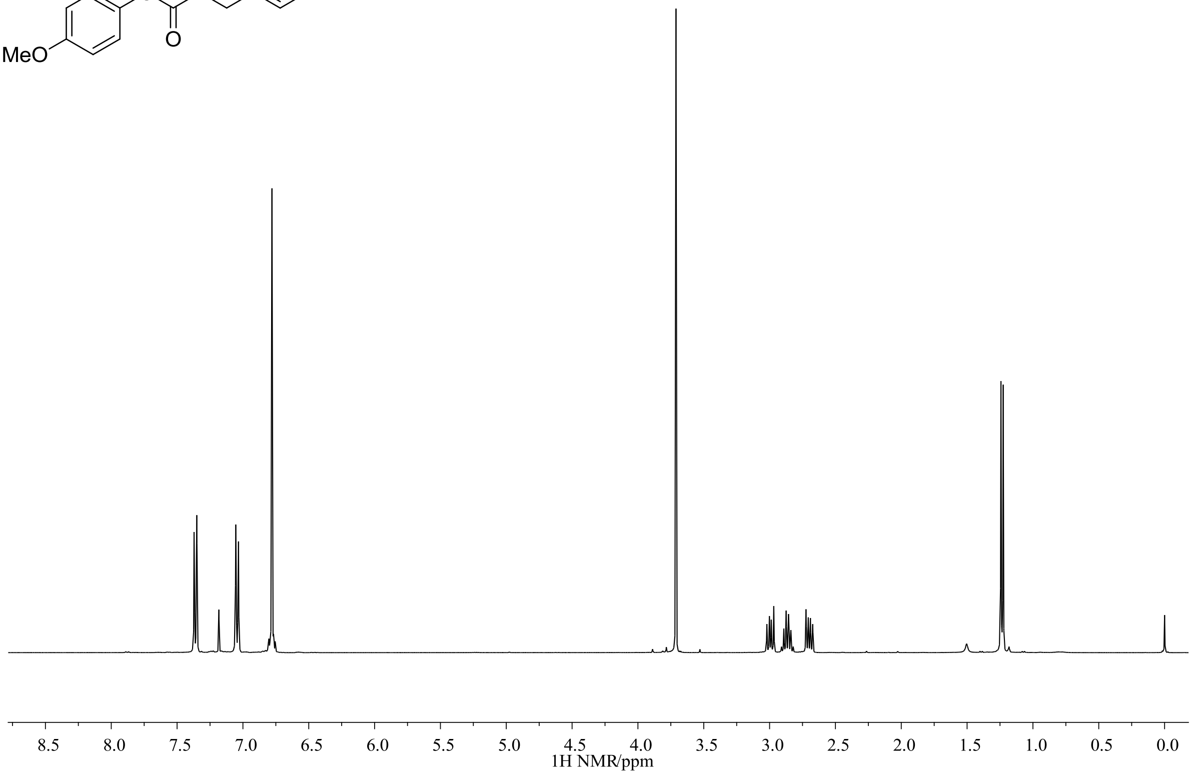

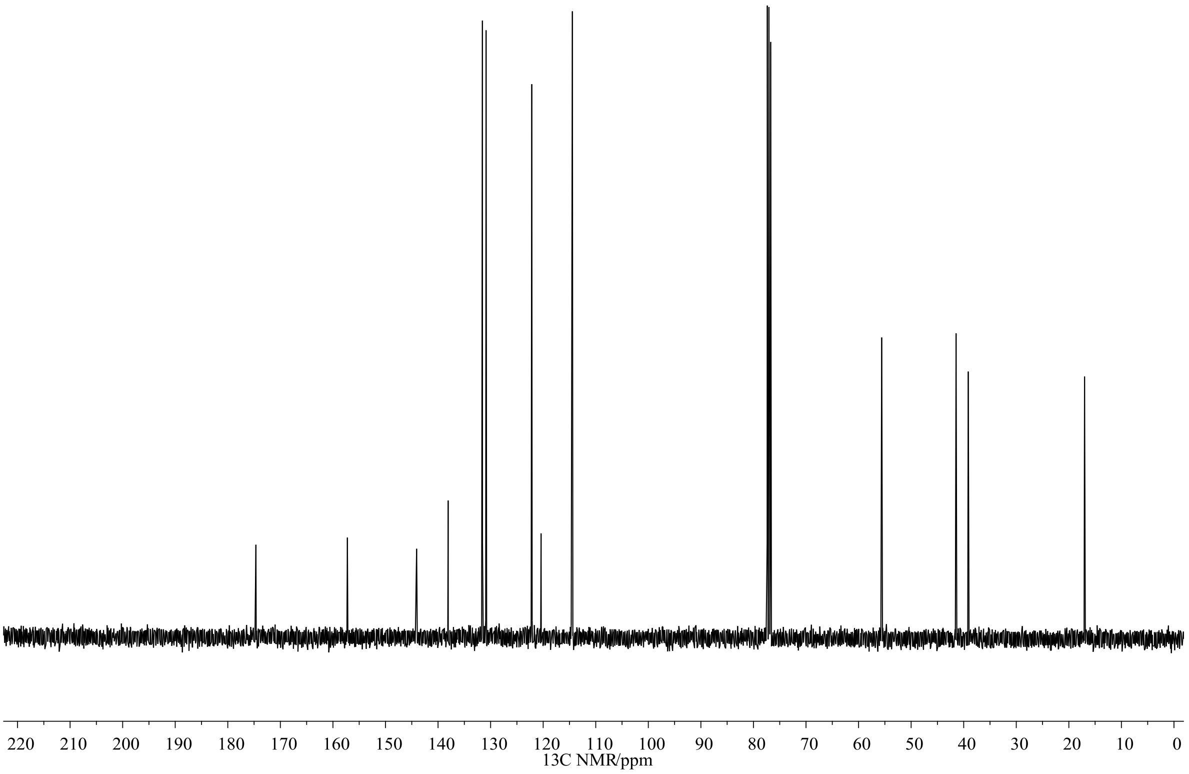

4-Methoxyphenyl 2-benzyloxirane-2-carboxylate, 35

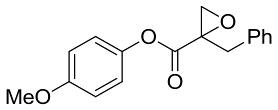

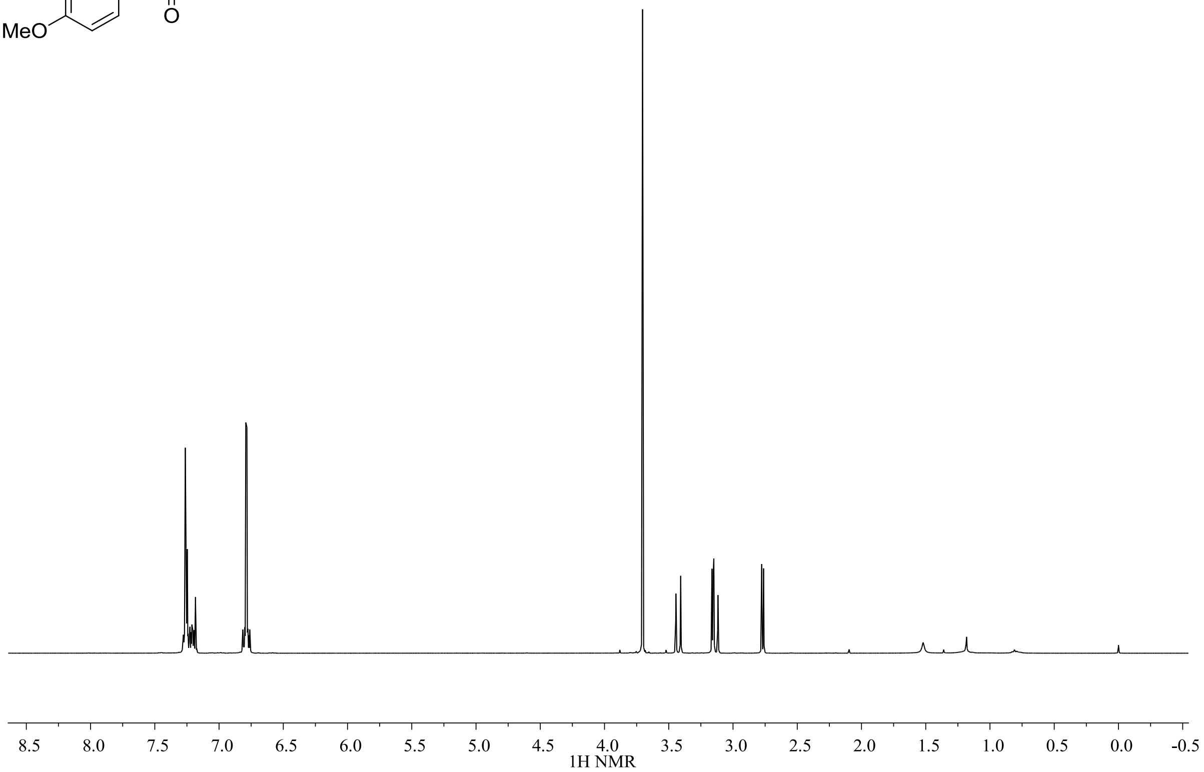

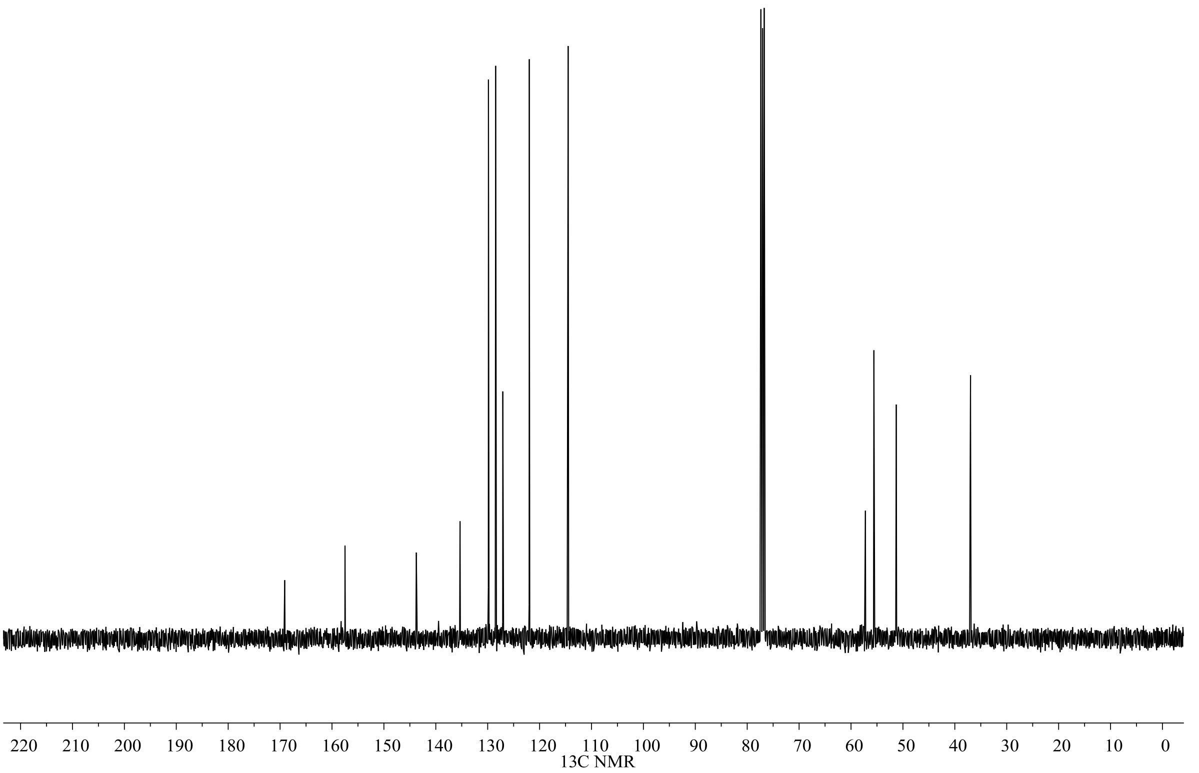

4-Methoxyphenyl 2-isopentyloxirane-2-carboxylate, 36

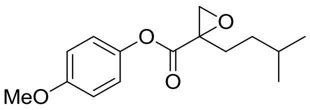

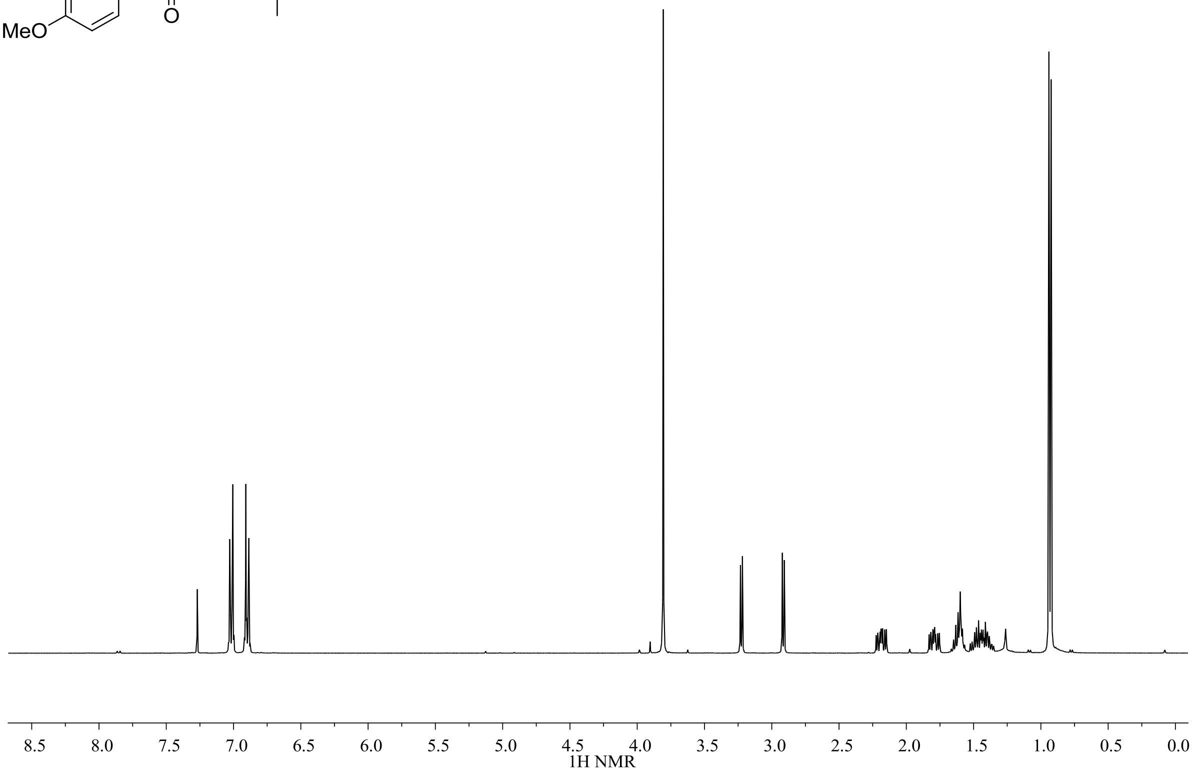

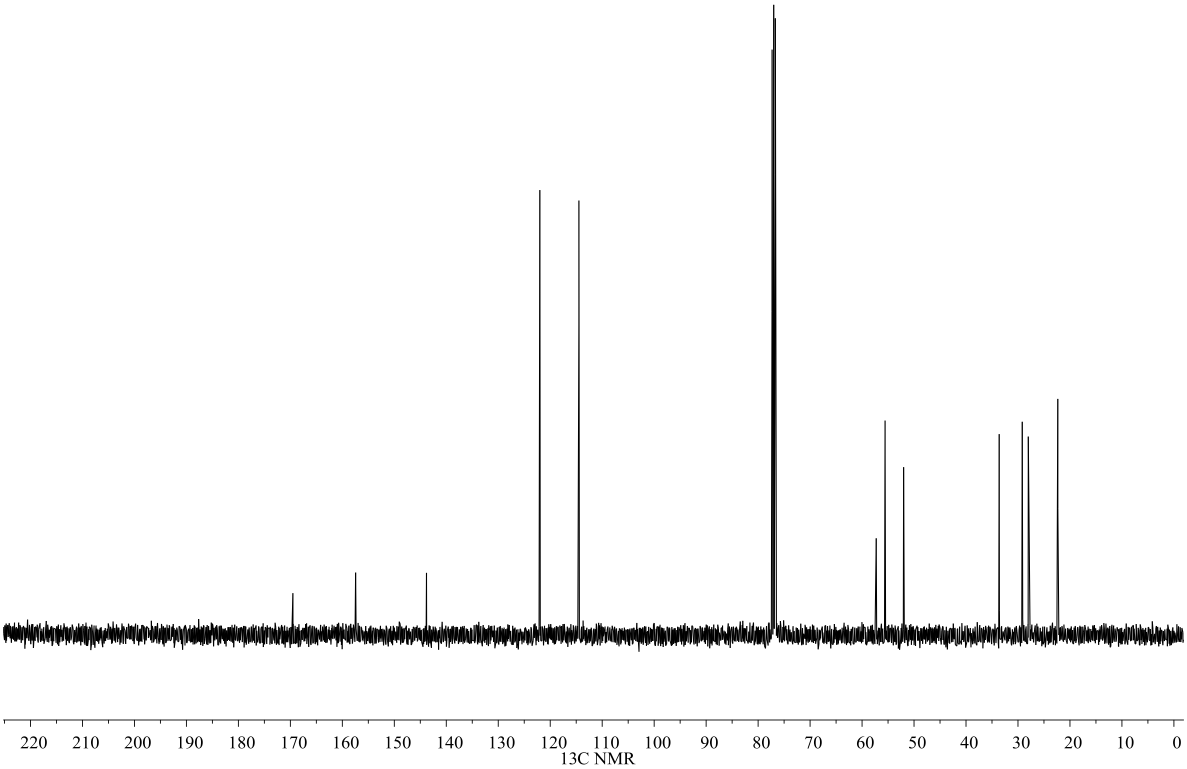

3,5-Dibenzyl-2-cyclopropyl-6-(4-methoxyphenyl)pyridine, 37

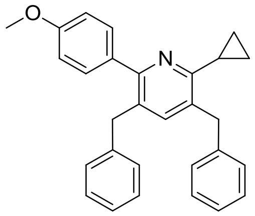

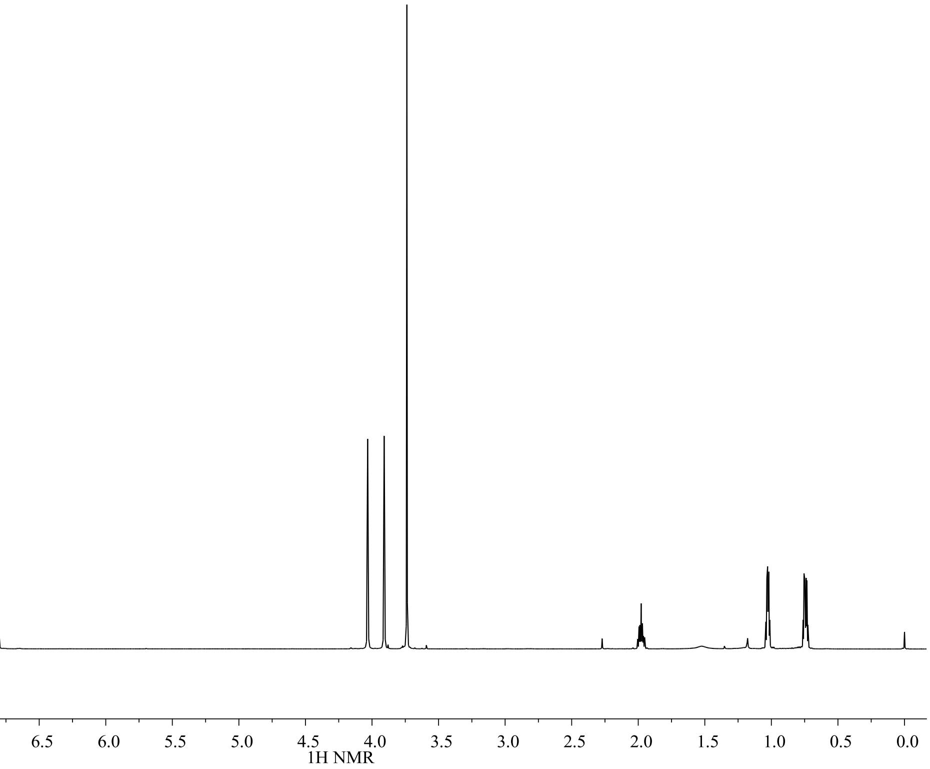

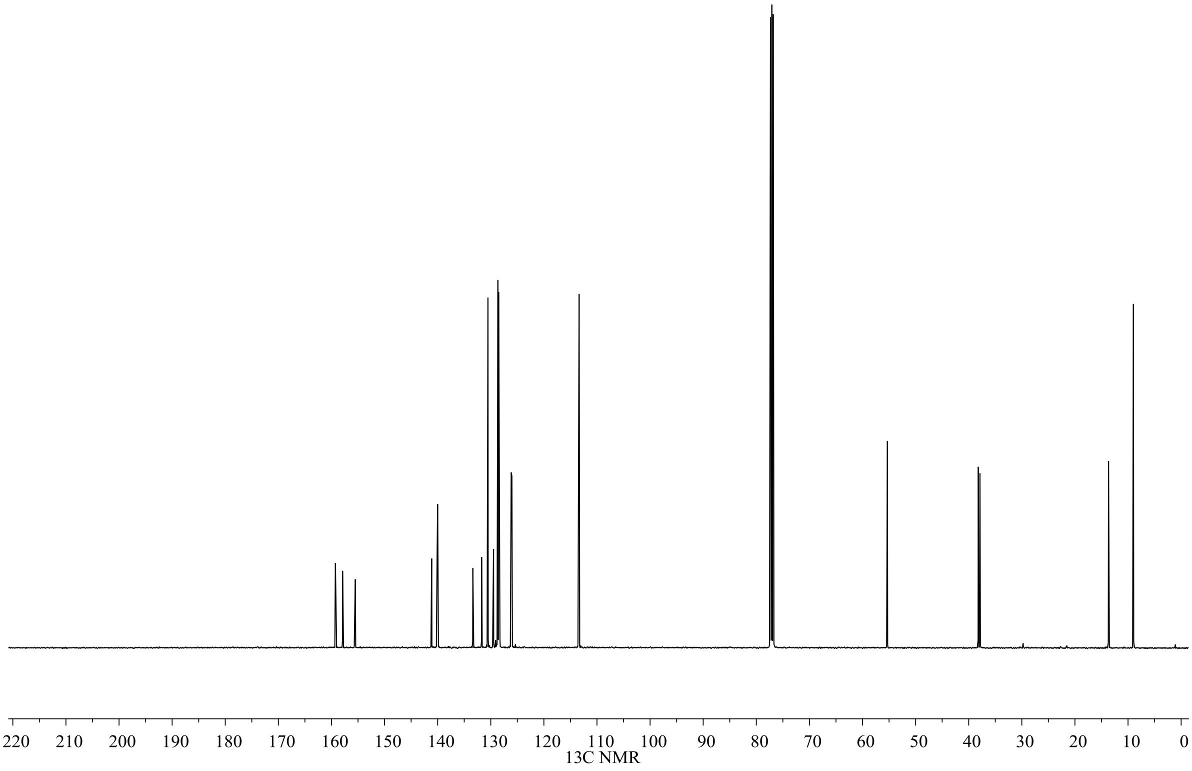

3,5-Dibenzyl-2-(4-methoxyphenyl)-6-phenylpyridine, 38

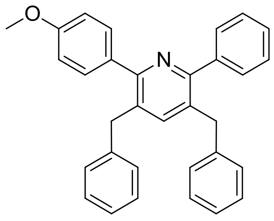

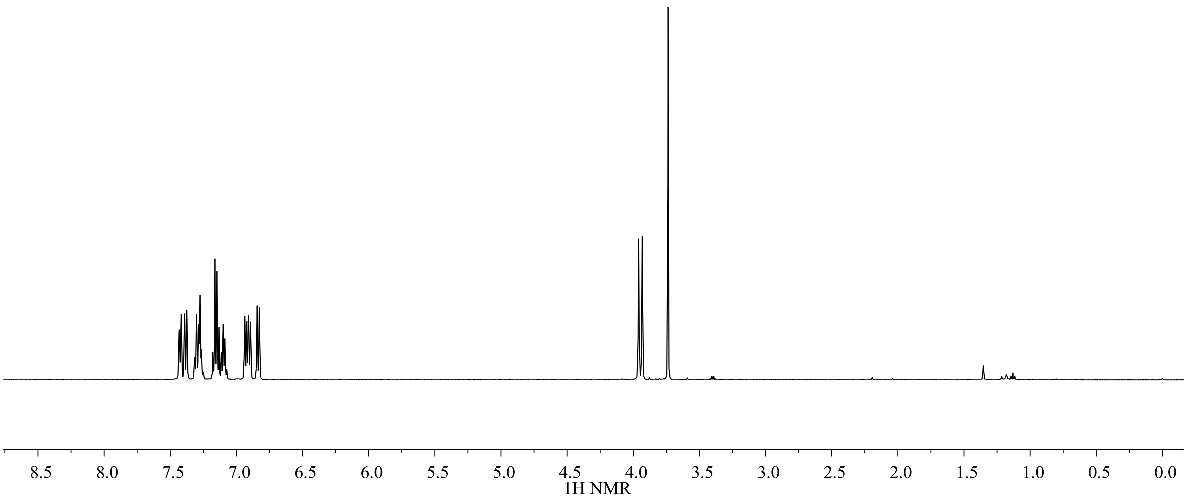

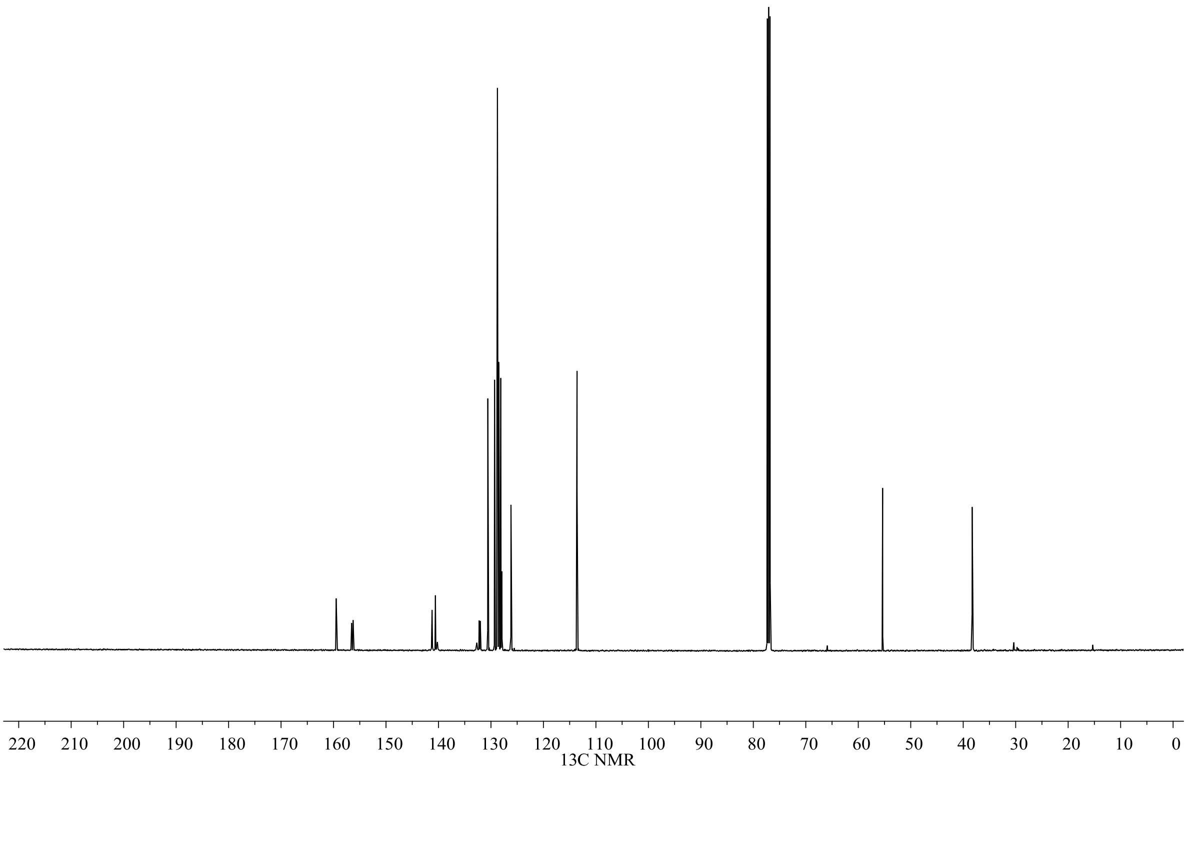

3,5-Dibenzyl-2,6-bis(4-methoxyphenyl)pyridine, 39

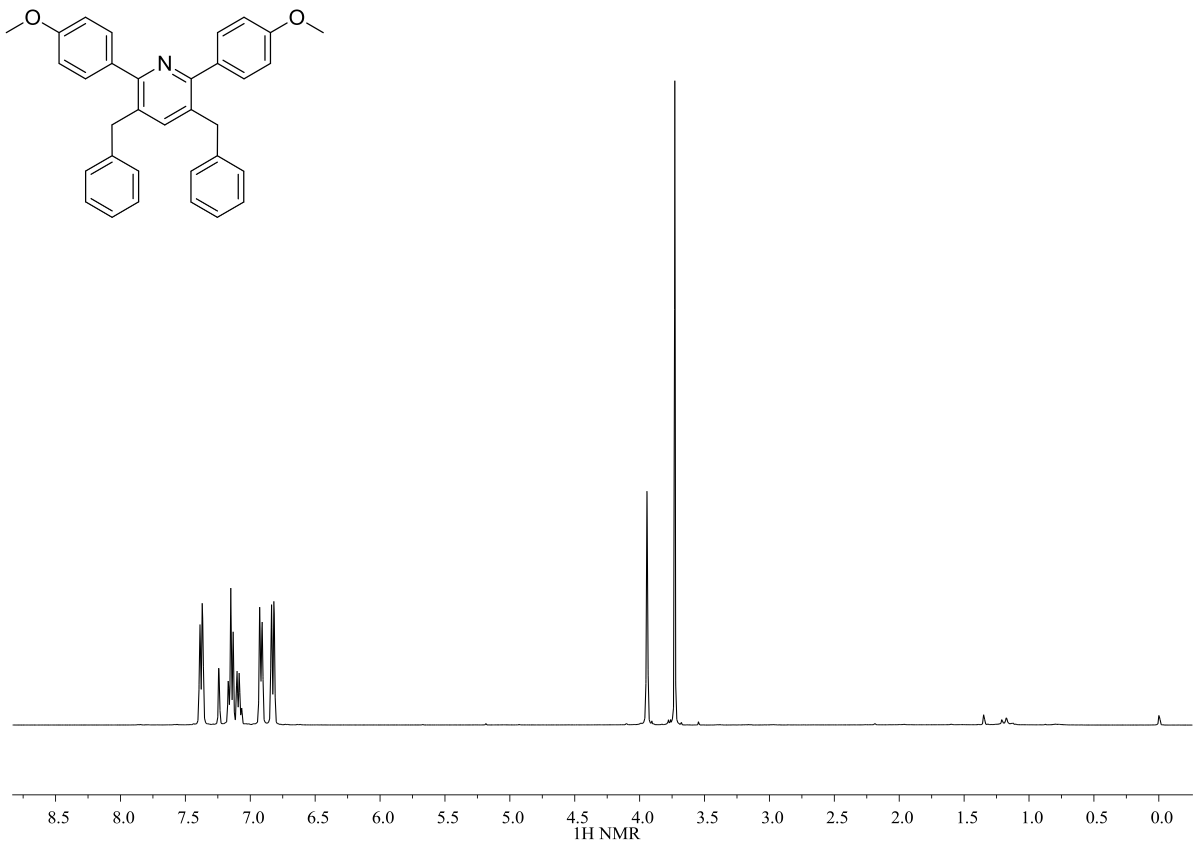

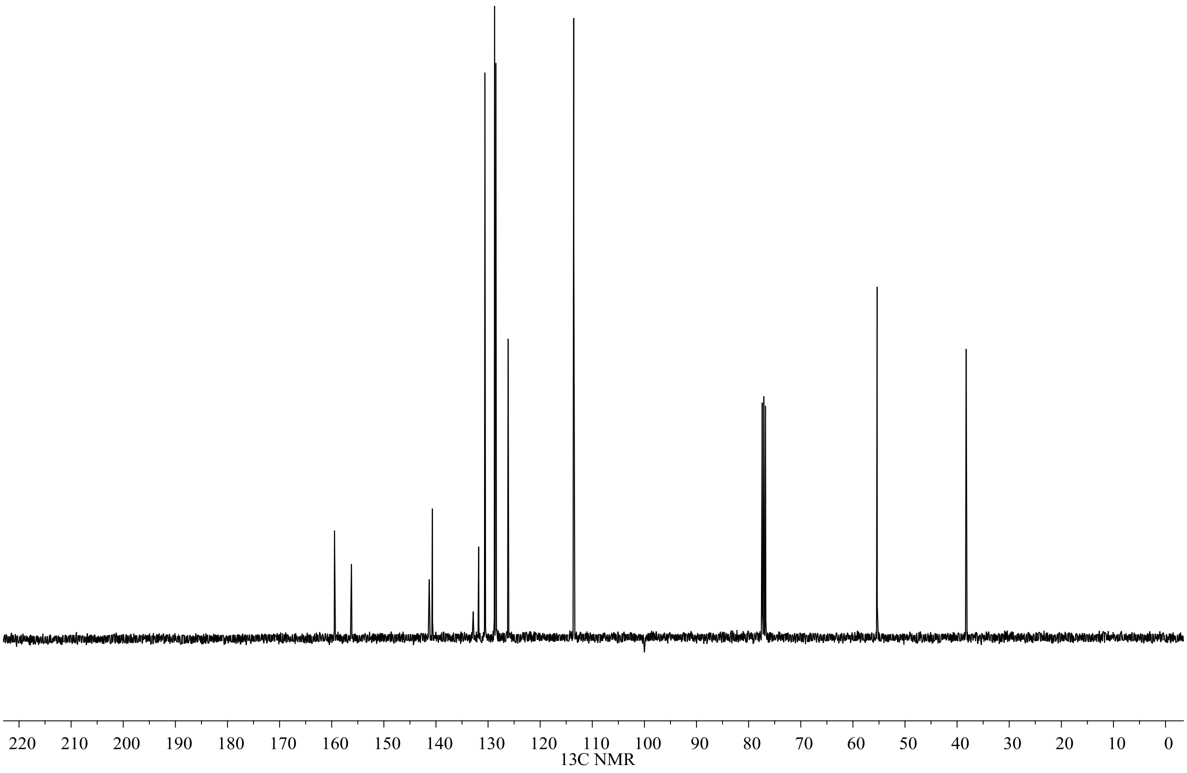

Supplement: Supplementary file 1 — miscellaneous_information [file anie0054-1642-SD1.pdf]
